# Preparation, COX-2 Inhibition and Anticancer Activity of Sclerotiorin Derivatives

**DOI:** 10.3390/md19010012

**Published:** 2020-12-29

**Authors:** Tao Chen, Yun Huang, Junxian Hong, Xikang Wei, Fang Zeng, Jialin Li, Geting Ye, Jie Yuan, Yuhua Long

**Affiliations:** 1 School of Chemistry, Guangzhou Key Laboratory of Analytical Chemistry for Biomedicine, South China Normal University, Guangzhou 510006, China; ct2020@m.scnu.edu.cn (T.C.); hjx2020@m.scnu.edu.cn (J.H.); xk20192421139@m.scnu.edu.cn (X.W.); zengfang@m.scnu.edu.cn (F.Z.); jialinli@m.scnu.edu.cn (J.L.); yegeting@m.scnu.edu.cn (G.Y.); 2 Department of Biochemistry, Zhongshan School of Medicine, Sun Yat-sen University, Guangzhou 510080, China; huangyun11910173@i.smu.edu.cn; 3 School of Basic Medical Sciences, Southern Medical University, Guangzhou 510515, China

**Keywords:** sclerotiorin derivatives, cytotoxic activity, COX-2 inhibition, molecular docking

## Abstract

The latest research has indicated that anti-tumor agents with COX-2 inhibitory activity may benefit their anti-tumor efficiency. A series of sclerotiorin derivatives have been synthesized and screened for their cytotoxic activity against human lung cancer cells A549, breast cancer cells MDA-MB-435 using the MTT method. Among them, compounds **3**, **7**, **12**, **13**, **15**, **17** showed good cytotoxic activity with IC_50_ values of 6.39, 9.20, 9.76, 7.75, 9.08, and 8.18 μM, respectively. In addition, all compounds were tested in vitro the COX-2 inhibitory activity. The results disclosed compounds **7**, **13**, **25** and sclerotiorin showed moderate to good COX-2 inhibition with the inhibitory ratios of 58.7%, 51.1%, 66.1% and 56.1%, respectively. Notably, compound **3** displayed a comparable inhibition ratio (70.6%) to the positive control indomethacin (78.9%). Furthermore, molecular docking was used to rationalize the potential of the sclerotiorin derivatives as COX2 inhibitory agents by predicting their binding energy, binding modes and optimal orientation at the active site of the COX-2. Additionally, the structure-activity relationships (SARS) have been addressed.

## 1. Introduction

Cancer has become one of the most important factors affecting human life and health in terms of incidence, mortality, and prevalence. In 2018, estimates for global statistics on cancer rates show that there were 18.1 million new cases and 9.6 million deaths. Lung cancer is the most frequent cancer and the leading cause of cancer death among males, followed by prostate and colorectal cancer. Among females, breast cancer is the most commonly diagnosed cancer and the leading cause of cancer death [1]. Cancer metastasis is the major reason of treatment failure and death [2,3]. As the research moves along, authoritative studies have shown that an inflammatory environment plays an important role in various stages of tumor development and affects the body response to chemotherapeutic agents [4,5]. Chronic inflammation is associated with tumor development, and inflammatory mediators are present in the tumor microenvironment, including cytokines, growth factors, reactive oxygen species and reactive nitrogen species [6,7,8]. These mediators also activate signaling molecules involved in inflammation and carcinogenesis, including nuclear transcription factor, inducible nitric oxide synthase and cyclooxygenase-2. All these factors together result in tumor initiation by increasing cell cycling, inhibiting tumor suppressor pathways and activating oncogenes [8,9,10]. 

COX-2 is a member of the cyclooxygenase family, and has long been a research focus in the treatment of inflammation. It is the key enzyme in the conversion of arachidonic acid to prostanoids, lipid mediators’ participation in multifarious physiological and pathological processes. COX-2 is known as an important enzyme in the inflammation process that has tumorigenesis function, and can promote cancer cells proliferation, migration and invasion [11,12]. Zoological and epidemiological studies have shown that COX-2 is closely associated with malignant tumors in recent years [13,14,15]. COX-2 over expression has been detected in lung cancer, colon cancer, stomach cancer, breast cancer and other tumors [16,17,18]. In lung cancer, studies show that COX-2 was highly expressed in all stages of NSCLS (non-small-cell lung cancer). Meanwhile, most of invasive and non-invasive lesions have shown elevated COX-2 expression when compared with nonmalignant tissue [19]. Concerning to colorectal cancer, studies have reported that COX-2 was over expressed in 85% and 50% of human colorectal carcinomas and adenomas, respectively. Immunohistochemical studies showed that COX-1 and trace COX-2 is expressed in normal smooth muscle cell and intestinal mucosal epithelial cell. However, COX-2 expression is dramatically increased in cancerous colorectal tissue compared with adjacent normal mucosa [20]. Studies indicated that COX-2 inhibitors aspirin and celecoxib can abrogate the stimulatory effects on lung cancer cell proliferation and migration by inhibition of COX-2 [21]. Moreover, celecoxib has been proved to inhibit growth and induce apoptosis of lung cancer cells [22]. Epidemiologic data demonstrated a role for aspirin in suppressing prostate carcinogenesis and suggested that inhibition of COX-2 via pharmacological means or regulation of its expression can limit the development of human prostate cancer [23]. Therefore, targeting the inhibition of COX-2 and its downstream pathways could be helpful for cancer therapy.

Marine creatures are natural treasures and have played important roles as sources of natural medicinal products [24,25]. Scientific researches showed that marine organisms derived marine natural products (MNPs) exhibited great potential for biological activities such as antifouling, antimalarial and anticancer, etc. [26,27,28]. Sclerotiorine was first isolated and identified from the mycelium of *Penicillium multicolor* G.M.P. in 1952 [29]. Its wide broad bioactivity makes it bound to get much more attention. Sclerotiorine and other azaphilones isolated from *Penicillium sclerotiorum* OUCMDZ-3839 displayed significant inhibitory activity against α-glycosidase and moderate bioactivity against H1N1 virus [30]. A series of sclerotioramine derivatives were synthesized and showed potent antifouling activity against the larval settlement of the barnacle *Balanus amphitrite* [31]. It is reported that sclerotiorin could substantially decrease the mycobacterial growth inside macrophages and with no cytotoxicity, suggesting that it has potential to supplement antibiotic therapy for tuberculosis (TB) [32]. Recently, evidence showed that sclerotiorin can provide clues for further searching on safer and effective entity against Alzheimer’s disease (AD) [33]. 

During our continuous search for biologically active marine natural compounds, we found an endophytic fungus SCNU-0016 from marine mangrove plant *Acanthus ilicifolius L.* could produce sclerotiorin in high productivity. Our primary research indicated sclerotiorin possesses COX-2 inhibition activity. The literature retrieval showed azaphilones can be antitumor agents [34]. In order to obtain dual targeted pharmaceutical compounds, since COX-2 selective inhibitors can both be anti-inflammatory candidates and benefit antitumor activity, we did the modification of sclerotiorin to afford the cytotoxic compounds with COX-2 inhibitory activity. Herein, we report synthesis, antitumor activity and COX-2 inhibition of a series of sclerotiorin derivatives.

## 2. Results and Discussion

### 2.1. Chemistry

**Scheme 1:**
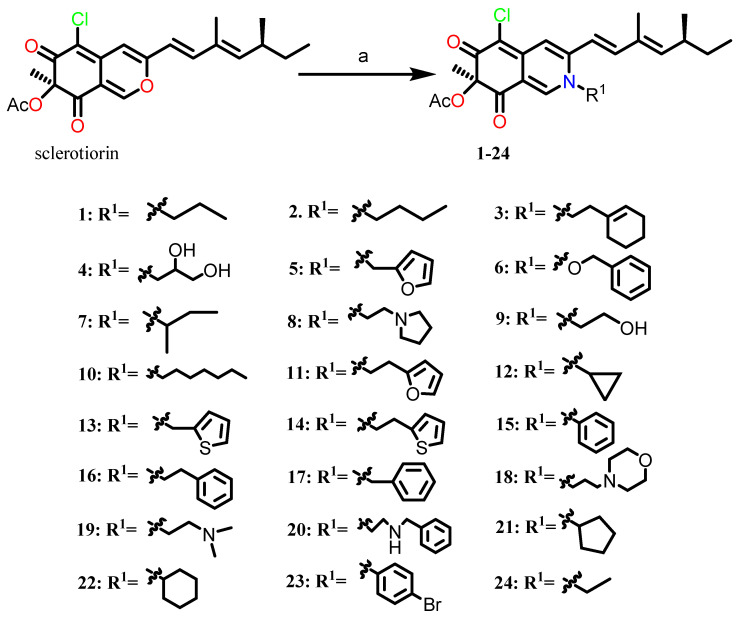
Reagents and conditions: (**a**) amine, DCM, r.t.

**Scheme 2:**
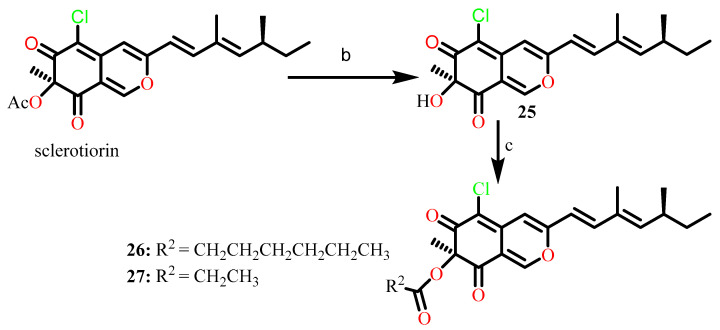
Reagents and conditions: (**b**) CH_3_ONa, CH_3_OH, 40 °C. (**c**) Pyridine, anhydride, 90 °C.

The parent compound sclerotiorin was extracted from the fungal *Penicillium sclerotiorum* SCNU-0016. The synthetic route of sclerotiorin derivatives **1**–**27** was shown in Scheme 1 and Scheme 2. Twenty-four amide-derivatives (**1**–**24**) have been successfully synthesized by one-step reaction of sclerotiorin with various amines in high yields (up to 90%). The derivative **25** was obtained by hydrolysis of sclerotiorin (yields up to 85%). Followed by esterification reaction of propionic anhydride and glutaric anhydride produced **26** and **27** in 82% and 78% yields, respectively. The resulting extract was subjected to silica gel column chromatography to obtain the pure products. The structures of target compounds **1**–**27** were confirmed by extensive spectroscopic methods including ^1^H NMR, ^13^C NMR, MS and HRMS.

### 2.2. Biological Studies

#### 2.2.1. Cytotoxic Activity of Sclerotiorin Derivatives 

The cytotoxic activity of all compounds was evaluated against A549 (human lung cancer) and MDA-MB-435 (breast cancer cells) by using the MTT method as described previously. 

As shown in Table 1, the four yellow pigments, sclerotiorin and the other three acyl changed derivatives **25**, **26**, **27,** present no cytotoxic activity on both cancer cell lines (IC_50_ > 50 μM). Compared with sclerotiorin, most of the amine modified sclerotiorin derivatives except **1** and **2** displayed moderate to fine cytotoxic activities against the MDA-MB-435 and A549 cell lines. Particularly noteworthy, compounds **3**, **7**, **12**, **13**, **15** and **17** showed excellent cytotoxic activities with IC_50_ values of 6.39, 9.20, 9.76, 7.75, 9.08 and 8.18 μM, respectively. Further SAR analysis can give the following clues: (1) a vinylogous γ-pyridone formed by the corresponding nitrogen atom substituted pyran nucleus of sclerotiorin increase the cytotoxicity; (2) a certain suitable bulky structure can obtain high cytotoxic activity, which was disclosed by comparing the IC_50_ values of compounds **1**, **2**, **24** to those of **7, 10, 12**; (3) from the observation of compounds **13**, **14, 15, 16, 17,** we can conclude one methylene connected aryl side chain have good effect on cytotoxic activity. In order to illustrate the underlying mechanisms for the cytotoxicity of these compounds, several rational deduces are summed up. Firstly, the amine modified sclerotiorin derivatives may be more efficiently served as a basic factor to change the acidic tumor microenvironment. In this respect, we can explain why the nitrogen substituted structures are more cytotoxic to tumor cells. Secondly, sclerotiorin derivatives possess α, β-unsaturated ketone skeleton and could be Michael receptor. Cytotoxic activity of these compounds may be attributed to the Michael addition reactions of sclerotiorin derivatives with active nucleophilic group in the biomolecules, such as amino acids, nucleic acids and other compounds in the tumor cells to irreversibly affect the functions of the biomolecules or regulate the cellular signal pathway. Thirdly, the literature addressing tumor cells always showed some high expression of cellular factors, the sclerotiorin derivatives may exhibit cytotoxicity by inhibiting some of them, such as the COX-2. Further COX-2 inhibitory activity for these compounds was conducted as following.

#### 2.2.2. In Vitro Primary Screen COX-2 Inhibitory Activity

In recent years, COX-2 inhibitors become a new target and hotspot for anticancer drug research and get a lot of attention. For further investigations the biological activity of semi-synthetic analogs of sclerotiorin, all compounds were primarily screened the COX-2 inhibitory activity at a concentration of 20 μM in vitro.

**Figure 1:**
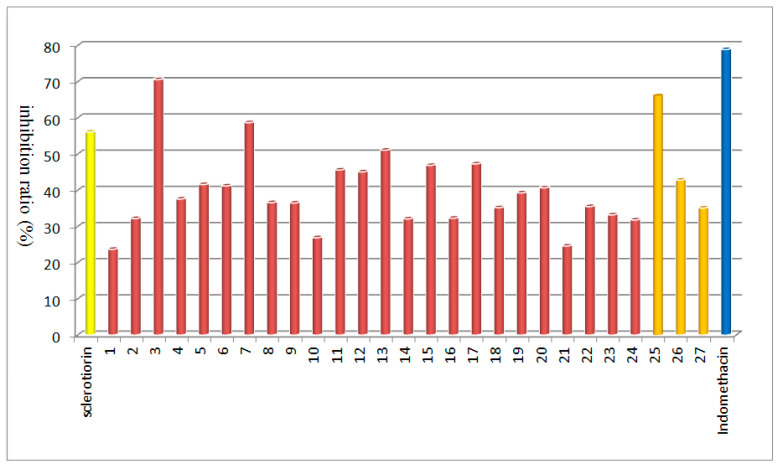
Inhibition ratio of compounds **1**–**27** to COX-2 in vitro.

As shown in Figure 1, most of semi-synthetic derivatives and parent compound sclerotiorin displayed good inhibitory activity for COX-2. Among all the derivatives, compound **3** displayed perfect COX-2 inhibition with a ratio of 70.6%, which is comparable to the positive control indomethacin (78.9%). Furthermore, compounds **7**, **13**, **15**, **17**, **25** and sclerotiorin showed moderate COX-2 inhibitory ratio of 58.7%, 51.1%, 46.7%, 47.3%, 66.1% and 56.1%, respectively. More interestingly, compared to the parent compound sclerotiorin, the inhibitory activity of the esterlysis derivative **25** increased from 56.1% to 66.1%. However, **26** and **27**, the esterification product of **25**, displayed low COX-2 inhibitory ratio of 42.8%, 35.1%, respectively. 

Based on the above results, observation was that the COX-2 inhibitory activity of sclerotiorin derivatives were associated with the cytotoxic activity. Overall, the cytotoxic activity of the compounds were positively correlated with the inhibitory activity of COX-2. Out of 28 tested compounds, **3**, **7**, **13**, **15** and **17** showed both cytotoxic activity against two cancer cell lines and potential COX-2 inhibitory activity. Particularly, compound **3** was found to be most potent derivative with high cytotoxic activity against the breast cancer cells and superior COX-2 inhibitory activity. Otherwise, compounds **25**, **26**, **27** and parent compound sclerotiorin were exhibiting good COX-2 inhibitory activity, but almost have no cytotoxic activity. This find suggests sclerotiorin and its esterlysis product **25** deserve intensive investigation on anti-inflammatory activity based on COX-2 inhibition selectivity.

#### 2.2.3. Molecular Docking Studies

**Figure 2:**
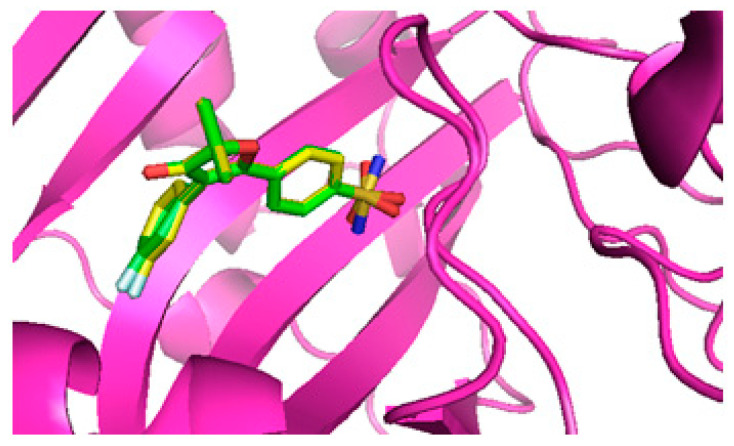
Comparison between the docked pose of 949 (yellow) as produced by docking experiment and the co-crystallized ligand of this inhibitor within COX-2 (green).

The COX-2 inhibitory effects of compound **3** led us to perform molecular docking studies to insight understand the ligand-protein interactions in detail, and dock simulations in COX-2 (PDB ID: 5GMN) [35] were carried out in AutoDock4.2.6 [36]. Docking procedure was validated by docking of 949 (the co-crystallized ligand of COX-2 protein) in the active site of COX-2 and root-mean-square deviation (RMSD) of 0.13 Å to the X-ray structure (Figure 2).

**Figure 3:**
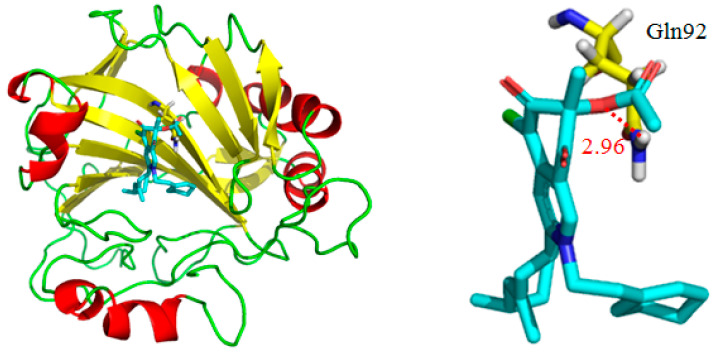
Compound **3** (cyan) docked into the active site of COX-2.

**Figure 4:**
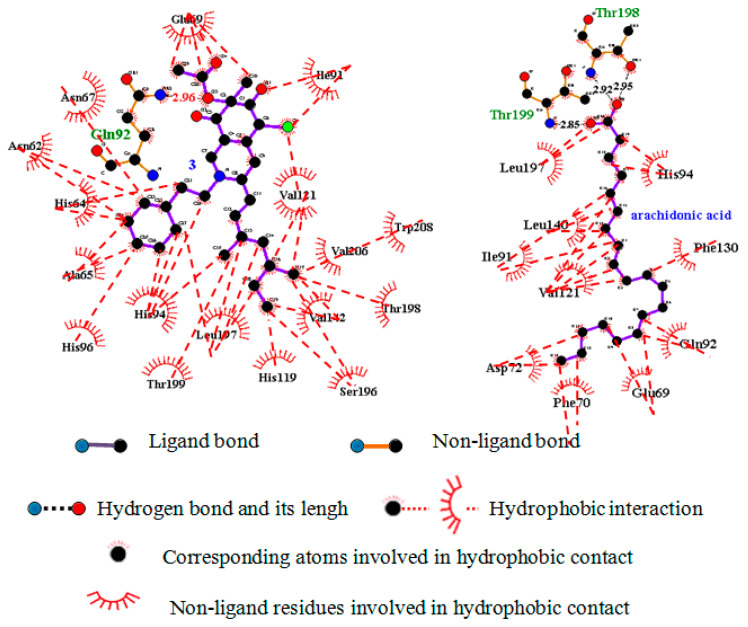
Two-dimensional (2D) diagram of the binding pose of **3** and arachidonic acid in the active site of COX-2.

**Figure 5:**
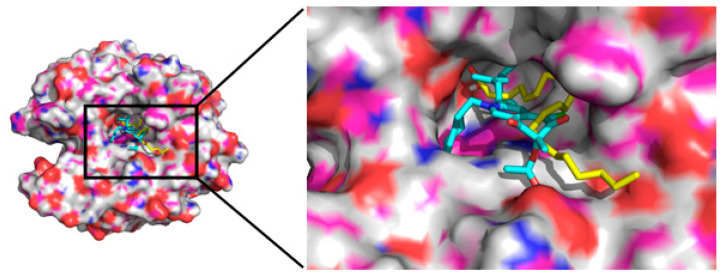
Model showing the placement of **3** (cyan) and arachidonic acid (yellow) in the active site pocket of COX-2.

Compound **3** was docked into the active site of COX-2 and the interaction energy of 8.24 kcal/mol was obtained. One hydrogen bonding interaction between the ester group of compound **3** with Gln92 (2.96 Å) of COX-2 active site was observed (Figure 3). It is clearly visible in Figure 4 that arachidonic acid and compound **3** were located deeply inside the same pocket of COX-2. Three hydrogen bonding interactions between carboxyl of arachidonic acid with Thr198 (2.92 Å, 2.95 Å) and Thr199 (2.85 Å) of COX-2 active site were observed respectively (Figure 5). The hydrophilic groups of compound **3** including carbonyl and ester group were inclined to via a hydrogen bonding interactions with the hydrophilic amino acid of the outside of the COX-2 protein active pocket. However, alkyl chain and cyclohexene of compound **3** is easier access to the interior of hydrophobic pocket by means of hydrophobic action, which is blocking the binding site of arachidonic acid in COX-2 enzyme to some extent. This may be the reason why the compound **3** showed a perfect inhibitory activity to COX-2 cyclooxygenase (Figure 5).

**Figure 6:**
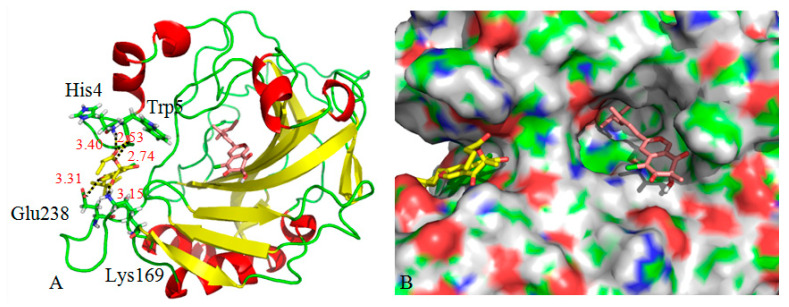
(**A**) 3D diagram of the binding model of **25** (brown) and **26** (yellow) in the active site of COX-2. (**B**) Model showing the placement of **25** (brown) and **26** (yellow) in the active site pocket of COX-2.

Compound **25** was docked into the active site of COX-2 and the interaction energy of 7.66 kcal/mol was obtained. Unexpectedly, it displayed no H-bonding interaction with the amino acid residue of COX-2 protein. Compound **26** was docked into the active site of COX-2 and the interaction energy of 6.26 kcal/mol was obtained. Three hydrogen bonding interactions between ester group of **26** with His4 (3.40 Å) and Trp5 (2.53 Å, 2.74 Å) of COX-2 active site was observed. One hydrogen bonding interaction between carbonyl group of **26** with Lys169 (3.15 Å) of COX-2 active site was observed. Additionally, Compound **26** showed H-bond interactions of 3.31 Å between its oxygen atom in the ring and Glu238 residues of the COX-2 active site (Figure 6A). However, we found that compound **26** did not located deeply inside the hydrophobic pocket of COX-2 protein as compound **25** did, but docked on the surface of the protein by hydrogen bonding (Figure 6B). Consequently, we deduce that the presence of ester group can resulted in lower activity of compound **26**.

## 3. Materials and Methods

### 3.1. Chemistry

#### 3.1.1. General Information

Proton and carbon NMR spectra were recorded on Bruker AVANCE NEO 600 MHz spectrometer (Bruker BioSpin, Switzerland). The spectra obtained in CDCl_3_ were referenced to the residual solvent peak. Chemical shifts (d) are reported in parts per million (ppm) relative to residual undeuterated solvent as an internal reference. HR-ESI-MS data were measured on a Thermo Fisher Scientific Q-TOF mass spectrometer (Thermo Fisher Scientific, Waltham, MA, USA). Silica gel (100–200 and 200–300 mesh) (Qingdao Haiyang Chemical Co., Ltd., Qingdao, China) was used for column chromatography. TLC silica gel GF254 plates (Yantai Zi Fu Chemical Co., Ltd., Yantai, China) and TLC silica gel 60 F254 (MERCK CHEMICALS (SHANGHAI) CO, LTD) were used for thin layer chromatography.

#### 3.1.2. Fungal Materials.

The fungus SCNU-0016 used in the study was isolated from fresh fruit of the mangrove plant *Acanthus ilicifolius L*, which was collected in October 2019 from Hailing island Mangrove Nature Reserve in Guangdong province, China. The fungal isolation was conducted as following. Initially, the plant fruit was washed with sterile water and surface-sterilized in a 100 mL beaker with 75% ethanol for 1 min. This was followed by dipping the sample into 5% sodium hypochlorite for 1 min, then the plant parts were rinsed with sterile water and cut into 3 mm sections and plated on potato dextrose agar (PDA, potatoes 300 mg/mL, dextrose 20 mg/mL, agar 15 mg/mL, chloramphenicol 1 mg/mL) with penicillin (100 units/mL) and streptomycin (0.8 mg/ mL). The plates were incubated at 25 ± 1 °C. The endophytic fungal strains were isolated by routine microbiological. The fungal isolates were numbered and stored at 4 °C in triplicate on PDA slants. Fungal identification was carried out using a molecular biological protocol by DNA amplication and sequencing of the ITS region [37]. The sequence data of the fungal strain have been deposited at Gen Bank with accession no. MW-309502. A BLAST search result showed that the sequence was the most similar (100%) to the sequence of *P. sclerotiorum*. A voucher strain was deposited in School of Chemistry, South China Normal University, Guangzhou, China, with the access code SCNU-F0016.

#### 3.1.3. Fungal Culture and Sclerotiorin Extraction

The fungal strain *P. sclerotiorum* SCNU-0016 was cultured on autoclaved potato liquid-substrate media (one-hundred Erlenmeyer flasks (1000 mL); each containing 400 mL of potato liquid medium, 8 g of glucose and 1.2 g artificial sea salts) at room temperature under static conditions and daylight for 28 days. Following incubation, pancake thallus grew on top of the potato liquid media. Then the air dried fungal were soaked in the solvent MeOH/CH_2_Cl_2_ (v:v, 1:1). Extracts were filtered and concentrated under reduced pressure to yield 820 g of residue. The residue was fractionated by column chromatography on silica gel by eluting with a gradient of EtOAc/petroleum ether from 1:10 to 1:1 give five fractions (Fr.1-Fr.5). sclerotiorin was extracted successfully by elution with EtOAc/petroleum ether (v:v, 1:10). sclerotiorin appear good solubility in the EtOAc/petroleum ether solution. Then removal of the solvent afforded 7.82 g of yellow powder.

#### 3.1.4. General Procedure for Synthesis of Sclerotioramine Derivatives **1**–**24**

A mixture of sclerotiorin (39 mg, 0.10 mmol) and varied amines (0.12 mmol) in anhydrous dichloromethane (2 mL) was stirred at room temperature until the sclerotiorin was disappeared. The resulting mixture was extracted with EtOAc (3 × 5 mL) and the organic phase was washed with saturated brine (3 × 15 mL). Then the organic phase was dried with MgSO_4_ and concentrated in vacuo. The crude product was purified by column chromatography with eluent of EA/PE (5:1, *v*/*v*) to give the pure compound. All the synthesized compounds were identified by MS and NMR spectra, please see Figures S1–S79 in the supplementary materials. 

Compound **1**

(*R*)-5-chloro-3-((*S*, 1*E*, 3*E)*-3,5-dimethylhepta-1, 3-dien-1-yl)-7-methyl-6, 8-dioxo-2-propyl-2, 6, 7, 8-tetrahydroisoquinolin-7-yl acetate. Red solid, the yield of 90%; m.p. 230.1–232.2 °C. ${\left[\alpha \right]}_{D}^{25}$ +13.2 (*c* 0.02, MeOH); UV (MeOH) λmax (log ε): 234 (3.13), 376 (3.03), 488 (1.68) nm; IR (KBr) *ν*_max_: 2956, 2922, 1743, 1708, 1590, 1491, 1368, 1251, 1190, 1092, 853 cm^−1^. ^1^H NMR (600 MHz, CDCl_3_) δ_H_ 7.75 (s, 1H), 7.02 (s, 1H), 6.96(d, *J* = 15.4 Hz, 1H), 6.12 (d, *J* = 15.4 Hz, 1H), 5.70 (d, *J* = 9.7 Hz, 1H), 3.89–3.72 (m, 2H), 2.59–2.34 (m, 1H), 2.16 (s, 3H), 1.85 (s, 3H), 1.80 (dd, *J* = 14.9, 7.4 Hz, 2H), 1.55 (s, 3H), 1.48–1.40 (m, 1H), 1.39–1.31 (m, 1H), 1.01 (dd, *J* = 15.3 7.2 Hz, 6H), 0.88 (t, *J* = 7.4 Hz, 3H). ^13^C NMR (150 MHz, CDCl_3_) δ_C_ 193.9, 184.2, 170.1, 148.2, 147.8, 145.0, 144.6, 141.1, 131.6, 114.6, 114.5, 111.7, 102.2, 84.8, 55.8, 35.1, 30.0, 23.6, 23.2, 20.3, 20.2, 12.6, 12.0, 10.9. LRMS (EI) *m/z* 432 [M]^+^.

Compound **2**

(*R*)-2-butyl-5-chloro-3-((*S*, 1*E*, 3*E*)-3,5-dimethylhepta-1, 3-dien-1-yl)-7-methyl-6, 8-dioxo-2, 6, 7, 8-tetrahydroisoquinolin-7-yl acetate. Red solid, the yield of 92%; m.p. 216.3–218.5 °C. ${\left[\alpha \right]}_{D}^{25}$ +13.6 (*c* 0.02, MeOH); UV (MeOH) λmax (log ε): 238 (3.10), 378 (3.09), 485 (1.78) nm; IR (KBr) *ν*_max_: 2953, 2921, 1743, 1708, 1590, 1493, 1368, 1252, 1190, 1092, 851 cm^−1^. ^1^H NMR (600 MHz, CDCl_3_) δ_H_ 7.74 (s, 1H), 7.02 (s, 1H), 6.96 (d, *J* = 15.4 Hz, 1H), 6.13 (d, *J* = 15.4 Hz, 1H), 5.70 (d, *J* = 9.7 Hz, 1H), 3.94–3.76 (m, 2H), 2.59–2.39 (m, 1H), 2.16 (s, 3H), 1.84 (s, 3H) 1.74 (s, 2H), 1.54 (s, 3H), 1.48–1.31 (m, 4H), 1.02 (d, *J* = 6.6 Hz, 3H), 0.98 (t, *J* = 7.4 Hz, 3H), 0.88 (t, *J* = 7.4 Hz, 3H). ^13^C NMR (150 MHz, CDCl_3_) δ_C_ 194.1, 184.3, 170.2, 148.3, 147.9, 145.1, 144.7, 141.2, 131.7, 114.8, 114.6, 111.8, 102.3, 84.9, 54.2, 35.2, 32.3, 30.2, 23.4, 20.4, 20.4, 19.8, 13.7, 12.7, 12.1. LRMS (EI) *m/z* 446 [M]^+^.

Compound **3**

(*R*)-5-chloro-2-(2-(cyclohex-1-en-1-yl)ethyl)-3-((*S*, 1*E*, 3*E*)-3,5-dimethylhepta-1, 3-dien-1-yl)-7-methyl-6, 8-dioxo-2, 6, 7, 8-tetrahydroisoquinolin-7-yl acetate. Red solid, the yield of 92%; m.p. 180.6–182.8 °C. ${\left[\alpha \right]}_{D}^{25}$ +14.2 (*c* 0.02, MeOH); UV (MeOH) λmax (log ε): 235 (3.11), 382 (3.28), 491 (1.66) nm; IR (KBr) *ν*_max_: 2965, 2920, 1743, 1703, 1600, 1491, 1368, 1288, 1248, 1180, 1089, 953 cm^−1^. ^1^H NMR (600 MHz, CDCl_3_) δ_H_ 7.64 (s, 1H), 7.01 (s, 1H), 6.96 (d, *J* = 15.4 Hz, 1H), 6.16 (d, *J* = 15.4 Hz, 1H), 5.70 (d, *J* = 9.7 Hz, 1H), 5.41 (s, 1H), 4.05–3.74 (m, 2H), 2.56–2.38 (m, 1H), 2.31 (t, *J* = 7.1 Hz, 2H), 2.16 (s, 3H), 2.00–1.87 (m, 4H), 1.85 (s, 3H), 1.63 (dd, *J* = 10.1, 4.1 Hz, 2H), 1.56–1.48 (m, 5H), 1.47–1.40 (m, 1H), 1.35 (dd, *J* = 14.3, 6.9, 1H), 1.02 (d, *J* = 6.6 Hz, 3H), 0.88 (t, *J* = 7.4 Hz, 3H). ^13^C NMR (150 MHz, CDCl_3_) δ_C_ 194.0, 184.3, 170.1, 148.2, 147.8, 145.1, 144.7, 141.5, 131.9, 131.7, 127.0, 114.7, 114.4, 111.7, 102.1, 84.9, 53.1, 38.6, 35.2, 30.2, 28.4, 25.4, 23.4, 22.7, 22.0, 20.4, 20.4, 12.7, 12.1. HRMS (ESI) for [M + H]^+^: calcd for C_29_H_37_ClNO_4_: 498.23332. Found: 498.24115.

Compound **4**

(7*R*)-5-chloro-2-(2, 3-dihydroxypropyl)-3-((*S*, 1*E*, 3*E*)-3,5-dimethylhepta-1, 3-dien-1-yl)-7-methyl-6, 8-dioxo-2, 6, 7, 8-tetrahydroisoquinolin-7-yl acetate. Red solid, the yield of 88%; m.p. 152.8–154.3 °C. ${\left[\alpha \right]}_{D}^{25}$ +14.5 (*c* 0.02, MeOH); UV (MeOH) λmax (log ε): 236 (3.09), 379 (3.10), 488 (1.64) nm; IR (KBr) *ν*_max_: 3537, 2958, 2926, 1733, 1701, 1581, 1489, 1242, 1154, 858 cm^−1^. ^1^H NMR (600 MHz, CD_3_OD) δ_H_ 8.15 (s, 1H), 7.20 (s, 1H), 7.11 (d, *J* = 15.4 Hz, 1H), 6.64 (d, *J* = 15.4 Hz, 1H), 5.79 (d, *J* = 9.7 Hz, 1H), 4.46 (dd, *J* = 14.4, 2.5 Hz, 1H), 3.94 (dd, *J* = 14.4, 9.2 Hz, 1H), 3.91–3.84 (m, 1H), 3.63 (dd, *J* = 11.1, 4.7 Hz, 1H), 3.53 (dd, *J* = 11.1, 6.7 Hz, 1H), 2.56–2.38 (m, 1H), 2.13 (d, *J* = 20 Hz, 3H), 1.92 (d, *J* = 0.9 Hz, 3H), 1.51 (d, *J* = 5.2 Hz, 3H), 1.50–1.46 (m, 1H), 1.41–1.34 (m, 1H), 1.05 (dd, *J* = 6.7, 2.1 Hz, 3H), 0.91 (t, *J* = 7.4 Hz, 3H). ^13^C NMR (150 MHz, CD_3_OD) δ_C_ 195.1, 185.5, 171.6, 151.8, 148.7, 148.3, 146.5, 144.9, 134.0, 117.4, 116.1, 112.3, 101.2, 86.2, 71.7, 64.5, 58.3, 36.2, 31.2, 23.8, 20.6, 20.2, 12.8, 12.4. HRMS (ESI) for [M − H]^−^: calcd for C_24_H_29_ClNO_6_: 462.17617. Found: 462.16899.

Compound **5**

(*R*)-5-chloro-3-((*S*, 1*E*, 3*E*)-3,5-dimethylhepta-1, 3-dien-1-yl)-2-(furan-2-ylmethyl)-7-methyl-6, 8-dioxo-2, 6, 7, 8-tetrahydroisoquinolin-7-yl acetate. Red solid, the yield of 96%; m.p. 160.0–161.5 °C. ${\left[\alpha \right]}_{D}^{25}$ +16.6 (*c* 0.02, MeOH); UV (MeOH) λmax (log ε): 240 (3.16), 380 (3.06), 496 (1.66) nm; IR (KBr) *ν*_max_: 2960, 2928, 1735, 1703, 1595, 1497, 1371, 1256, 1144, 1104, 1083, 860 cm^−1^. ^1^H NMR (600 MHz, CDCl_3_) δ_H_ 7.84 (s, 1H), 7.45–7.41 (m, 1H), 6.97 (s, 1H), 6.90 (d, *J* = 15.3Hz, 1H), 6.42–6.37 (m, 2H), 6.33 (d, *J* = 15.4 Hz, 1H), 5.67 (d, *J* = 9.8 Hz, 1H), 4.97 (d, *J* = 4.8 Hz, 2H), 2.52–2.40 (m, 1H), 2.14 (s, 3H), 1.84 (d, *J* = 0.9 Hz, 3H), 1.53 (s, 3H), 1.46–1.40 (m, 1H), 1.36–1.28 (m, 1H), 1.01 (d, *J* = 6.7 Hz, 3H), 0.87 (t, *J* = 7.4 Hz, 3H). ^13^C NMR (150 MHz, CDCl_3_) δ_C_ 193.8, 184.5, 170.2, 148.3, 148.1, 147.0, 145.1, 144.5, 144.2, 141.1, 131.9, 115.1, 115.0, 111.6, 111.1, 110.6, 102.7, 85.0, 50.3, 35.1, 30.1, 23.3, 20.4, 20.3, 12.7, 12.1. HRMS (ESI) for [M + H]^+^: calcd for C_26_H_29_ClNO_5_: 470.16560. Found: 470.17326.

Compound **6**

(*R*)-2-(benzyloxy)-5-chloro-3-((*S*, 1*E*, 3*E*)-3,5-dimethylhepta-1, 3-dien-1-yl)-7-methyl-6, 8-dioxo-2, 6, 7, 8-tetrahydroisoquinolin-7-yl acetate. Red solid, the yield of 96%; m.p. 166.6–168.3 °C. ${\left[\alpha \right]}_{D}^{25}$ +14.3 (*c* 0.02, MeOH); UV (MeOH) λmax (log ε): 248 (3.09), 386 (3.06), 491 (1.67) nm; IR (KBr) *ν*_max_: 3080, 2964, 2929, 1723, 1703, 1611, 1487, 1375, 1252, 1221, 1148, 1080, 952, 781 cm^−1^. ^1^H NMR (600 MHz, CDCl_3_) δ_H_ 7.82 (s, 1H), 7.44 (d, *J* = 7.1 Hz, 3H), 7.36 (dd, *J* = 7.6, 1.7 Hz, 2H), 7.08 (d, *J* = 15.9 Hz, 1H), 7.00 (s, 1H), 6.29 (d, *J* = 15.9 Hz, 1H), 5.74 (d, *J* = 9.7 Hz, 1H), 5.06 (dd, *J* = 25.6, 10.1 Hz, 2H), 258–2.43 (m, 1H), 2.17 (s, 3H), 1.83 (d, *J* = 0.7 Hz, 3H), 1.53 (s, 3H), 1.48–1.40 (m, 1H), 1.38–1.34 (m, 1H), 1.03 (d, *J* = 6.6 Hz, 3H), 0.90 (d, *J* = 7.4 Hz, 3H). ^13^C NMR (150 MHz, CDCl_3_) δ_C_ 193.15, 184.41, 170.22, 149.11, 145.86, 144.88, 143.54, 137.20, 132.12, 131.62, 130.58, 130.27, 129.37, 128.98, 113.77, 112.08, 109.43, 103.14, 84.74, 81.96, 65.71, 35.28, 30.17, 29.83, 23.29, 20.35, 12.63, 12.13. HRMS (ESI) for [M + H]^+^: calcd for C_28_H_31_ClNO_5_: 496.18125. Found: 496.18889.

Compound **7**

(*R*)-5-chloro-3-((*S*, 1*E*, 3*E*)-3,5-dimethylhepta-1, 3-dien-1-yl)-7-methyl-6, 8-dioxo-2-(2-(pyrrolidin-1-yl)ethyl)-2, 6, 7, 8-tetrahydroisoquinolin-7-yl acetate. Red solid, the yield of 96%; m.p. 145.5–147.6 °C. ${\left[\alpha \right]}_{D}^{25}$ +16.1 (*c* 0.02, MeOH); UV (MeOH) λmax (log ε): 246 (3.09), 379 (3.00), 488 (1.64) nm; IR (KBr) *ν*_max_: 2958, 2922, 1748, 1708, 1591, 1491, 1365, 1252, 1190, 1092, 860 cm^−1^. ^1^H NMR (600 MHz, CDCl_3_) δ_H_ 7.78 (s, 1H), 7.00 (s, 1H), 6.95 (d, *J* = 15.3 Hz, 1H), 6.23 (d, *J* = 15.3 Hz, 1H), 5.70 (d, *J* = 9.8 Hz, 1H), 3.98 (s, 2H), 2.84 (s, 2H), 2.61 (s, 4H), 2.53–2.43 (m, 1H), 2.17 (s, 3H), 1.86 (s, 3H), 1.81 (s, 4H), 1.54 (s, 3H), 1.49–1.40 (m, 1H), 1.38–1.34 (m, 1H), 1.03 (d, *J* = 6.6 Hz, 3H), 0.90 (d, *J* = 7.4 Hz, 3H). ^13^C NMR (150 MHz, CDCl_3_) δ_C_ 194.0, 184.4, 170.2, 149.2, 147.6, 145.0, 144.5, 136.5, 131.6, 115.4, 115.3, 112.7, 102.3, 84.9, 58.8, 35.1, 30.1, 30.0, 23.3, 20.9, 20.7, 20.4, 20.3, 12.7, 12.1, 10.8, 10.8. HRMS (ESI) for [M + H]^+^: calcd for C_27_H_36_ClN_2_O_4_: 487.22854. Found: 487.23595.

Compound **8**

(7*R*)-2-(sec-butyl)-5-chloro-3-((*S*, 1*E*, 3*E*)-3,5-dimethylhepta-1, 3-dien-1-yl)-7-methyl-6, 8-dioxo-2, 6, 7, 8-tetrahydroisoquinolin-7-yl acetate. Red solid, the yield of 93%; m.p. 164.4–466.8 °C. ${\left[\alpha \right]}_{D}^{25}$ +16.8 (*c* 0.02, MeOH); UV (MeOH) λmax (log ε): 256 (3.05), 386 (2.98), 498 (1.53) nm; IR (KBr) *ν*_max_: 2964, 2920, 1736, 1701, 1599, 1497, 1370, 1252, 1200, 1141, 1084, 861 cm^−1^. ^1^H NMR (600 MHz, CDCl_3_) δ_H_ 7.83 (s, 1H), 6.95 (s, 1H), 6.87 (d *J* = 15.3 Hz, 1H), 6.13 (d, *J* = 15.3 Hz, 1H), 5.67 (d, *J* = 9.7 Hz, 1H), 4.23 (dd, *J* = 13.8, 6.9 Hz, 1H), 2.53–2.41 (m, 1H), 2.17 (s, 3H), 1.85 (s,3H), 1.80 (dd, *J* = 14.8, 7.4 Hz, 2H), 1.56 (s, 3H), 1.47–1.42 (m, 4H), 1.38–1.33 (m, 1H), 1.02 (d, *J* = 6.6 Hz, 3H), 0.93 (t, *J* = 7.3 Hz, 3H), 0.88 (t, *J* = 7.4 Hz, 3H). ^13^C NMR (150 MHz, CDCl_3_) δ_C_ 194.0, 184.5, 170.2, 148.4, 147.9, 145.1, 144.6, 142.2, 131.7, 114.6, 114.4, 111.7, 102.3, 84.9, 54.0, 53.4, 51.5, 35.2, 30.1, 29.8, 23.4, 20.4, 20.3, 12.8, 12.1. HRMS (ESI) for [M + H]^+^: calcd for C_25_H_33_ClNO_4_: 446.20199. Found: 446.20947.

Compound **9**

(*R*)-5-chloro-3-((*S*, 1*E*, 3*E*)-3, 5-dimethylhepta-1, 3-dien-1-yl)-2-(2-hydroxyethyl)-7-methyl-6, 8-dioxo-2, 6, 7, 8-tetrahydroisoquinolin-7-yl acetate. Red solid, the yield of 88%; m.p. 180.2–182.8 °C. ${\left[\alpha \right]}_{D}^{25}$ +15.8 (*c* 0.02, MeOH); UV (MeOH) λmax (log ε): 233 (3.06), 389 (3.18), 498 (1.64) nm; IR (KBr) *ν*_max_: 3535, 2950, 2926, 1733, 1706, 1581, 1484, 1242, 1155, 866 cm^−1^. ^1^H NMR (600 MHz, CDCl_3_) δ_H_ 7.93 (s, 1H), 7.05 (s, 1H), 6.94 (d, *J* = 15.3 Hz, 1H), 6.28 (d, *J* = 15.4 Hz, 1H), 5.70 (d, *J* = 9.7 Hz, 1H), 4.04 (d, *J* = 5.4 Hz, 2H), 3.91 (d, *J* = 4.6 Hz, 2H), 2.46 (d, *J* = 8.6 Hz, 1H), 2.14 (s, 3H), 1.84 (s, 3H), 1.53 (s, 3H), 1.46–1.42 (m, 1H), 1.36–1.30 (m, 1H), 1.01 (d, *J* = 6.6 Hz, 3H), 0.87 (t, *J* = 7.4 Hz, 3H). LRMS (EI) m/z 434 [M]^+^.

Compound **10**

(*R*)-5-chloro-3-((S, 1E, 3E)-3, 5-dimethylhepta-1, 3-dien-1-yl)-2-heptyl-7-methyl-6, 8-dioxo-2, 6, 7, 8-tetrahydroisoquinolin-7-yl acetate. Red solid, the yield of 93%; m.p. 95.6–97.3 °C. ${\left[\alpha \right]}_{D}^{25}$ +13.9 (*c* 0.02, MeOH); UV (MeOH) λmax (log ε): 246 (3.05), 389 (3.20), 488 (1.64) nm; IR (KBr) *ν*_max_: 2966, 2928, 1748, 1705, 1591, 1493, 1368, 1252, 1190, 1093, 862 cm^−1^. ^1^H NMR (600 MHz, CDCl_3_) δ_H_ 7.74 (s, 1H), 7.01 (s, 1H), 6.95 (d, *J* = 15.3 Hz, 1H), 6.12 (d, *J* = 15.4 Hz, 1H), 5.70 (d, *J* = 9.8 Hz, 1H), 3.82 (td, *J* = 7.2, 2.9 Hz, 2H), 2.52–2.37 (m, 1H), 2.16 (s, 3H), 1.84 (s, 3H), 1.77–1.72 (m, 2H), 1.54 (s, 3H), 1.46–1.42 (m, 1H), 1.37–1.25 (m, 9H), 1.02 (d, *J* = 6.7 Hz, 3H), 088 (t, *J* = 7.3 Hz, 6H). ^13^C NMR (150 MHz, CDCl_3_) δ_C_ 194.1, 184.4, 170.2, 148.2, 147.9, 145.0, 144.7, 141.1, 131.7, 114.8, 114.7, 111.8, 102.2, 84.9, 54.5, 35.2, 31.6, 30.3, 30.1, 28.8, 26.4, 23.4, 22.6, 20.4, 20.3, 14.1, 12.7, 12.1. HRMS (ESI) for [M + H]^+^: calcd for C_28_H_39_ClNO_4_: 488.24894. Found: 488.25679.

Compound **11**

(*R*)-5-chloro-3-((*S*, 1*E*, 3*E*)-3, 5-dimethylhepta-1, 3-dien-1-yl)-2-(2-(furan-2-yl)ethyl)-7-methyl-6, 8-dioxo-2, 6, 7, 8-tetrahydroisoquinolin-7-yl acetate. Red solid, the yield of 90%; m.p. 130.3–132.8 °C. ${\left[\alpha \right]}_{D}^{25}$ +15.9 (*c* 0.02, MeOH); UV (MeOH) λmax (log ε): 256 (3.03), 379 (3.16), 493 (1.54) nm; IR (KBr) *ν*_max_: 2966, 2923, 1738, 1703, 1595, 1497, 1372, 1256, 1148, 1104, 1083, 869 cm^−1^. ^1^H NMR (600 MHz, CDCl_3_) δ_H_ 7.59 (s, 1H), 7.33 (d, *J* = 1.6 Hz, 1H), 6.96 (d, *J* = 15.3 Hz, 1H), 6.91 (d, *J* = 15.3 Hz, 1H), 6.28 (dd, *J* = 2.9, 2.0 Hz, 1H), 6.09 (d, *J* = 3.1 Hz, 1H), 5.98 (d, *J* = 15.3 Hz, 1H), 5.68 (d, *J* = 9.7 Hz, 1H), 4.17–4.05 (m, 2H), 3.22–2.98 (m, 2H), 2.47 (dt, *J* = 8.0, 7.3 Hz, 1H), 2.16 (s, 3H), 1.83 (s, 3H), 1.53 (s, 3H), 1.46–1.42 (m, 1H), 1.37–1.31 (m, 1H), 1.02 (d, *J* = 6.6 Hz, 3H), 0.88 (t, *J* = 7.4 Hz, 3H). ^13^C NMR (150 MHz, CDCl_3_) δ_C_ 193.8, 184.5, 170.2, 149.4, 148.1, 148.0, 145.2, 144.5, 142.7, 141.0, 131.8, 114.9, 114.5, 111.5, 110.9, 108.6, 102.5, 84.9, 52.8, 35.2, 30.2, 29.0, 23.3, 20.4, 20.4, 12.7, 12.1. HRMS (ESI) for [M + H]^+^: calcd for C_27_H_31_ClNO_5_: 484.18125. Found: 484.18897.

Compound **12**

(*R*)-5-chloro-2-cyclopropyl-3-((*S*, 1*E*, 3*E*)-3, 5-dimethylhepta-1, 3-dien-1-yl)-7-methyl-6, 8-dioxo-2, 6, 7, 8-tetrahydroisoquinolin-7-yl acetate. Red solid, the yield of 91%; m.p. 172.3–174.4 °C. ${\left[\alpha \right]}_{D}^{25}$ +15.6 (*c* 0.02, MeOH); UV (MeOH) λmax (log ε): 251 (3.01), 388 (3.31), 489 (1.66) nm; IR (KBr) *ν*_max_: 2968, 2921, 1743, 1708, 1610, 1491, 1369, 1288, 1245, 1180, 1089, 955 cm^−1^. ^1^H NMR (600 MHz, CDCl_3_) δ_H_ 7.90 (s, 1H), 7.07 (s, 1H), 6.98 (d, *J* = 15.6 Hz, 1H), 6.55 (d, *J* = 15.6 Hz, 1H), 5.72 (d, *J* = 9.8 Hz, 1H), 3.27 (dd, *J* = 6.8, 3.3 Hz, 1H), 2.68–2.39 (m, 1H), 2.16 (s, 3H), 1.86 (s, 3H), 1.53 (s, 3H), 1.48–1.40 (m, 1H), 1.37–1.31 (m, 1H), 1.22 (d, *J* = 6.8 Hz, 2H), 1.05 (dd, *J* = 15.1, 2.5 Hz, 2H), 1.02 (d, *J* = 6.7 Hz, 3H), 0.88 (t, *J* = 7.4 Hz, 3H). LRMS (EI) *m/z* 431 [M]^+^.

Compound **13**

(*R*)-5-chloro-3-((*S*, 1*E*, 3*E*)-3, 5-dimethylhepta-1, 3-dien-1-yl)-7-methyl-6, 8-dioxo-2-(thiophen-2-ylmethyl)-2, 6, 7, 8-tetrahydroisoquinolin-7-yl acetate. Red solid, the yield of 89%; m.p. 106.5–108.4 °C. ${\left[\alpha \right]}_{D}^{25}$ +13.3 (*c* 0.02, MeOH); UV (MeOH) λmax (log ε): 244 (3.00), 381 (3.10), 497 (1.68) nm; IR (KBr) *ν*_max_: 2960, 2924, 1735, 1704, 1591, 1499, 1371, 1256, 1140, 1080, 1008, 860 cm^−1^. ^1^H NMR (600 MHz, CDCl_3_) δ_H_ 7.86 (s, 1H), 7.35 (dd, *J* = 3.9, 2.4 Hz, 1H), 7.10–6.98 (m, 3H), 6.92 (d, *J* = 15.3 Hz, 1H), 6.22 (dd, *J* = 15.3 Hz, 1H), 5.68 (d, *J* = 9.8 Hz, 1H), 5.17 (q, *J* = 16.2 Hz, 2H), 2.54–2.41 (m, 1H), 2.15 (s, 3H), 1.80 (d, *J* = 10 Hz, 3H), 1.54 (s, 3H), 1.46–1.40 (m, 1H), 1.37–1.29 (m, 1H), 1.01 (d, *J* = 6.7 Hz, 3H), 0.87 (t, *J* = 7.4 Hz, 3H). ^13^C NMR (150 MHz, CDCl_3_) δ_C_ 193.8, 184.6, 170.3, 148.3, 148.0, 145.3, 144.4, 140.9, 136.3, 131.9, 127.8, 127.5, 127.2, 115.1, 114.9, 111.7, 103.0, 85.0, 52.6, 35.2, 30.1, 23.3, 20.4, 20.3, 12.7, 12.1. HRMS (ESI) for [M + H]^+^: calcd for C_26_H_29_ClNO_5_S: 486.14276. Found: 486.15086.

Compound **14**

(*R*)-5-chloro-3-((*S*, 1*E*, 3*E*)-3,5-dimethylhepta-1, 3-dien-1-yl)-7-methyl-6, 8-dioxo-2-(2-(thiophen-2-yl)ethyl)-2, 6, 7, 8-tetrahydroisoquinolin-7-yl acetate. Red solid, the yield of 91%; m.p. 100.2–102.0 °C. ${\left[\alpha \right]}_{D}^{25}$ +16.2 (*c* 0.02, MeOH); UV (MeOH) λmax (log ε): 251 (3.09), 389 (3.00), 501 (1.55) nm; IR (KBr) *ν*_max_: 2966, 2920, 1735, 1708, 1591, 1489, 1371, 1258, 1140, 1088, 1008, 849 cm^−1^. ^1^H NMR (600 MHz, CDCl_3_) δ_H_ 7.59 (s, 1H), 7.19 (dd, *J* = 5.1, 1.1 Hz, 1H), 6.97 (s, 1H), 6.95–6.88 (m, 2H), 6.78 (d, *J* = 3.3 Hz, 1H), 5.98 (d, *J* = 15.3 Hz, 1H), 5.68 (d, *J* = 9.7 Hz, 1H), 4.08 (ddt, *J* = 78.5, 14.6, 7.2 Hz, 2H), 3.34–3.13 (m, 2H), 2.52–2.39 (m, 1H), 2.16 (s, 3H), 1.82 (d, *J* = 1.0 Hz, 3H), 1.52 (s, 3H), 1.49–1.39 (m, 1H), 1.38–1.31 (m,1H), 1.02 (d, *J* = 6.7 Hz, 3H), 0.88 (t, *J* = 7.4 Hz, 3H). ^13^C NMR (150 MHz, CDCl_3_) δ_C_ 193.8, 184.4, 170.2, 148.3, 147.9, 145.2, 144.4, 140.9, 137.4, 131.8, 127.8, 126.9, 125.4, 114.8, 114.5, 111.6, 102.6, 85.0, 55.5, 35.2, 30.6, 30.2, 23.4, 20.4, 20.4, 12.7, 12.1. HRMS (ESI) for [M + H]^+^: calcd for C_27_H_31_ClNO_5_S: 500.15841. Found: 500.16563.

Compound **15**

(*R*)-5-chloro-3-((*S*, 1*E*, 3*E*)-3, 5-dimethylhepta-1, 3-dien-1-yl)-7-methyl-6, 8-dioxo-2-phenyl-2, 6, 7, 8-tetrahydroisoquinolin-7-yl acetate. Red solid, the yield of 93%; m.p. 190.2–192.1 °C. ${\left[\alpha \right]}_{D}^{25}$ +16.5 (*c* 0.02, MeOH); UV (MeOH) λmax (log ε): 241 (3.19), 379 (3.12), 506 (1.53) nm; IR (KBr) *ν*_max_: 3088, 2964, 2916, 1731, 1707, 1611, 1591, 1495, 1363, 1275, 1204, 1130, 1084, 1020, 856, 773 cm^−1^. ^1^H NMR (600 MHz, CDCl_3_) δ_H_ 7.83 (s, 3H), 7.59–7.47 (m, 3H), 7.32–7.27 (m, 2H), 7.15 (s, 1H), 6.96 (d, *J* = 15.6 Hz), 5.65 (d, *J* = 9.7 Hz, 1H), 5.58 (d, *J* = 15.6 Hz, 1H), 2.46–2.30 (m, 1H), 2.17 (s, 3H), 1.58 (s, 3H), 1.49 (d, *J* = 0.9 Hz, 3H), 1.45–1.36 (m, 1H), 1.33–1.28 (m, 1H), 0.97 (d, *J* = 6.6 Hz, 3H), 0.84 (t, 7.4 Hz, 3H). ^13^C NMR (150 MHz, CDCl_3_) δ_C_ 193.9, 184.8, 170.3, 148.0, 147.6, 144.2, 143.3, 141.3, 140.5, 131.9, 130.3, 130.3, 130.2, 126.7, 126.7, 116.3, 114.5, 109.9, 103.4, 84.9, 35.1, 30.1, 23.3, 20.4, 20.3, 12.3, 12.1. LRMS (EI) *m/z* 467 [M]^+^.

Compound **16**

(*R*)-5-chloro-3-((*S*, 1*E*, 3*E*)-3,5-dimethylhepta-1, 3-dien-1-yl)-7-methyl-6, 8-dioxo-2-phenethyl-2, 6, 7, 8-tetrahydroisoquinolin-7-yl acetate. Red solid, the yield of 91%; m.p. 180.2–181.0 °C. ${\left[\alpha \right]}_{D}^{25}$ +15.3 (*c* 0.02, MeOH); UV (MeOH) λmax (log ε): 243 (3.06), 386 (3.14), 498 (1.55) nm; IR (KBr) *ν*_max_: 3080, 2962, 2916, 1731, 1715, 1611, 1588, 1495, 1366, 1275, 1212, 1130, 1084, 1022, 856, 780 cm^−1^. ^1^H NMR (600 MHz, CDCl_3_) δ_H_ 7.51 (s, 1H), 7.30 (dd, *J* = 10.2, 4.6 Hz, 2H), 7.26–7.22 (m, 1H), 7.15- 7.01 (m, 2H), 6.94 (s, 1H), 6.88 (d, *J* = 15.2 Hz, 1H), 5.99 (d, *J* = 15.4 Hz, 1H), 5.67 (d, *J* = 9.7 Hz, 1H), 4.32–3.89 (m, 2H), 3.28–2.84 (m, 2H), 2.53–2.40 (m, 1H), 2.14 (s, 3H), 1.80 (d, *J* = 1.0 Hz, 3H), 1.50 (s, 3H), 1.48–1.40 (m, 1H), 1.38–1.31 (m, 1H), 1.02 (d, *J* = 6.7 Hz, 3H), 0.88 (t, *J* = 7.4 Hz, 3H). ^13^C NMR (150 MHz, CDCl_3_) δ_C_ 193.8, 184.4, 170.1, 148.1, 148.0, 145.1, 144.5, 141.1, 135.9, 131.8, 129.3, 129.3, 128.9, 128.9, 127.8, 114.7, 114.7, 111.5, 102.3, 85.0, 55.6, 36.7, 35.1, 30.1, 23.4, 20.4, 20.3, 12.7, 12.1. LRMS (EI) *m/z* 495 [M]^+^.

Compound **17**

(*R*)-2-benzyl-5-chloro-3-((*S*, 1*E*, 3*E*)-3,5-dimethylhepta-1, 3-dien-1-yl)-7-methyl-6, 8-dioxo-2, 6, 7, 8-tetrahydroisoquinolin-7-yl acetate. Red solid, the yield of 92%; m.p. 188.8–190.2 °C. ${\left[\alpha \right]}_{D}^{25}$ +13.9 (*c* 0.02, MeOH); UV (MeOH) λmax (log ε): 259 (3.06), 399 (3.10), 499 (1.65) nm; IR (KBr) *ν*_max_: 3082, 2964, 2918, 1731, 1710, 1611, 1591, 1495, 1363, 1276, 1204, 1130, 1084, 1020, 858, 773 cm^−1^. ^1^H NMR (600 MHz, CDCl_3_) δ_H_ 7.86 (s, 1H), 7.45–7.33 (m, 3H), 7.18 (d, *J* = 7.2 Hz, 2H), 7.04 (s, 1H), 6.89 (d, *J* = 15.4 Hz, 1H), 6.02 (d, *J* = 15.4 Hz, 1H), 5.64 (d, *J* = 9.8 Hz, 1H), 5.19–4.88 (m, 2H), 2.54–2.30 (m, 1H), 2.16 (s, 3H), 1.66 (d, *J* = 1.0 Hz, 3H), 1.57 (s, 3H), 1.46–1.40 (m, 1H), 1.34–1.28 (s, 1H), 0.98 (d, *J* = 6.7 Hz, 3H), 0.84 (t, *J* = 6.7 Hz, 3H). ^13^C NMR (150 MHz, CDCl_3_) δ_C_ 194.0, 184.6, 170.3, 148.4, 148.2, 145.0, 144.5, 141.7, 134.2, 131.8, 129.7, 129.7, 129.1, 126.6, 126.6, 115.2, 114.9, 111.7, 102.9, 84.9, 57.9, 35.1, 30.1, 23.4, 20.4, 20.3, 12.6, 12.1. HRMS (ESI) for [M + H]^+^: calcd for C_28_H_31_ClNO_4_: 480.1863. Found: 480.1935.

Compound **18**

(*R*)-5-chloro-3-((*S*, 1*E*, 3*E*)-3, 5-dimethylhepta-1, 3-dien-1-yl)-7-methyl-2-(3-morpholinopropyl) -6, 8-dioxo-2, 6, 7, 8-tetrahydroisoquinolin-7-yl acetate. Red solid, the yield of 96%; m.p. 110.2–112.6 °C. ${\left[\alpha \right]}_{D}^{25}$ +16.6 (*c* 0.02, MeOH); UV (MeOH) λmax (log ε): 245 (3.19), 376 (3.00), 505 (1.63) nm; IR (KBr) *ν*_max_: 2960, 2920, 1727, 1706, 1595, 1499, 1371, 1248, 1208, 1148, 1080, 864 cm^−1^. ^1^H NMR (600 MHz, CDCl_3_) δ_H_ 7.93 (s, 1H), 7.01 (s, 1H), 6.96 (d, *J* = 15.4 Hz, 1H), 6.18 (d, *J* = 15.4 Hz, 1H), 5.71 (d, *J* = 9.7 Hz, 1H), 4.01 (s, 2H), 3.75 (s, 4H), 2.68–2.24 (m, 7H), 2.16 (s, 3H), 1.98 (dd, *J* = 28.3, 21.6 Hz, 2H), 1.86 (s, 3H), 1.54 (s, 3H), 1.46–1.40 (m, 1H), 1.37–1.32 (m, 1H), 1.02 (d, *J* = 6.6 Hz, 3H), 0.88 (t, *J* = 7.4 Hz, 3H). ^13^C NMR (150 MHz, CDCl_3_) δ_C_ 194.1, 184.5, 170.2, 148.4, 147.9, 145.1, 144.6, 142.2, 131.7, 114.6, 114.4, 111.7, 102.3, 84.9, 54.0, 53.4, 51.5, 35.2, 30.1, 29.8, 23.4, 20.4, 20.3, 12.8, 12.1. 10.9, 10.8. HRMS (ESI) for [M + H]^+^: calcd for C_28_H_38_ClN_2_O_5_: 517.22345. Found:517.24647.

Compound **19**

(*R*)-5-chloro-2-(2-(dimethylamino)ethyl)-3-((*S*, 1*E*, 3*E*)-3,5-dimethylhepta-1, 3-dien-1-yl)-7-methyl-6, 8-dioxo-2, 6, 7, 8-tetrahydroisoquinolin-7-yl acetate. Red solid, the yield of 92%; m.p. 185.6–187.2 °C. ${\left[\alpha \right]}_{D}^{25}$ +14.8 (*c* 0.02, MeOH); UV (MeOH) λmax (log ε): 236 (3.01), 371 (3.23), 487 (1.45) nm; IR (KBr) *ν*_max_: 2960, 2923, 1731, 1706, 1595, 1499, 1371, 1251, 1209, 1140, 1080, 866 cm^−1^. ^1^H NMR (600 MHz, CDCl_3_) δ_H_ 7.77 (s, 1H), 7.00 (s, 1H), 6.94 (d, *J* = 15.3 Hz, 1H), 6.21 (d, *J* = 15.4 Hz, 1H), 5.70 (d, *J* = 9.70 Hz, 1H), 3.93 (s, 2H), 2.64 (s, 2H), 2.53–2.41 (m, 1H), 2.31 (s, 6H), 2.16 (s, 3H), 1.85 (d, *J* = 0.8 Hz, 3H), 1.54 (s, 3H), 1.46–1.40 (m, 1H), 1.39–1.31 (m, 1H), 1.02 (d, *J* = 6.6 Hz, 3H), 0.88 (t, *J* = 7.4 Hz, 3H). ^13^C NMR (150 MHz, CDCl_3_) δ_C_ 193.9, 184.4, 170.1, 148.1, 148.0, 145.1, 144.5, 141.5, 131.7, 114.8, 114.6, 111.6, 102.3, 84.9, 58.5, 51.8, 45.6, 35.1, 30.0, 29.3, 23.3, 20.3, 20.2, 12.6, 12.0. HRMS (ESI) for [M + H]^+^: calcd for C_25_H_34_ClN_2_O_4_: 461.26289. Found: 461.22027.

Compound **20**

(*R*)-2-(2-(benzylamino)ethyl)-5-chloro-3-((*S*, 1*E*, 3*E*)-3, 5-dimethylhepta-1, 3-dien-1-yl)-7-methyl-6, 8-dioxo-2, 6, 7, 8-tetrahydroisoquinolin-7-yl acetate. Red solid, the yield of 91%; m.p. 160.1–161.8. ${\left[\alpha \right]}_{D}^{25}$ +14.3 (*c* 0.02, MeOH); UV (MeOH) λmax (log ε): 251 (3.03), 381 (3.26), 497 (1.55) nm; IR (KBr) *ν*_max_: 3412, 2963, 2924, 1737, 1703, 1589, 1495, 1369, 1249, 1145, 1082, 1007, 965, 861 cm^−1^. ^1^H NMR (600 MHz, CDCl_3_) δ_H_ 7.81 (s, 1H), 7.32–7.27 (m, 2H), 7.24 (td, *J* = 7.6, 2.1 Hz, 3H), 6.94 (s, 1H), 6.83 (d, *J* = 15.3 Hz, 1H), 6.16 (d, *J* = 15.4 Hz, 1H), 5.66 (d, *J* = 9.7 Hz, 1H), 4.09–3.80 (m, 2H), 3.79 (s, 2H), 3.12–2.77 (m, 2H), 2.53–2.37 (m, 1H), 2.16 (s, 3H), 1.80 (d, *J* = 1.0 Hz, 3H), 1.55 (s, 3H), 1.49–1.41 (m, 1H), 1.37–1.32 (m, 1H), 1.01 (d, *J* = 6.7 Hz, 3H), 0.88 (t, *J* = 7.4 Hz, 3H). ^13^C NMR (150 MHz, CDCl_3_) δ_C_ 194.1, 184.5, 170.2, 148.1, 148.0, 145.1, 144.8, 142.1, 131.8, 128.8, 128.8, 128.8, 128.3, 128.3, 127.6, 114.9, 114.4, 111.8, 102.2, 85.0, 54.1, 53.6, 47.5, 35.13, 30.2, 23.4, 20.5, 20.4, 12.7, 12.1. HRMS (ESI) for [M + H]^+^: calcd for C_30_H_36_ClN_2_O_5_: 523.22854. Found: 523.23594.

Compound **21**

(*R*)-5-chloro-2-cyclopentyl-3-((*S*, 1*E*, 3*E*)-3, 5-dimethylhepta-1, 3-dien-1-yl)-7-methyl-6, 8-dioxo-2, 6, 7, 8-tetrahydroisoquinolin-7-yl acetate. Red solid, the yield of 95%; m.p. 132.3–133.5 °C. ${\left[\alpha \right]}_{D}^{25}$ +15.3 (*c* 0.02, MeOH); UV (MeOH) λmax (log ε): 233 (3.09), 369 (3.13), 499 (1.65) nm; IR (KBr) *ν*_max_: 2971, 2925, 1743, 1700, 1600, 1491, 1368, 1288, 1246, 1180, 1089, 955 cm^−1^. ^1^H NMR (600 MHz, CDCl_3_) δ_H_ 7.88 (s, 1H), 6.95 (s, 1H), 6.87 (d, *J* = 15.3 Hz, 1H), 6.19 (d, *J* = 15.3 Hz, 1H), 5.67 (d, *J* = 9.7 Hz, 1H), 4.53 (p, *J* = 7.4 Hz, 1H), 2.55–2.38 (m, 1H), 2.29–2.12 (m, 5H), 1.91 (ddd, *J* = 16.4, 8.5, 5.0 Hz, 2H), 1.85 (d, *J* = 1.0 Hz, 3H), 1.84–1.70 (m, 4H), 1.54 (s, 3H), 1.46–1.39 (m,1H), 1.38–1.30 (m, 1H), 1.01 (d, *J* = 6.7 Hz, 3H), 0.88 (t, *J* = 7.4 Hz, 3H). ^13^C NMR (150 MHz, CDCl_3_) δ_C_ 194.1, 184.4, 170.2, 149.1, 147.7, 144.9, 144.5, 136.9, 131.7, 115.6, 115.3, 112.7, 102.2, 84.9, 62.7, 35.1, 33.3, 33.2, 30.1, 24.3, 24.3, 23.4, 20.4, 20.4, 12.8, 12.1. HRMS (ESI) for [M + H]^+^: calcd for C_26_H_33_ClNO_4_: 458.20199. Found: 458.20981.

Compound **22**

(*R*)-5-chloro-2-cyclohexyl-3-((*S*, 1*E*, 3*E*)-3, 5-dimethylhepta-1, 3-dien-1-yl)-7-methyl-6, 8-dioxo-2, 6, 7, 8-tetrahydroisoquinolin-7-yl acetate. Red solid, the yield of 95%; m.p. 136.2–138.1 °C. ${\left[\alpha \right]}_{D}^{25}$ +16.5 (*c* 0.02, MeOH); UV (MeOH) λmax (log ε): 248 (3.08), 379 (3.16), 503 (1.51) nm; IR (KBr) *ν*_max_: 2955, 2918, 1743, 1705, 1600, 1491, 1368, 1286, 1248, 1180, 1089, 923 cm^−1^. ^1^H NMR (600 MHz, CDCl_3_) δ_H_ 7.90 (s, 1H), 6.96 (s, 1H), 6.88 (d, *J* = 15.2 Hz, 1H), 6.14 (d, *J* = 15.3 Hz, 1H), 5.68 (d, *J* = 9.7 Hz, 1H), 3.97 (tt, *J* = 12.0, 3.2 Hz, 1H), 2.65–2.34 (m, 1H), 2.16 (s, 3H), 1.98 (dd, *J* = 23.3, 12.1 Hz, 5H), 1.85 (d, *J* = 1.0 Hz, 3H), 1.77 (d, *J* = 13.3 Hz, 1H), 1.64 (tt, 12.4, 6.3 Hz, 2H), 1.47–1.31 (m, 4H), 1.29–1.16 (m, 3H), 1.02 (d, *J* = 6.7 Hz, 3H), 0.88 (t, *J* = 7.4 Hz, 3H). ^13^C NMR (150 MHz, CDCl_3_) δ_C_ 194.1, 184.4, 170.2, 148.6, 147.7, 145.1, 144.6, 136.9, 131.6, 115.1, 115.0, 112.9, 102.1, 84.9, 61.2, 35.1, 33.3, 33.1, 30.1, 25.8, 25.8, 25.0, 23.4, 20.4, 20.4, 12.7, 12.1. LRMS (EI) *m/z* 472 [M]^+^.

Compound **23**

(*R*)-2-(4-bromophenyl)-5-chloro-3-((*S*, 1*E*, 3*E*)-3, 5-dimethylhepta-1, 3-dien-1-yl)-7-methyl-6, 8-dioxo-2, 6, 7, 8-tetrahydroisoquinolin-7-yl acetate. Red solid, the yield of 91%; m.p. 190.0–192.3 °C. ${\left[\alpha \right]}_{D}^{25}$ +16.8 (*c* 0.02, MeOH); UV (MeOH) λmax (log ε): 236 (3.03), 377 (3.25), 493 (1.48) nm; IR (KBr) *ν*_max_: 3101, 2959, 2919, 1735, 1707, 1601, 1508, 1487, 1365, 1268, 1250, 1207, 1129, 1080, 960 cm^−1^. ^1^H NMR (600 MHz, CDCl_3_) δ_H_ 7.76 (s, 1H), 7.73–7.63 (m, 2H), 7.24–7.15 (m, 2H), 7.12 (s, 1H), 6.96 (d, *J* = 15.5 Hz, 1H), 5.67 (d, *J* = 9.7 Hz, 1H), 5.55 (d, *J* = 15.5 Hz, 1H), 2.53–2.32 (m, 1H), 2.17 (s, 3H), 1.63–1.49 (m, 6H), 1.46–1.40 (m, 1H), 1.39–1.31 (m, 1H), 0.99 (d, *J* = 6.6 Hz, 3H), 0.85 (t, *J* = 7.4 Hz, 3H). ^13^C NMR (150 MHz, CDCl_3_) δ_C_ 193.7, 184.9, 170.3, 148.3, 147.2, 143.8, 143.6, 140.9, 139.5, 133.6, 133.6, 131.9, 128.3, 128.3, 124.3, 116.0, 114.7, 110.1, 104.0, 84.8, 35.1, 30.1, 23.2, 20.4, 20.3, 12.5, 12.1. HRMS (ESI) for [M + H]^+^: calcd for C_27_H_28_ClBrNO_4_: 544.08120. Found: 544.08855.

Compound **24**

(*R*)-5-chloro-3-((*S*, 1*E*, 3*E*)-3, 5-dimethylhepta-1, 3-dien-1-yl)-2-ethyl-7-methyl-6, 8-dioxo-2, 6, 7, 8-tetrahydroisoquinolin-7-yl acetate. Red solid, the yield of 91%; m.p. 250.0–251.1 °C. ${\left[\alpha \right]}_{D}^{25}$ +15.5 (*c* 0.02, MeOH); UV (MeOH) λmax (log ε): 238 (3.12), 385 (3.33), 499 (1.47) nm; IR (KBr) *ν*_max_: 2955, 2922, 1748, 1712, 1591, 1491, 1368, 1252, 1190, 1093, 853 cm^−1^. ^1^H NMR (600 MHz, CDCl_3_) δ_H_ 7.78 (s, 1H), 6.99 (s, 1H), 6.95 (d, *J* = 15.3 Hz, 1H), 6.14 (d, *J* = 15.4 Hz, 1H), 5.69 (d, *J* = 9.7 Hz, 1H), 3.92 (q, *J* = 7.3 Hz, 2H), 2.52–2.36 (m, 1H), 2.14 (s, 3H), 1.84 (d, *J* = 0.9 Hz, 3H), 1.52 (s, 3H), 1.46–1.36 (m, 4H), 1.36–1.27 (m, 1H), 1.00 (d, *J* = 6.7 Hz, 3H), 0.86 (t, *J* = 7.4 Hz, 3H). ^13^C NMR (150 MHz, CDCl_3_) δ_C_ 193.9, 184.3, 170.2, 148.2, 147.9, 145.1, 144.8, 140.8, 131.7, 115.0, 114.5, 111.7, 102.0, 84.9, 49.5, 35.1, 30.1, 23.2, 20.4, 20.3, 15.7, 12.7, 12.1. HRMS (ESI) for [M + H]^+^: calcd for C_23_H_29_ClNO_4_: 418.17069. Found: 418.17845.

#### 3.1.5. General Procedure for Synthesis of 25

To a solution of sclerotiorin (39 mg, 0.10 mmol) in anhydrous dichloromethane (2 mL), the CH_3_ONa (54 mg, 10 mmol) was add. The resulting reaction mixture was stirred at 40 °C until the sclerotiorin was disappeared. 12 mL HCl (2M) was added to the mixture to regulate the pH to 2. The mixture was extracted with EtOAc (3 × 5 mL) and the organic phase was washed with saturated brine (3 × 15 mL). Then the organic phase was dried with MgSO_4_ and concentrated in vacuo. The crude product was purified by column chromatography with eluent of EA/PE (2:1, *v*/*v*) to give the pure compound.

Compound **25**

(*R*)-5-chloro-3-((*S*, 1*E*, 3*E*)-3, 5-dimethylhepta-1, 3-dien-1-yl)-7-hydroxy-7-methyl-6H-isochromene-6, 8 (7H)-dione. Yellow solid, the yield of 88%; m.p. 130.5–132.1 °C. ${\left[\alpha \right]}_{D}^{25}$ +221.7 (*c* 0.6, MeOH); UV (MeOH) λmax (log ε): 234 (3.01), 344 (3.13) nm; IR (KBr) *ν*_max_: 3537, 2949, 1625, 1510, 1183, 1154, 1131, 1079, 966 cm^−1^. ^1^H NMR (600 MHz, CD_3_OD) δ_H_ 8.13 (s,1H), 7.17 (d, *J* = 15.7 Hz, 1H), 6.81 (s, 1H), 6.36 (d, *J* = 15.7 Hz, 1H), 5.74 (d, *J* = 9.8 Hz, 1H), 2.63–2.42 (m, 1H), 1.90 (d, *J* = 1.1 Hz, 3H), 1.52 (s, 3H), 1.49–1.46 (m, 1H), 1.38–1.34 (m, 1H), 1.00 (d, *J* = 6.7 Hz, 3H), 0.86 (t, *J* = 7.4 Hz, 3H). ^13^C NMR (150 MHz, CD_3_OD) δ_C_ 196.8, 191.8, 160.2, 153.6, 149.2, 143.8, 141.4, 133.8, 117.5, 116.4, 110.4, 107.0, 84.9, 36.3, 31.2, 27.7, 20.6, 12.5, 12.3. HRMS (ESI) for [M + H]^+^: calcd for C_19_H_22_ClO_4_: 349.1128. Found: 349.1212.

#### 3.1.6. General Procedure for Synthesis of 26 and 27

A solution of **25** (39 mg, 0.11 mmol) and anhydride (1.10 mmol) in anhydrous pyridine (3 mL) was refluxed at 90 °C until the **25** disappeared. After being cooled to room temperature, 2 mL HCl (2M) was added to the mixture to regulate the pH to 2. The resulting mixture was extracted with EtOAc (3 × 5 mL) and the organic phase was washed with saturated brine (3 × 15 mL). Then the organic phase was dried with MgSO_4_ and concentrated in vacuo. The crude product was purified by column chromatography with eluent of EA/PE (1:10, *v*/*v*) to give the pure compound.

Compound **26**

(*R*)-5-chloro-3-((*S*, 1*E*, 3*E*)-3, 5-dimethylhepta-1, 3-dien-1-yl)-7-methyl-6, 8-dioxo-7, 8-dihydro-6H-isochromen-7-yl butyrate. Yellow oil, the yield of 78%. ${\left[\alpha \right]}_{D}^{25}$ +42.8 (*c* 0.2, MeOH); UV (MeOH) λmax (log ε): 241 (3.08), 348 (3.15) nm; IR (KBr) *ν*_max_: 2949, 1737, 1625, 1509, 1180, 1135, 1079, 958 cm^−1^. ^1^H NMR (600 MHz, CD_3_OD) δ_H_ 8.19 (s, 1H), 7.19 (d, *J* = 15.7 Hz, 1H), 6.87 (s, 1H), 6.38 (d, *J* = 15.7 Hz, 1H), 5.75 (d, *J* = 9.8 Hz, 1H), 2.63–2.51 (m, 1H), 2.43 (t, *J* = 7.4 Hz, 2H), 1.90 (d, *J* = 1.1 Hz, 3H), 1.64 (ddd, *J* = 7.1, 5.0, 2.5 Hz, 2H), 1.55 (s, 3H), 1.51–1.44 (m, 1H), 1.38–1.34 (m, 5H), 1.04 (d, *J* = 6.7 Hz, 3H), 0.94 (t, *J* = 7.1HZ, 3H), 0.90 (t, *J* = 7.4 Hz, 3H). ^13^C NMR (150 MHz, CD_3_OD) δ_C_ 192.9, 188.1, 174.2, 160.5, 155.1, 149.4, 144.1, 141.5, 133.9, 117.4, 115.8, 110.7, 107.3, 86.0, 36.3, 34.0, 32.2, 31.2, 25.5, 23.4, 22.9, 20.6, 14.2, 12.5, 12.4. HRMS (ESI) for [M + H]^+^: calcd for C_22_H_32_ClO_5_: 447.1600. Found: 447.1936.

Compound **27**

(*R*)-5-chloro-3-((*S*, 1*E*, 3*E*)-3, 5-dimethylhepta-1, 3-dien-1-yl)-7-methyl-6, 8-dioxo-7, 8-dihydro-6H-isochromen-7-yl propionate. yellow oil, the yield of 83%. ${\left[\alpha \right]}_{D}^{25}$ +37.2 (*c* 0.2, MeOH); UV (MeOH) λmax (log ε): 238 (3.11), 340 (3.08) nm; IR (KBr) *ν*_max_: 2959, 1738, 1625, 1506, 1180, 1135, 1079, 962 cm^−1^. ^1^H NMR (600 MHz, CD_3_OD) δ_H_ 8.20 (s, 1H), 7.19 (d, *J* = 15.7 Hz, 1H), 6.88 (s, 1H), 6.38 (d, *J* = 15.7 Hz, 1H), 5.76 (d, *J* = 9.8 Hz, 1H), 2.59–2.51 (m, 1H), 2.49–2.45 (m, 2H), 1.90 (d, *J* = 1.0 Hz, 3H), 1.56 (s, 3H), 1.51–1.44 (m, 1H), 1.39–1.34 (m, 1H), 1.14 (t, *J* = 7.6 Hz, 3H), 1.04 (d, *J* = 6.6 Hz, 3H), 0.90 (t, *J* = 7.4 Hz, 3H). ^13^C NMR (150 MHz, CD_3_OD) δ_C_ 192.9, 188.1, 174.9, 160.5, 155.1, 149.5, 144.1, 141.5, 133.9, 117.4, 115.8, 110.7, 107.3, 86.1, 36.3, 31.2, 27.5, 22.9, 20.6, 12.5, 12.3, 9.1. HRMS (ESI) for [M + H]^+^: calcd for C_22_H_26_ClO_5_: 405.1391. Found: 405.1465.

### 3.2. Cytotoxic Activity 

The test compounds at a serial final concentration of 50, 25, 12.5, 6.25, and 3.125 µM were evaluated against A549 (human lung cancer cells), MDA-MB-435 (breast cancer cells) using the MTT method. Human breast cancer cell lines MDA-MB-435, human lung cancer cell line A549 were obtained from Keygen Biotech (Nanjing, China) and cultuRed in Dulbecco’s modified Eagle’s medium (DMEM) (Invitrogen, Carlsbad, CA, USA) supplemented with 5% fetal bovine serum (Hyclone, Logan, UT, USA), 2 mM l-glutamine, 100 mg/mL streptomycin, and 100 units/mL penicillin (Invitrogen). The cultures were maintained at 37 °C in a humidified atmosphere of 5% CO_2_.

### 3.3. COX-2 Inhibitory Activity

The in vitro inhibitory of the compounds were evaluated by using COX-2 (human) inhibitor screening assay kit (Item NO:701080) supplied by Cayman chemicals USA. Indomethacin was used as a positive control and the tested compounds was dissolved in DMSO.

Each compound was tested in triplicate at 20 μM, and the percent inhibition for COX-2 was obtained for each experiment. The amount of prostaglandins produced by enzyme in the presence of the test compounds was measured and compared with the control experiments (also performed in triplicate) with enzyme inhibition = 1/concentration of prostaglandin in each enzymatic reaction (The percent inhibition of test compound was inversely proportional to the amount of prostaglandins produced by each wells). Finally, the calculations were performed as per the kit guidelines.

### 3.4. Molecular Docking

The X-ray crystal structure of COX-2 cyclooxygenase (PDB ID: 5GMN) enzyme was obtained from protein data bank in PDB format as initialing point. The crystal original ligand was extracted from the crystal structure prior to docking. Then, all waters were removed in the crystal structure. Hydrogens addition and Gasteiger charges were executed in turn. The protein was regarded as rigid and the conformation of the ligand was regarded as changeable. The parameter of grid box was set as 100 × 100 × 100 points and center on the protein. The Lamarckian genetic algorithm was used to dock algorithm and number of GA runs was 100. PyMOL [38] and LigPolt [39] were used to visualize and analyze results.

## 4. Conclusions

In summary, a novel series of sclerotiorin derivatives were synthesized. All compounds have been screened for their cytotoxic activity in vitro. Most of sclerotiorin derivatives showed good to great cytotoxic activity. In addition, the COX-2 inhibitory results disclosed that most of the derivatives displayed considerable inhibition of COX-2. Particularly, compound **3** displayed superior inhibitory ratio of 70.6%, a comparable inhibition ratio to positive indomethacin (78.9%) in 20 μM. Moreover, both in vitro and in silico studies showed that some of the new compounds act as promising COX-2 inhibitors. The results of this research will provide useful information for the design of novel series of anticancer agents with COX-2 inhibitory activity.

## Figures and Tables

**Table 1:** Cytotoxic activity of sclerotiorin derivatives.

Compd.	MDA-MB-435 IC_50_ (μM)	A549 IC_50_ (μM)	Compd.	MDA-MB-435 IC_50_ (μM)	A549 IC_50_ (μM)
1	>50	40.29	2	>50	>50
3	6.39	22.75	4	35.49	>50
5	20.93	20.27	6	44.27	>50
7	9.20	21.47	8	23.52	21.16
9	38.74	40.35	10	11.76	20.19
11	11.13	25.46	12	9.76	25.98
13	7.75	14.08	14	15.66	21.07
15	9.08	22.70	16	21.29	15.06
17	8.18	14.15	18	26.21	32.05
19	16.34	25.29	20	15.71	23.35
21	22.42	28.18	22	16.26	16.53
23	10.23	23.75	24	42.92	21.46
25	>50	>50	26	>50	>50
27	>50	>50	Sclerotiorin	>50	>50
EPI	0.15	0.37			

## Data Availability

The data presented in this study are available in the Supplementary Materials.

## Supplementary Materials

The following are available online at https://www.mdpi.com/1660-3397/19/1/12/s1. Figure S1. MS spectrum of compound 1; Figures S2 and S3: NMR (in CDCl3) spectra of compound 1; Figure S4: MS spectrum of compound 2; Figures S5 and S6: NMR (in CDCl3) spectra of compound 2; Figure S7: HRMS spectrum of compound 3; Figures S8 and S9: NMR (in CDCl3) spectra of compound 3; Figure S10. HRMS spectrum of compound 4; Figures S11 and S12: NMR (in CD3OD) spectra of compound 4; Figure S13: HRMS spectrum of compound 5; Figures S14 and S15: NMR (in CDCl3) spectra of compound 5; Figure S16: HRMS spectrum of compound 6; Figures S17 and S18: NMR (in CDCl3) spectra of compound 6; Figure S19: HRMS spectrum of compound 7; Figures S20 and S21: NMR (in CDCl3) spectra of compound 7; Figure S22: HRMS spectrum of compound 8; Figures S23 and S24: NMR (in CDCl3) spectra compound 8; Figure S25: MS spectrum of compound 9; Figures S26 and S27: NMR (in CDCl3) spectrum of compound 9; Figure S27: HRMS spectrum of compound 10; Figures S28 and S29: NMR (in CDCl3) spectra of compound 10; Figure S30: HRMS spectrum of compound 11; Figures S31 and S32: NMR (in CDCl3) spectra of compound 11; Figure S33: MS spectrum of compound 12; Figure S34: ^1^H NMR (in CDCl3) spectra of compound 12; Figure S35: HRMS spectrum of compound 13; Figures S36 and S37: NMR (in CDCl3) spectra of compound 13; Figure S38: HRMS spectrum of compound 14; Figures S39 and S40: NMR (in CDCl3) spectra of compound 14; Figure S41: MS spectrum of compound 15; Figures S42 and S43: NMR (in CDCl3) spectra of compound 15; Figure S44: MS spectrum of compound 16; Figures S45 and S46: NMR (in CDCl3) spectra of compound 16; Figure S47: HRMS spectrum of compound 17; Figures S48 and S49: NMR (in CDCl3) spectra of compound 17; Figure S50: HRMS spectrum of compound 18; Figures S51 and S52: NMR (in CDCl3) spectra of compound 18; Figure S53: HRMS spectrum of compound 19; Figures S54 and S55: NMR (in CDCl3) spectra of compound 19; Figure S56: HRMS spectrum of compound 20; Figures S57 and S58: NMR (in CDCl3) spectra of compound 20; Figure S59: HRMS spectrum of compound 21; Figures S60 and S61: NMR (in CDCl3) spectra of compound 21; Figure S62: MS spectrum of compound 22; Figures S63 and S64: NMR (in CDCl3) spectra of compound 22; Figure S65: HRMS spectrum of compound 23; Figures S66 and S67: NMR (in CDCl3) spectra of compound 23; Figure S68c: HRMS spectrum of compound 24; Figures S69 and S70: NMR (in CDCl3) spectra of compound 24; Figure S71: HRMS spectrum of compound 25; Figures S72 and S73: NMR (in CDCl3) spectra of compound 25; Figure S74: HRMS spectrum of compound 26; Figures S75 and S76: NMR (in CDCl3) spectra of compound 26; Figure S77: HRMS spectrum of compound 27; Figures S78 and S79: NMR (in CDCl3) spectra of compound 27.
