# Supplementary material for: Preparation, COX-2 Inhibition and Anticancer Activity of Sclerotiorin Derivatives
Source: Mar Drugs. 2020 Dec 29;19(1):12. doi: 10.3390/md19010012 (PMC7823724; doi:10.3390/md19010012)
Supplement: Supplementary file 1 [file marinedrugs-19-00012-s001.pdf]

Supporting Information

Preparation, COX-2 Inhibition and Potent Anticancer Activity of Sclerotiorin Derivatives

Tao Chen[1], Yun Huang[2, 3], Junxian Hong[1], Xikang Wei[1], Fang Zeng[1], Jialin Li[1], Geting Ye[1], Jie Yuan[2,*], Yuhua Long[1,*]

[1] School of Chemistry, Guangzhou Key Laboratory of Analytical Chemistry for Biomedicine, South China Normal University, Guangzhou 510006, PR China; 1660564371@qq.com (T.C.); 1209256295@qq.com (J.H.); 2855836968@qq.com (X.W.); 874674100@qq.com (F.Z.); 605394658@qq.com (J.L.); 2779232198@qq.com (G.Y.); longyh@scnu.edu.cn (Y.L.)

[2] Department of Biochemistry, Zhongshan School of Medicine, Sun Yat-sen University, Guangzhou 510080, China; yuanjie@mail.sysu.edu.cn (J.Y.)

[3] School of Basic Medical Sciences, Southern Medical University, Guangzhou 510515, China; wyhl2014@163.com (Y.H.)

* Correspondence: longyh@scnu.edu.cn (Y.L.); yuanjie@mail.sysu.edu.cn (J. Y.)

CONTENT

Fig.S1. MS spectrum of compound 1 1

Fig.S2. ^{1}H NMR (600 MHz, $CDCl_3$) spectrum of compound 1 1

Fig.S3.^{13}C NMR (150 MHz, $CDCl_3$) spectrum of compound 1 2

Fig.S4. MS spectrum of compound 2 2

Fig.S5. ^{1}H NMR (600 MHz, $CDCl_3$) spectrum of compound 2 3

Fig.S6.^{13}C NMR (150 MHz, $CDCl_3$) spectrum of compound 2 3

Fig.S7. HRMS spectrum of compound 3 4

Fig.S8. ^{1}H NMR (600 MHz, $CDCl_3$) spectrum of compound 3 4

Fig.S9.^{13}C NMR (150 MHz, $CDCl_3$) spectrum of compound 3 5

Fig.S10. HRMS spectrum of compound 4 5

Fig.S11. ^{1}H NMR (600 MHz, CD_3OD) spectrum of compound 4 6

Fig.S12.^{13}C NMR (150 MHz, CD_3OD) spectrum of compound 4 6

Fig.S13. HRMS spectrum of compound 5 7

Fig.S14. ^{1}H NMR (600 MHz, $CDCl_3$) spectrum of compound 5 7

Fig.S15. ^{13}C NMR (150 MHz, $CDCl_3$) spectrum of compound 5 8

Fig.S16. HRMS spectrum of compound 6 8

Fig.S17. ^{1}H NMR (600 MHz, $CDCl_3$) spectrum of compound 6 9

Fig.S18. ^{13}C NMR (150 MHz, $CDCl_3$) spectrum of compound 6 9

Fig.S19. HRMS spectrum of compound 7 10

Fig.S20. ^{1}H NMR (600 MHz, $CDCl_3$) spectrum of compound 7 10

Fig.S21. ^{13}C NMR (150 MHz, $CDCl_3$) spectrum of compound 7 11

Fig.S22. HRMS spectrum of compound 8 11

Fig.S23. ^{1}H NMR (600 MHz, $CDCl_3$) spectrum of compound 8 12

Fig.S24. ^{13}C NMR (150 MHz, $CDCl_3$) spectrum of compound 8 12

Fig.S25. MS spectrum of compound 9 13

Fig.S26. ^{1}H NMR (600 MHz, $CDCl_3$) spectrum of compound 9 13

Fig.S27. HRMS spectrum of compound 10 14

Fig.S28. ^{1}H NMR (600 MHz, $CDCl_3$) spectrum of compound 10 14

Fig.S29. ^{13}C NMR (150 MHz, $CDCl_3$) spectrum of compound 10 15

Fig.S30. HRMS spectrum of compound 11 15

Fig.S31. ^{1}H NMR (600 MHz, $CDCl_3$) spectrum of compound 11 16

Fig.S32. ^{13}C NMR (150 MHz, $CDCl_3$) spectrum of compound 11 16

Fig.S33. MS spectrum of compound 12 17

Fig.S34. ^{1}H NMR (600 MHz, $CDCl_3$) spectrum of compound 12 17
Fig.S35. HRMS spectrum of compound 13 18
Fig.S36. ^{1}H NMR (600 MHz, $CDCl_3$) spectrum of compound 13 18
Fig.S37. ^{13}C NMR (150 MHz, $CDCl_3$) spectrum of compound 13 19
Fig.S38. HRMS spectrum of compound 14 19
Fig.S39. ^{1}H NMR (600 MHz, $CDCl_3$) spectrum of compound 14 20
Fig.S40. ^{13}C NMR (150 MHz, $CDCl_3$) spectrum of compound 14 20
Fig.S41. MS spectrum of compound 15........ 21
Fig.S42. ^{1}H NMR (600 MHz, $CDCl_3$) spectrum of compound 15 21
Fig.S43. ^{13}C NMR (150 MHz, $CDCl_3$) spectrum of compound 15 22
Fig.S44. MS spectrum of compound 16........ 22
Fig.S45. ^{1}H NMR (600 MHz, $CDCl_3$) spectrum of compound 16 23
Fig.S46. ^{13}C NMR (150 MHz, $CDCl_3$) spectrum of compound 16 23
Fig.S47. HRMS spectrum of compound 17 24
Fig.S48. ^{1}H NMR (600 MHz, $CDCl_3$) spectrum of compound 17 24
Fig.S49. ^{13}C NMR (150 MHz, $CDCl_3$) spectrum of compound 17 25
Fig.S50. HRMS spectrum of compound 18 25
Fig.S51. ^{1}H NMR (600 MHz, $CDCl_3$) spectrum of compound 18 26
Fig.S52. ^{13}C NMR (150 MHz, $CDCl_3$) spectrum of compound 18 26
Fig.S53. HRMS spectrum of compound 19 27
Fig.S54. ^{1}H NMR (600 MHz, $CDCl_3$) spectrum of compound 19 27
Fig.S55. ^{13}C NMR (150 MHz, $CDCl_3$) spectrum of compound 19 28
Fig.S56. HRMS spectrum of compound 20 28
Fig.S57. ^{1}H NMR (600 MHz, $CDCl_3$) spectrum of compound 20 29
Fig.S58. ^{13}C NMR (150 MHz, $CDCl_3$) spectrum of compound 20 29
Fig.S59. HRMS spectrum of compound 21 30
Fig.S60. ^{1}H NMR (600 MHz, $CDCl_3$) spectrum of compound 21 30
Fig.S61. ^{13}C NMR (150 MHz, $CDCl_3$) spectrum of compound 21 31
Fig.S62. MS spectrum of compound 22........ 31
Fig.S63. ^{1}H NMR (600 MHz, $CDCl_3$) spectrum of compound 22 32
Fig.S64. ^{13}C NMR (150 MHz, $CDCl_3$) spectrum of compound 22 32
Fig.S65. HRMS spectrum of compound 23 33
Fig.S66. ^{1}H NMR (600 MHz, $CDCl_3$) spectrum of compound 23 33
Fig.S67. ^{13}C NMR (150 MHz, $CDCl_3$) spectrum of compound 23 34

Fig.S68. HRMS spectrum of compound 24 34
Fig.S69. ^{1}H NMR (600 MHz, $CDCl_3$) spectrum of compound 24 35
Fig.S70. ^{13}C NMR (150 MHz, $CDCl_3$) spectrum of compound 24 35
Fig.S71. HRMS spectrum of compound 25 36
Fig.S72. ^{1}H NMR (600 MHz, $CDCl_3$) spectrum of compound 25 36
Fig.S73. ^{13}C NMR (150 MHz, $CDCl_3$) spectrum of compound 25 37
Fig.S74. HRMS spectrum of compound 26 38
Fig.S75. ^{1}H NMR (600 MHz, $CDCl_3$) spectrum of compound 26 38
Fig.S76. ^{13}C NMR (150 MHz, $CDCl_3$) spectrum of compound 26 38
Fig.S77. HRMS spectrum of compound 27 39
Fig.S78. ^{1}H NMR (600 MHz, $CDCl_3$) spectrum of compound 27 40
Fig.S79. ^{13}C NMR (150 MHz, $CDCl_3$) spectrum of compound 27 40

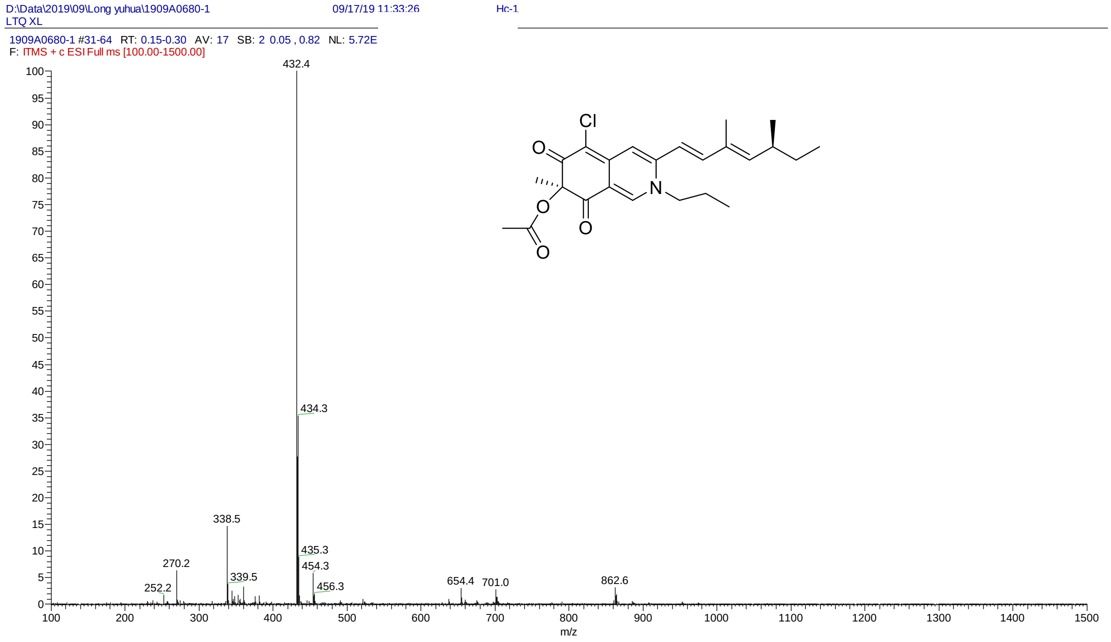

Fig.S1. MS spectrum of compound **1**

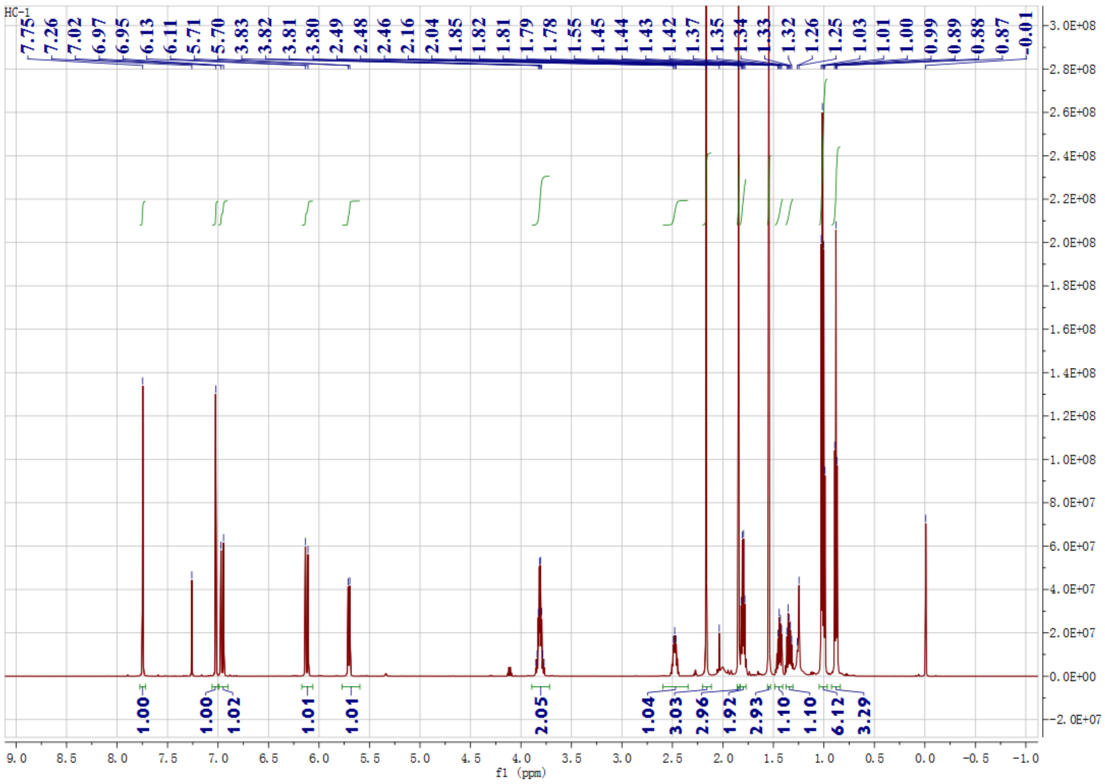

Fig.S2. ^{1}H NMR (600 MHz, $CDCl_3$) spectrum of compound **1**

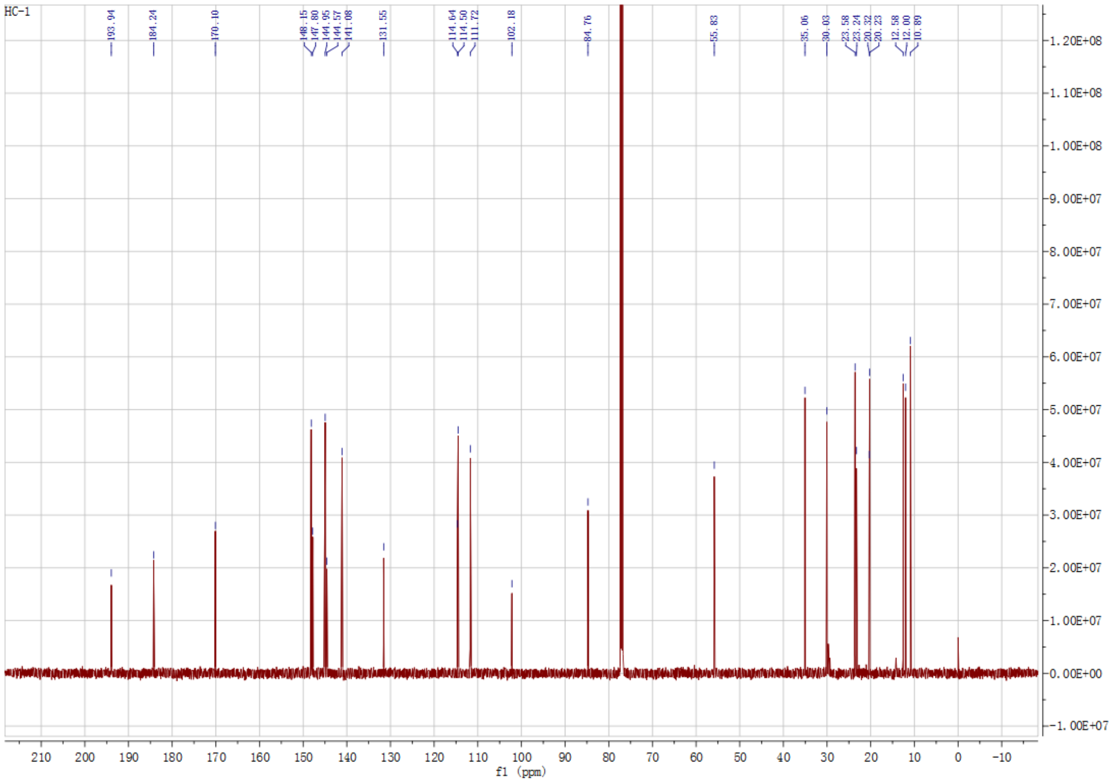

Fig.S3.^{13}C NMR (150 MHz, $CDCl_3$) spectrum of compound **1**

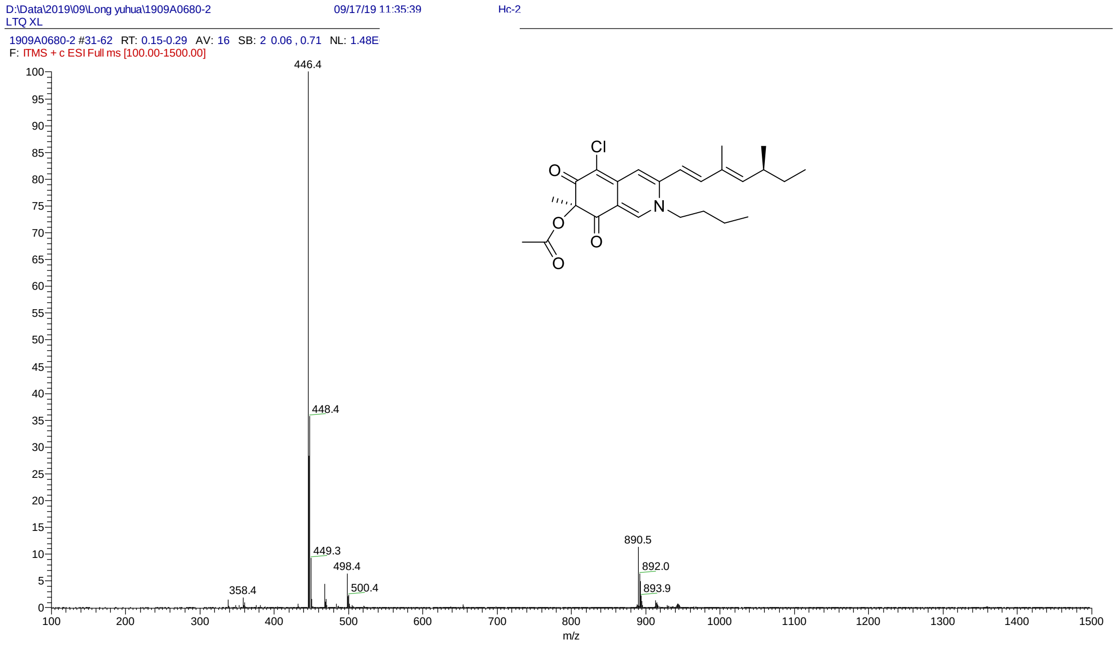

Fig.S4. MS spectrum of compound **2**

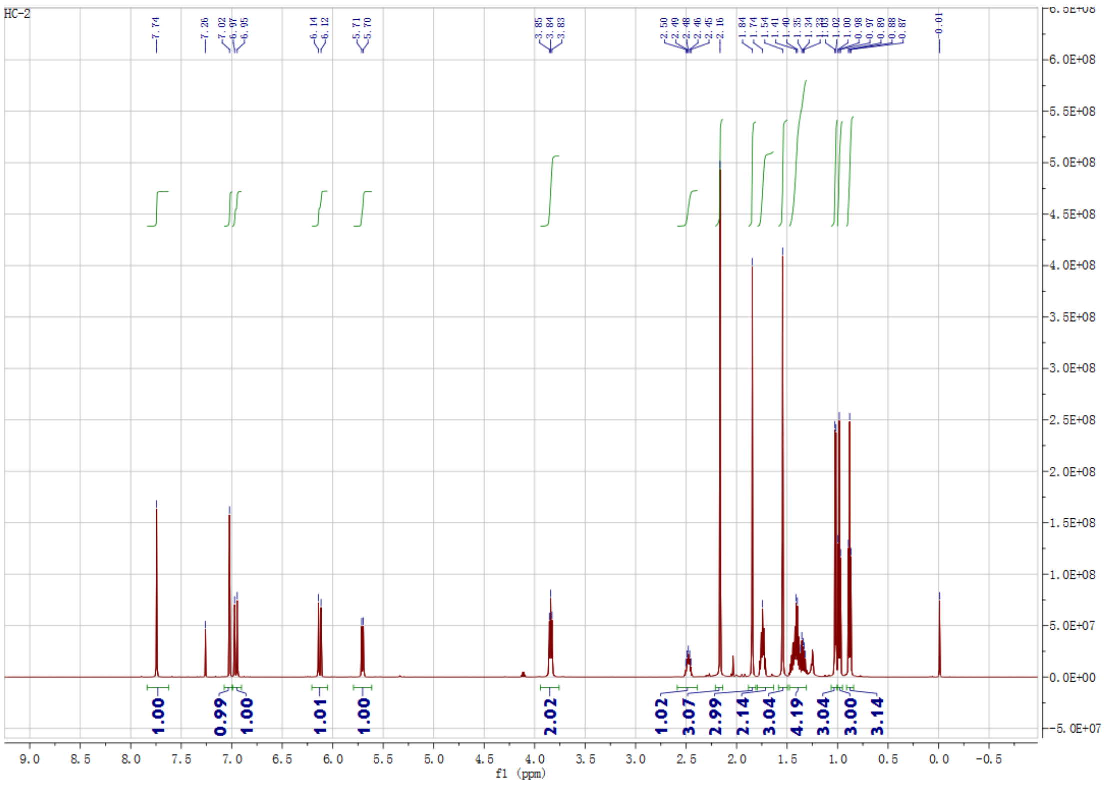

Fig.S5. ^{1}H NMR (600 MHz, $CDCl_3$) spectrum of compound **2**

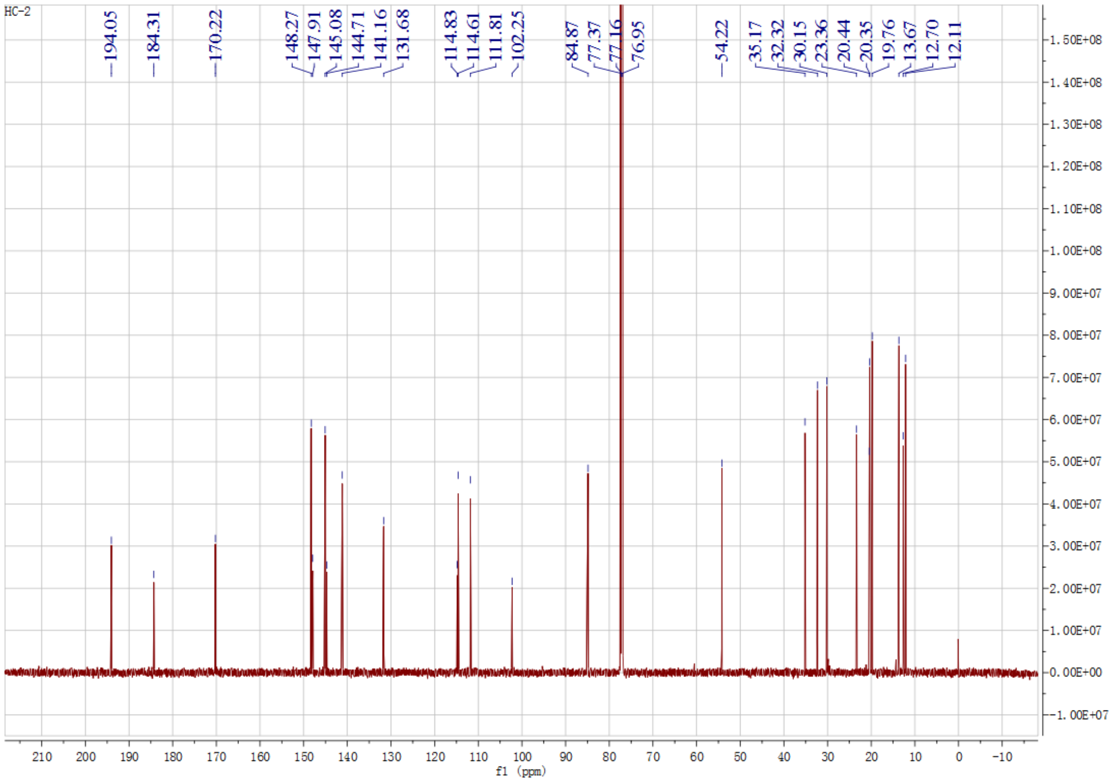

Fig.S6. ^{13}C NMR (150 MHz, $CDCl_3$) spectrum of compound **2**

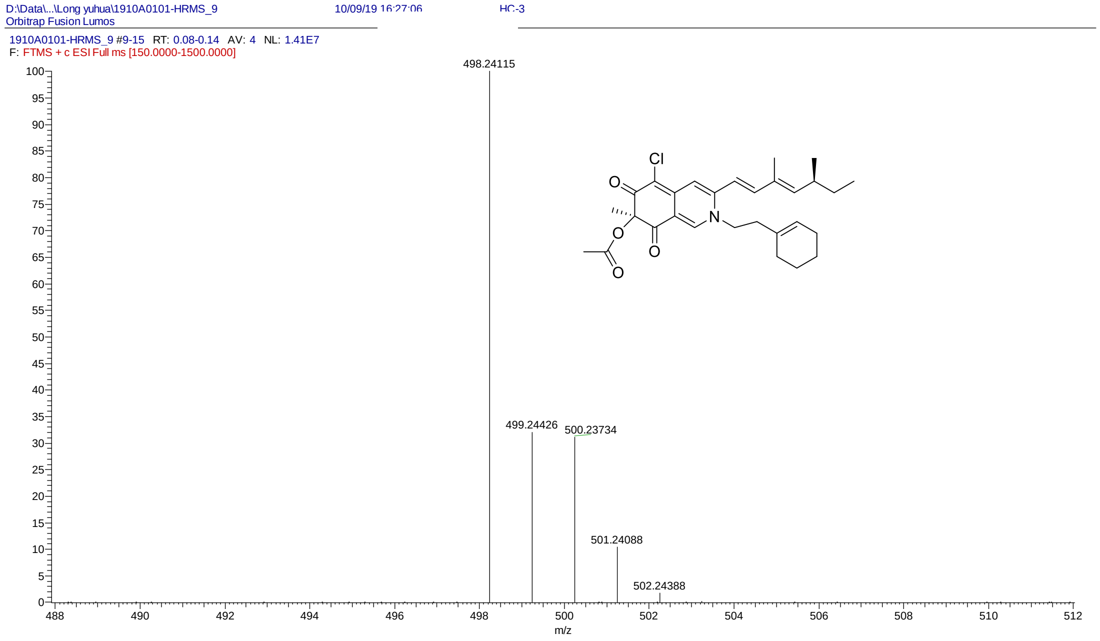

Fig.S7. HRMS spectrum of compound **3**

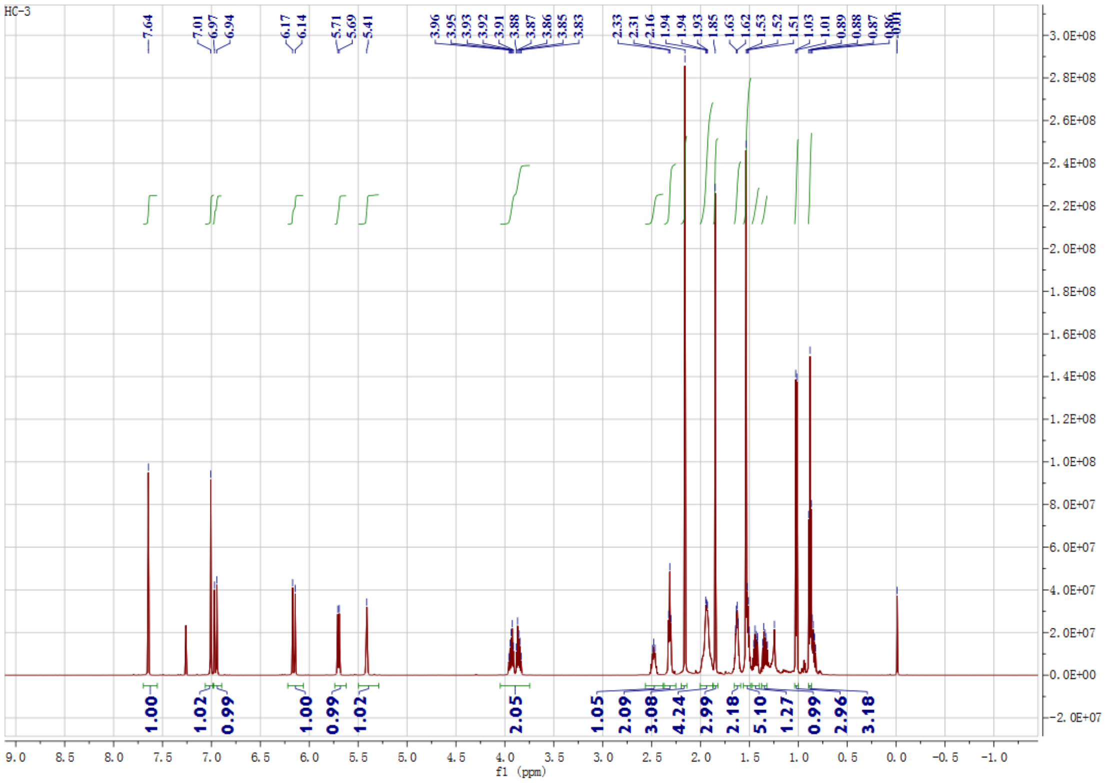

Fig.S8. ^{1}H NMR (600 MHz, $CDCl_3$) spectrum of compound 3

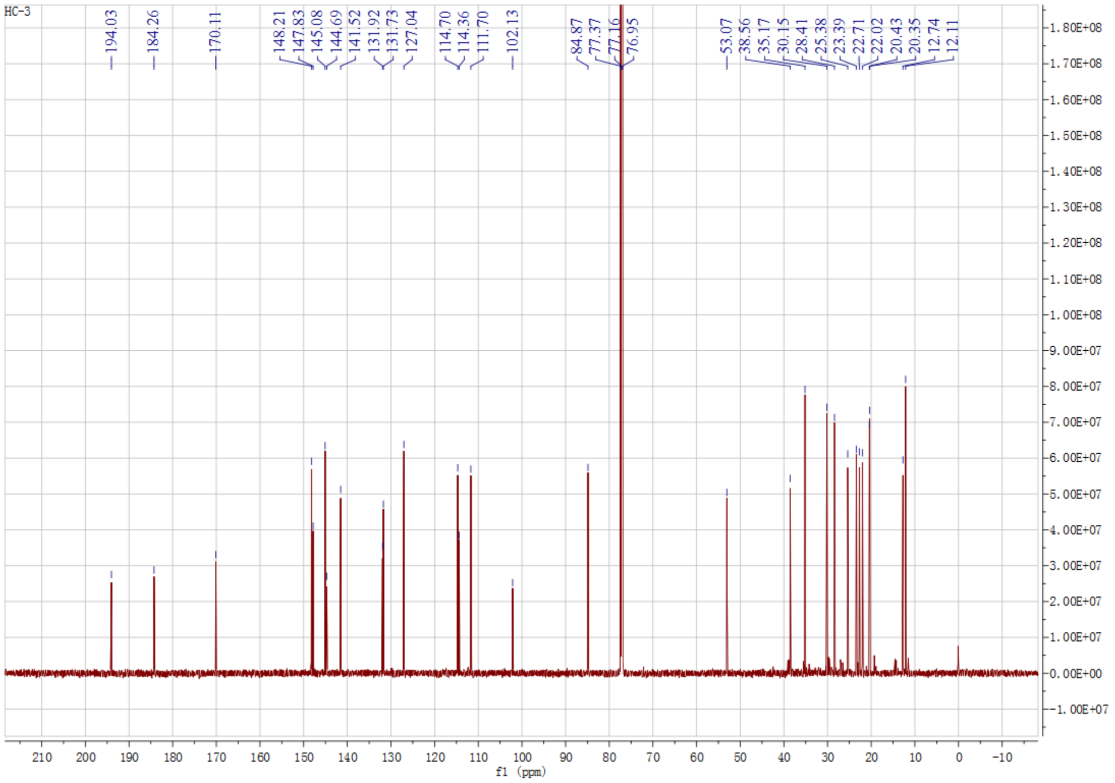

Fig.S9.[13]**C NMR (150 MHz, CDCl3) spectrum of compound 3**

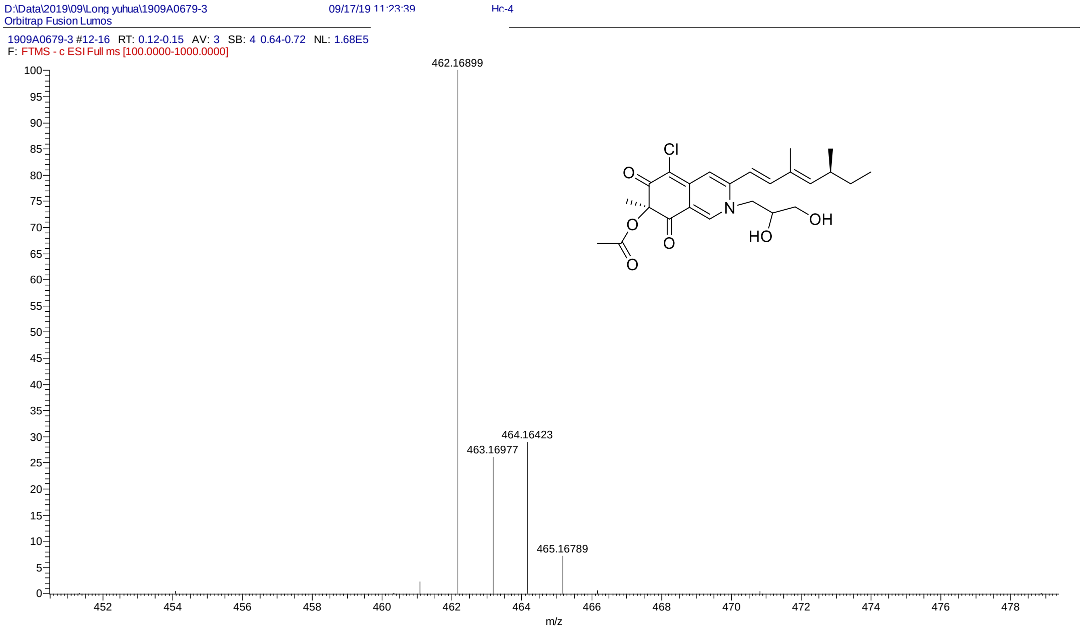

Fig.S10. HRMS spectrum of compound **4**

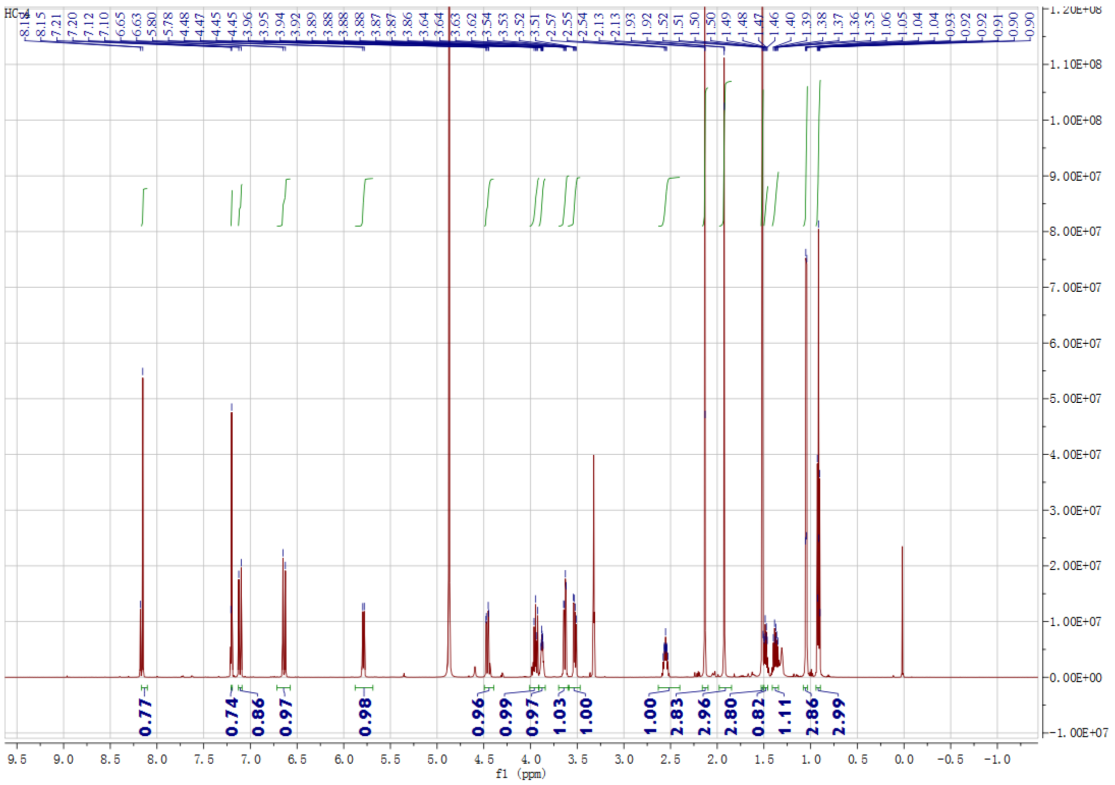

Fig.S11. ^{1}H NMR (600 MHz, CD_3OD) spectrum of compound **4**

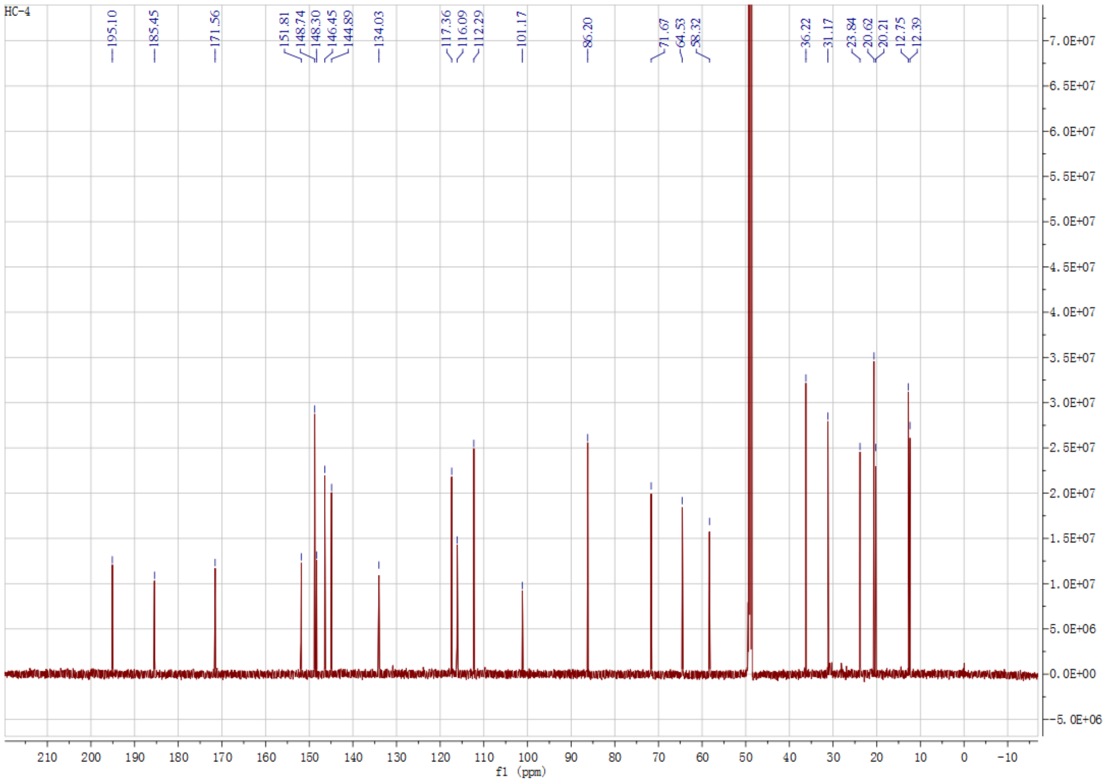

Fig.S12.^{13}C NMR (150 MHz, CD_3OD) spectrum of compound 4

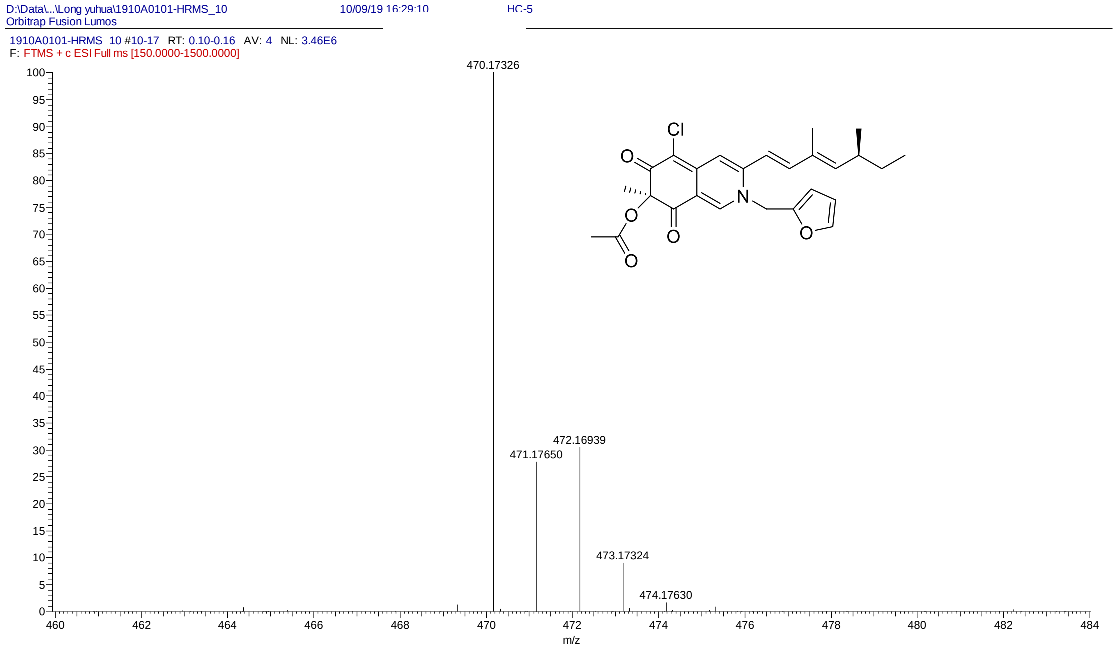

Fig.S13. HRMS spectrum of compound **5**

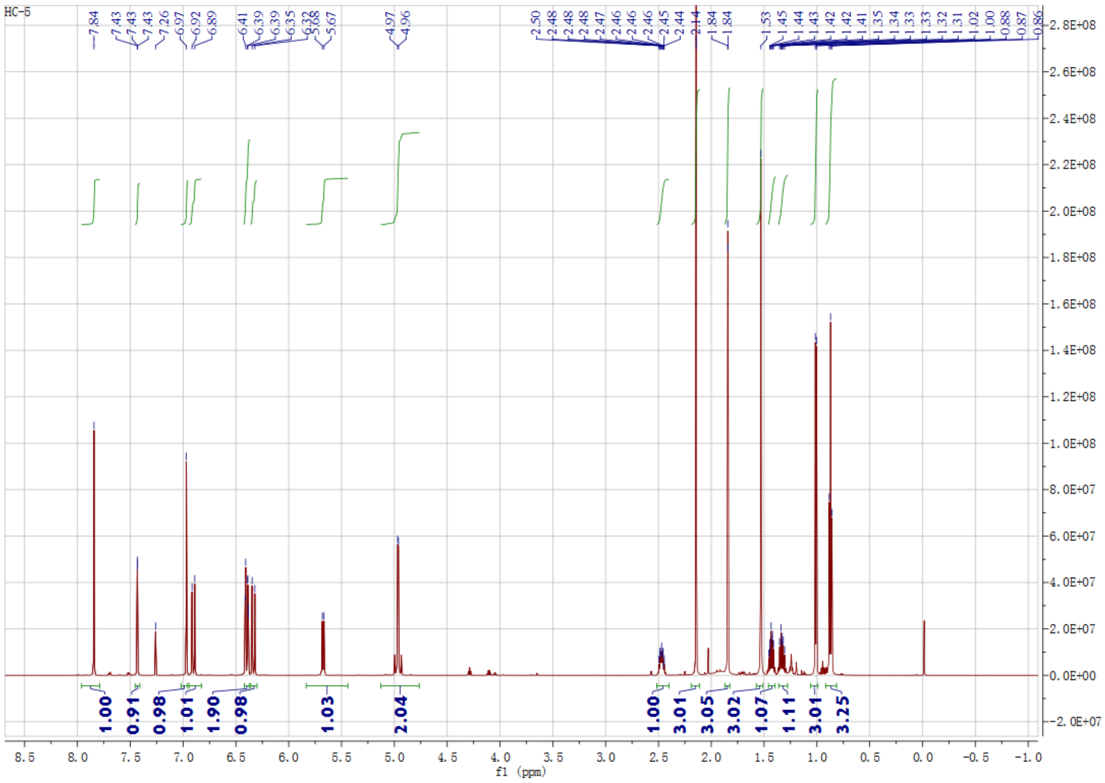

Fig.S14. ^{1}H NMR (600 MHz, $CDCl_3$) spectrum of compound **5**

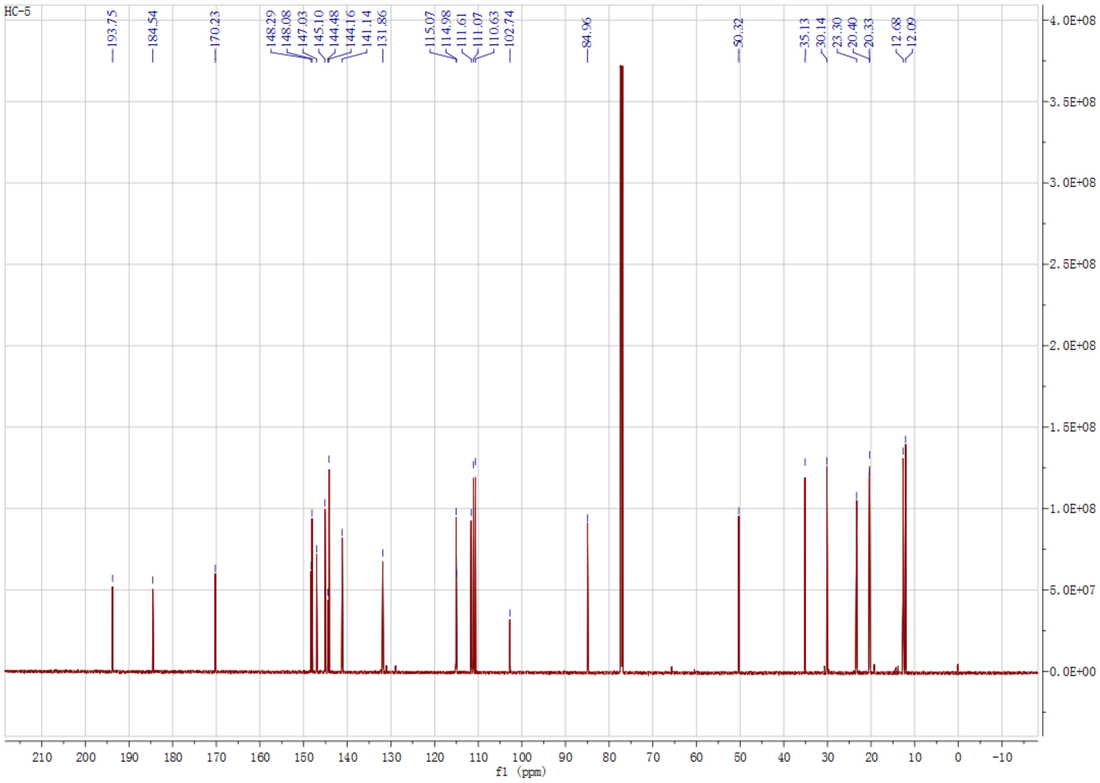

Fig.S15. ^{13}C NMR (150 MHz, $CDCl_3$) spectrum of compound **5**

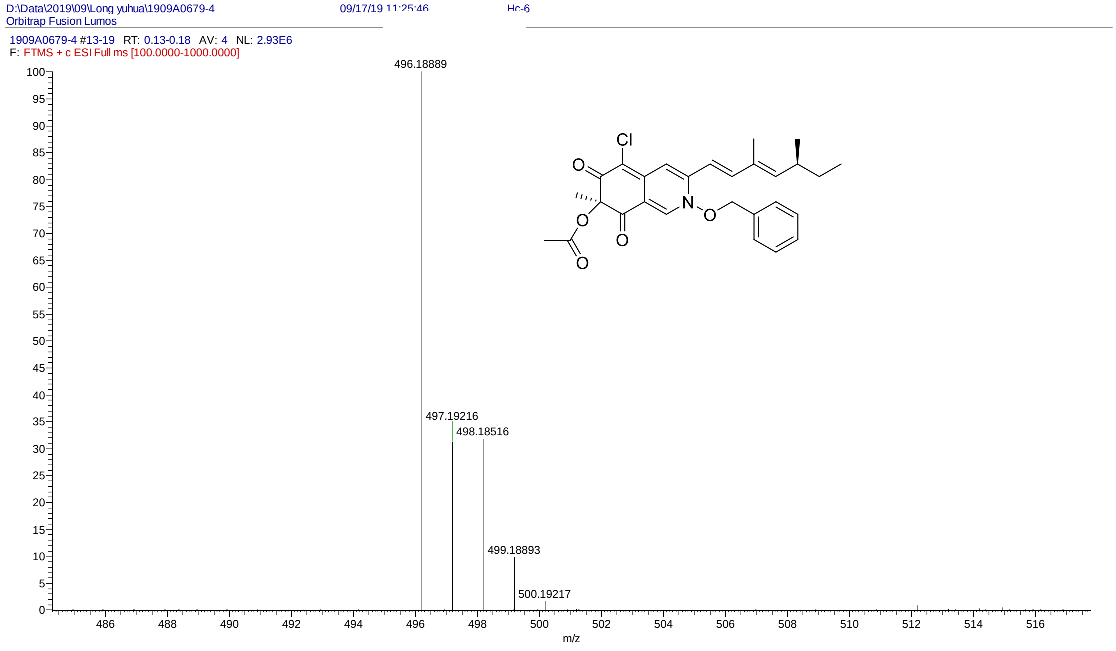

Fig.S16. HRMS spectrum of compound **6**

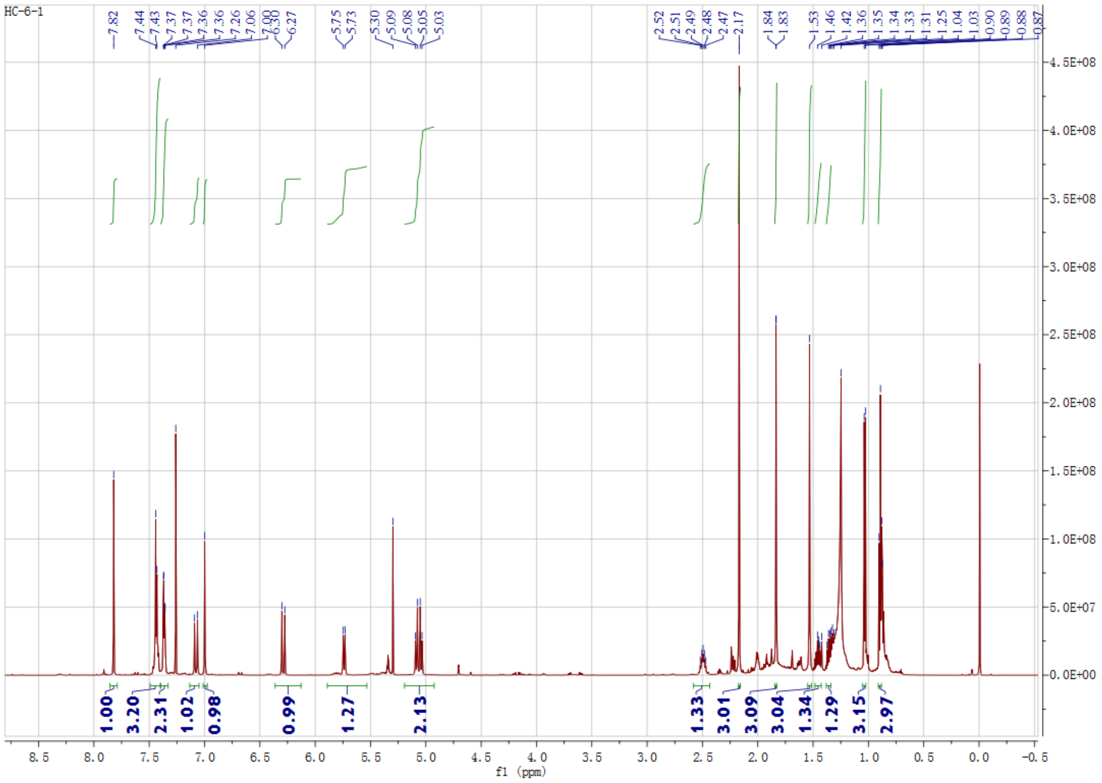

Fig.S17. ^{1}H NMR (600 MHz, $CDCl_3$) spectrum of compound **6**

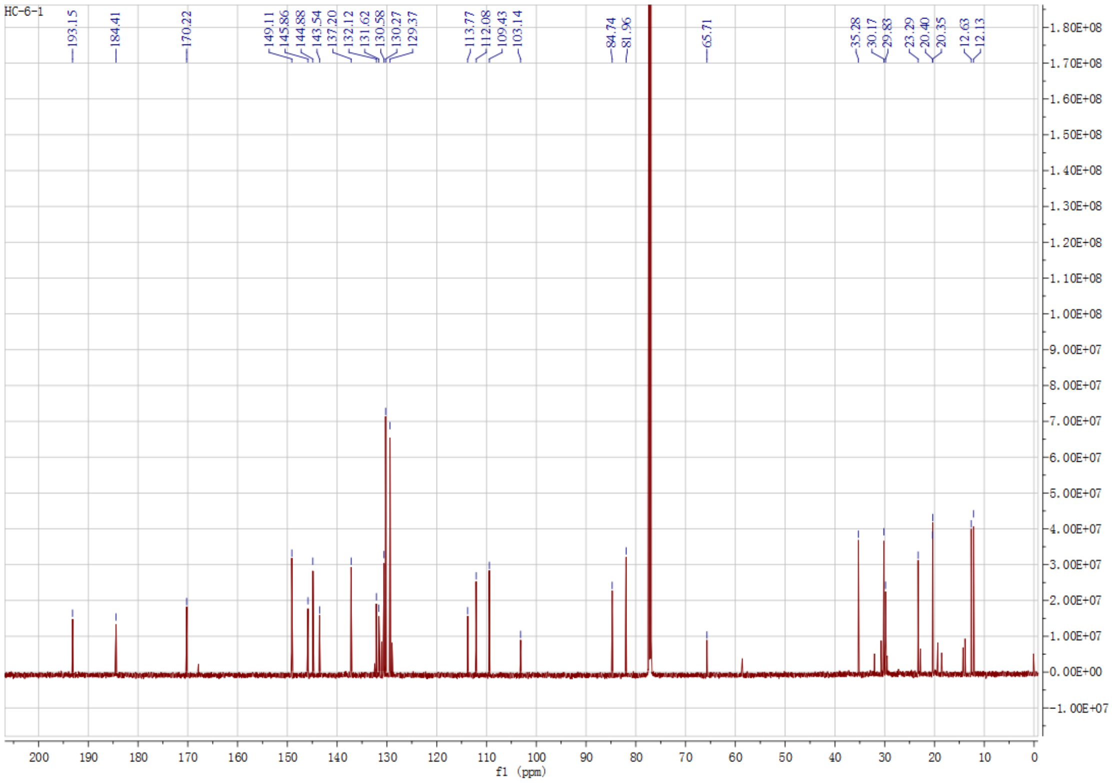

Fig.S18. ^{13}C NMR (150 MHz, $CDCl_3$) spectrum of compound **6**

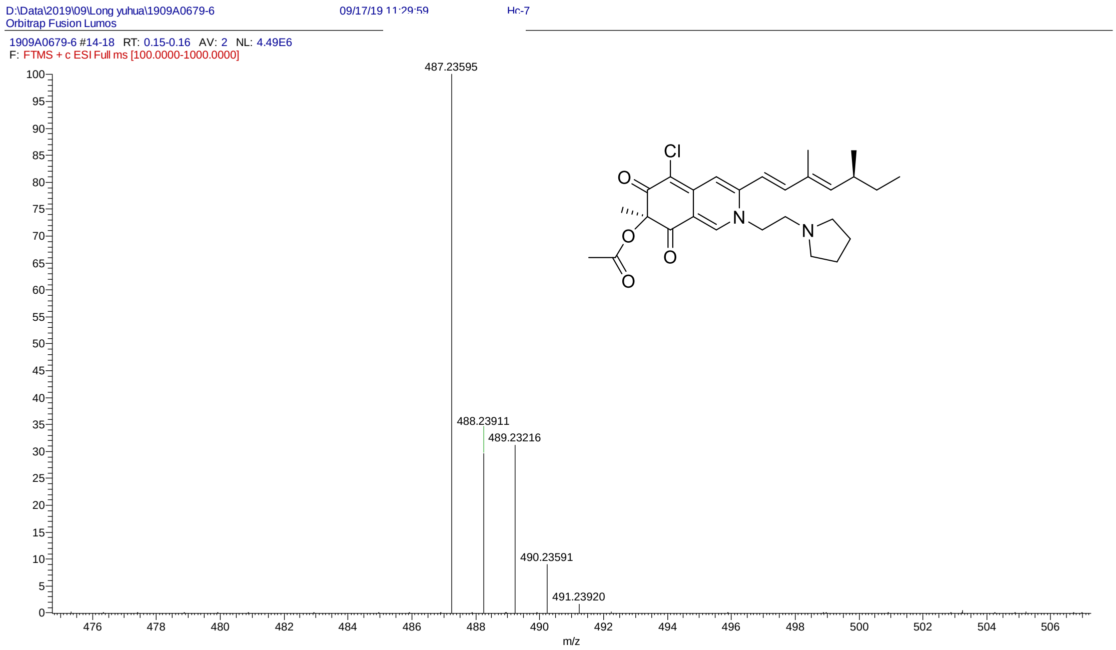

Fig.S19. HRMS spectrum of compound **7**

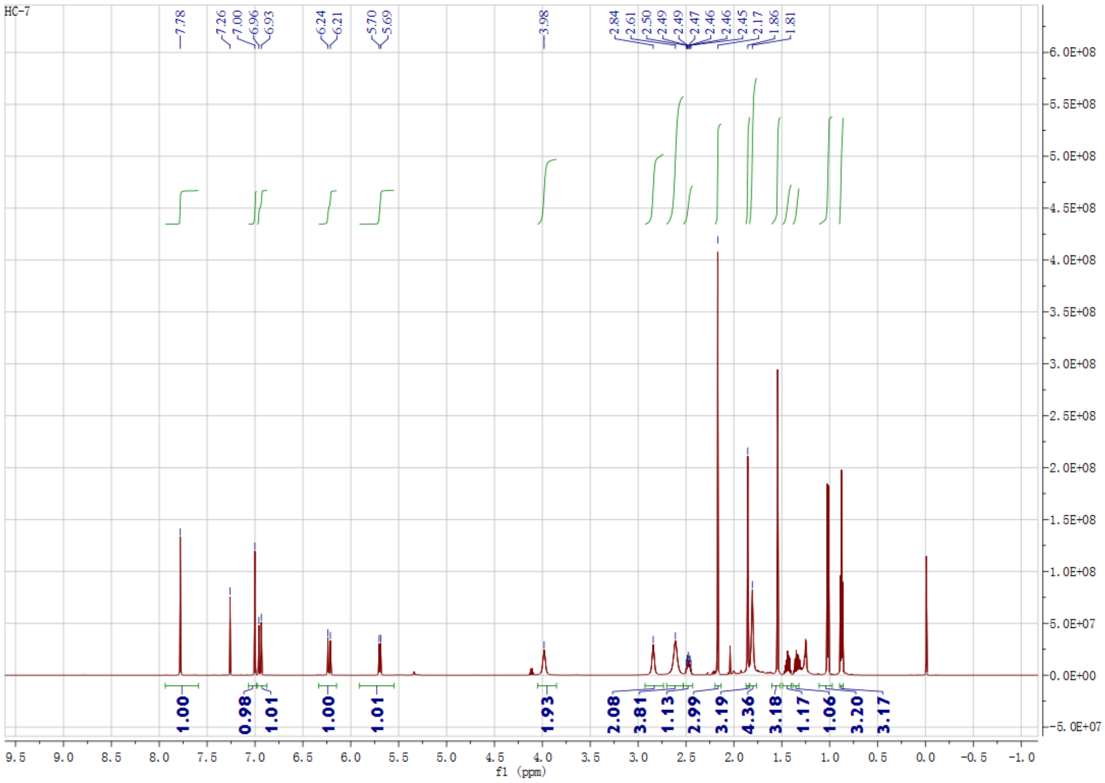

Fig.S20. ^{1}H NMR (600 MHz, $CDCl_3$) spectrum of compound **7**

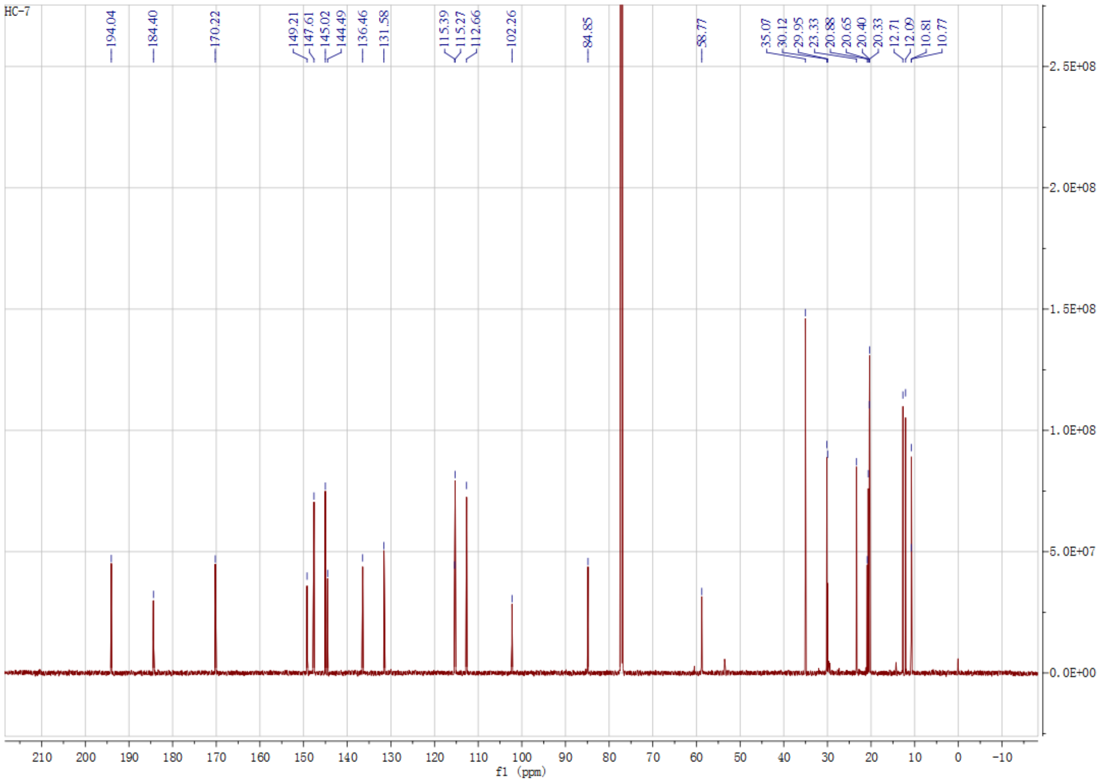

Fig.S21. ^{13}C NMR (150 MHz, $CDCl_3$) spectrum of compound **7**

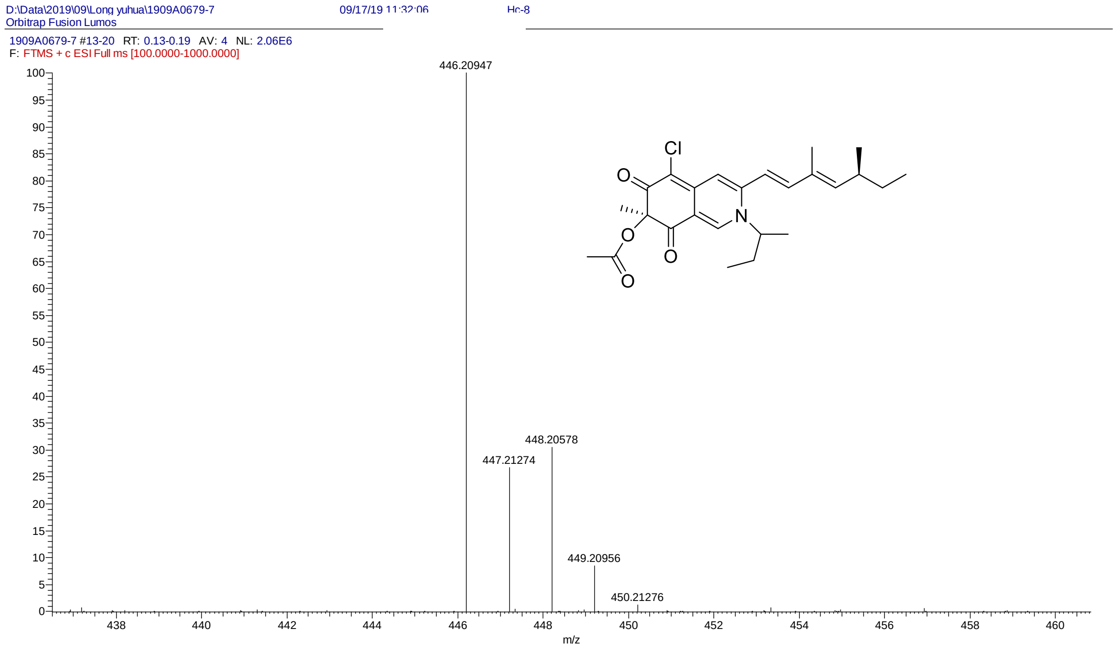

Fig.S22. HRMS spectrum of compound **8**

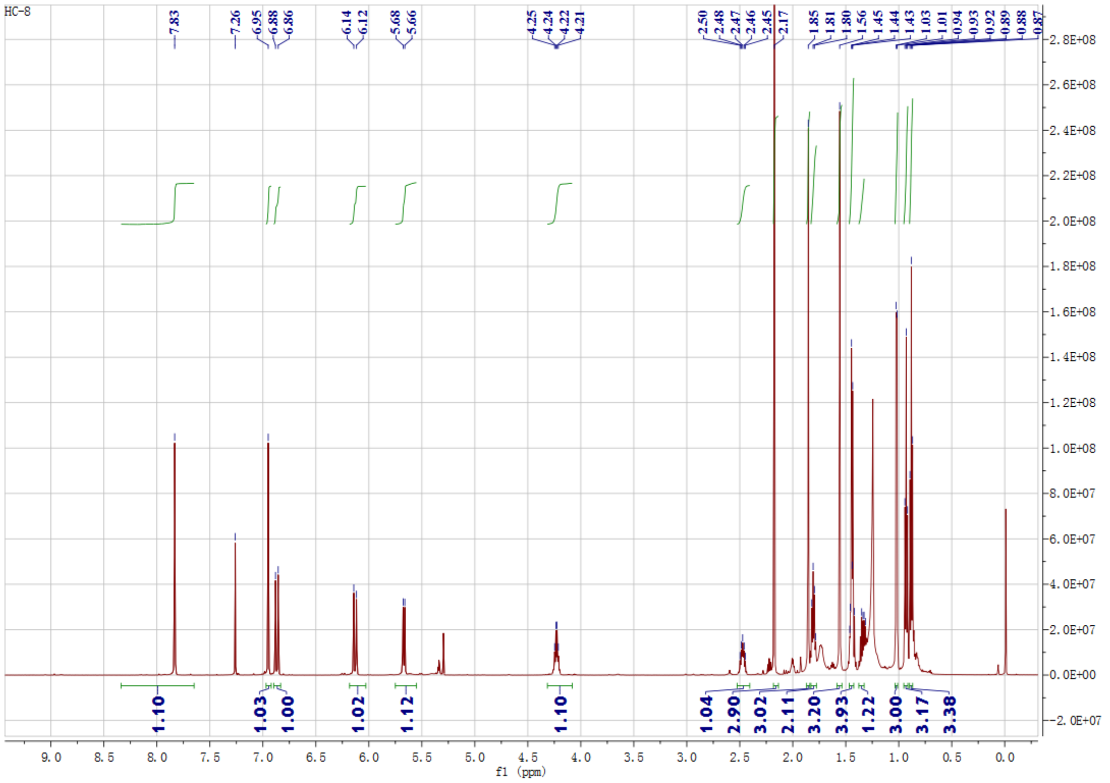

Fig.S23. ^{1}H NMR (600 MHz, $CDCl_3$) spectrum of compound **8**

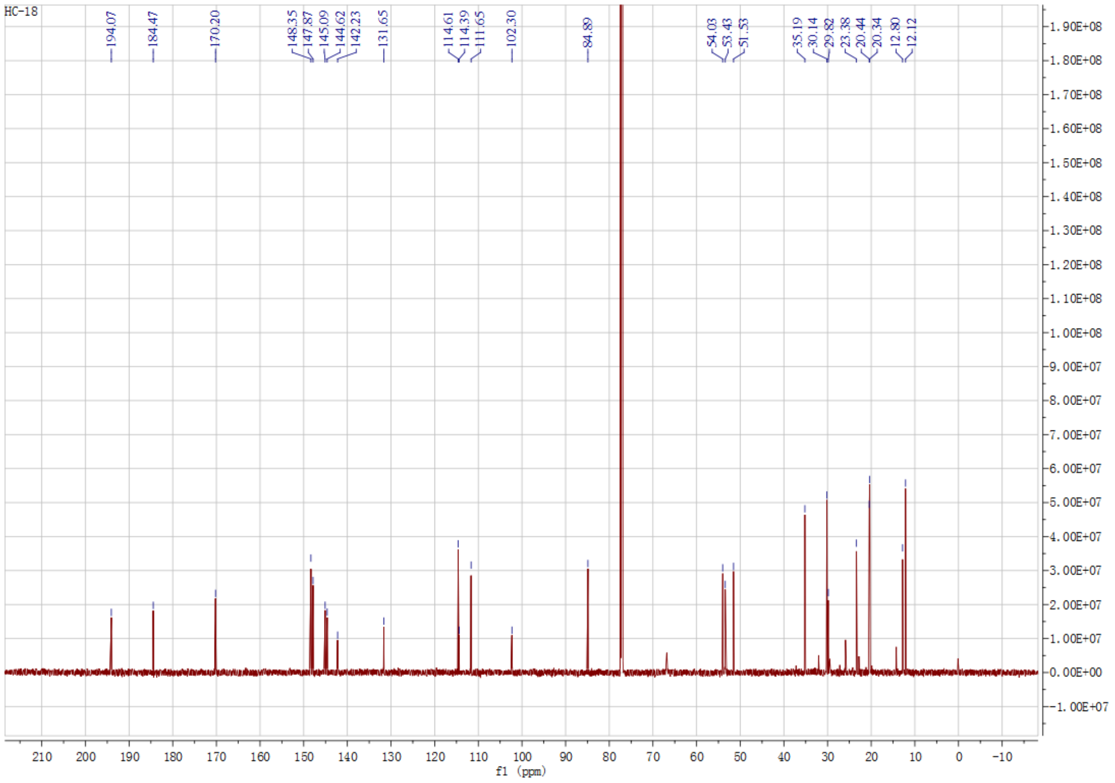

Fig.S24. ^{13}C NMR (150 MHz, $CDCl_3$) spectrum of compound **8**

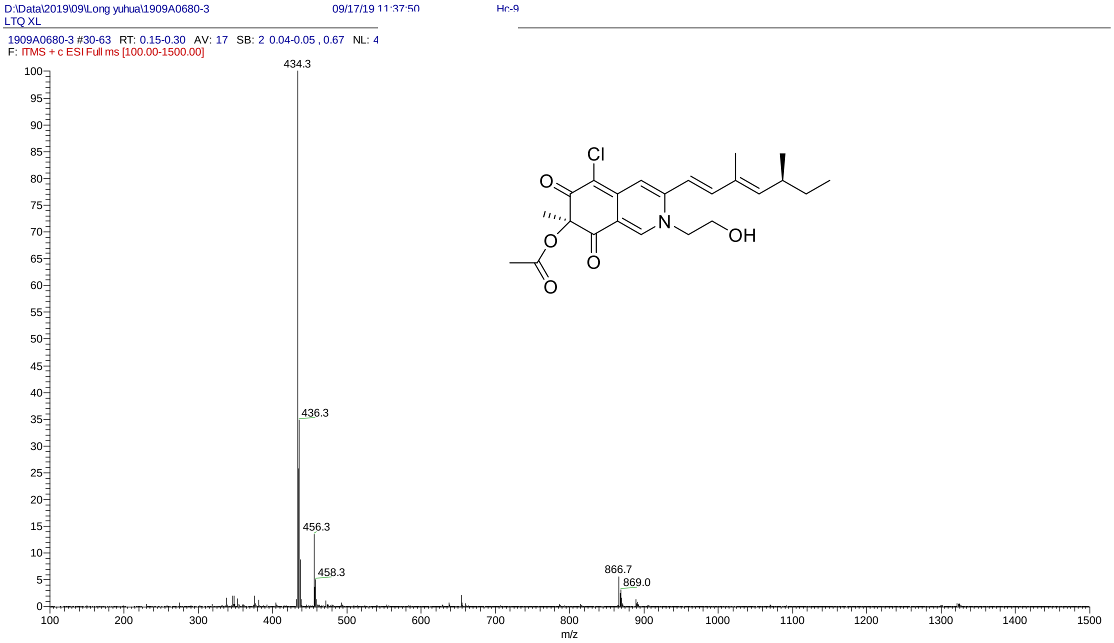

Fig.S25. MS spectrum of compound **9**

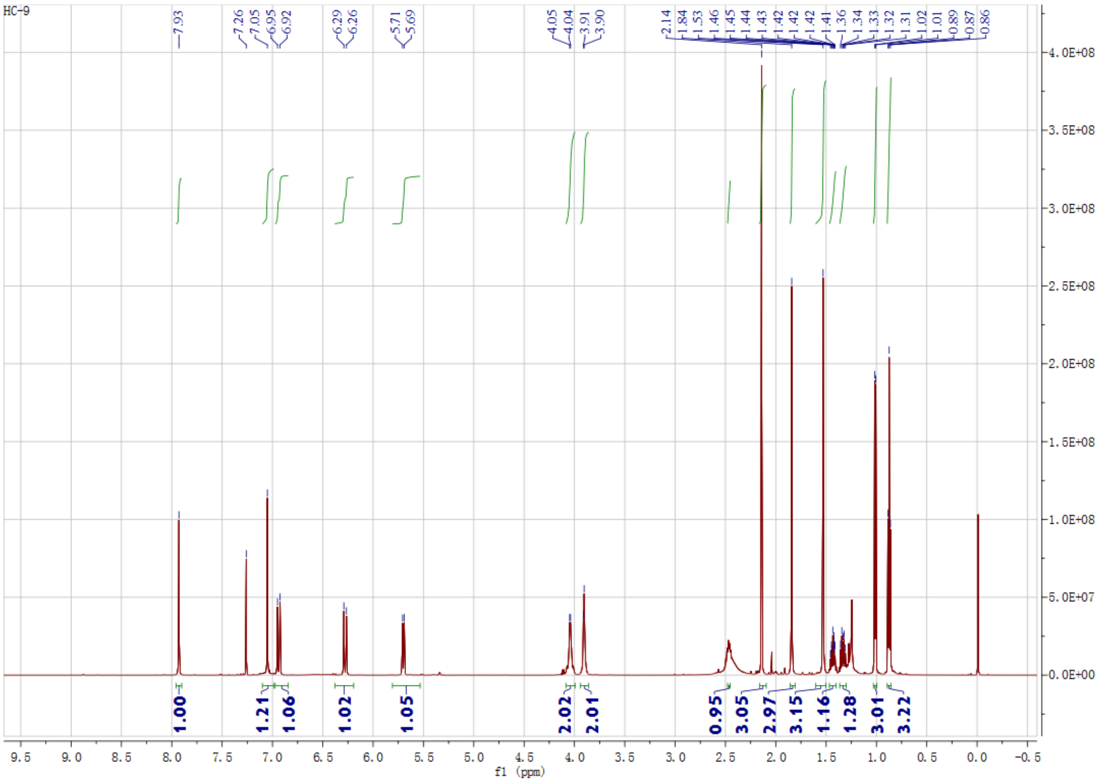

Fig.S26. ^{1}H NMR (600 MHz, $CDCl_3$) spectrum of compound **9**

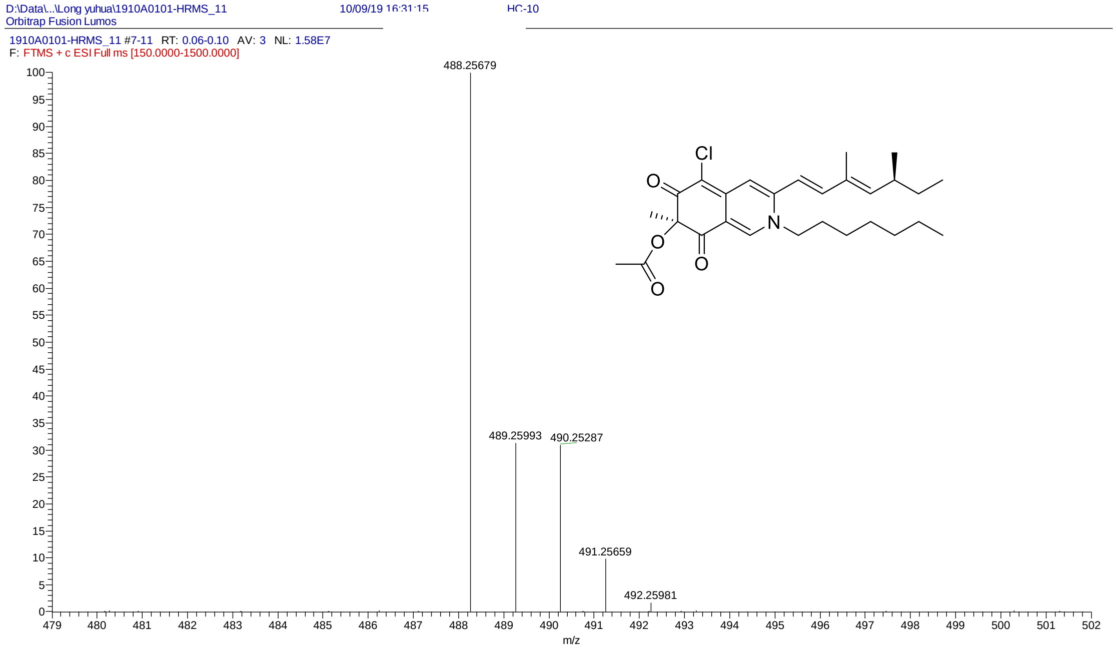

Fig.S27. HRMS spectrum of compound **10**

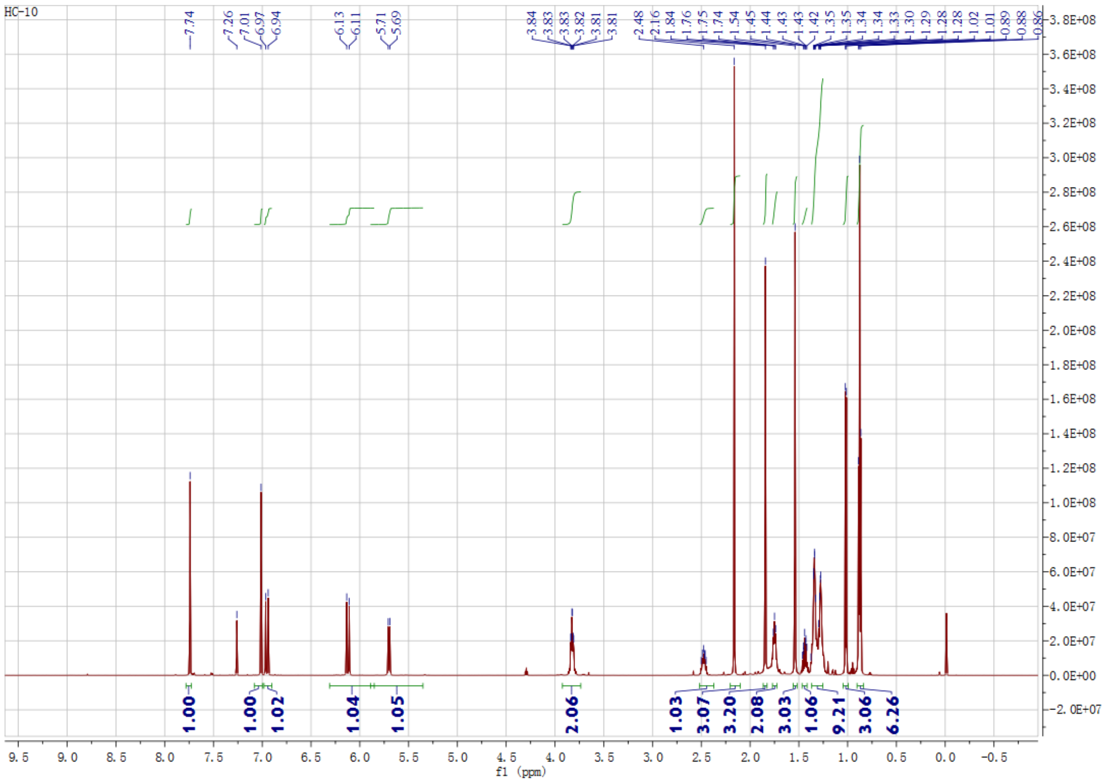

Fig.S28. ^{1}H NMR (600 MHz, $CDCl_3$) spectrum of compound **10**

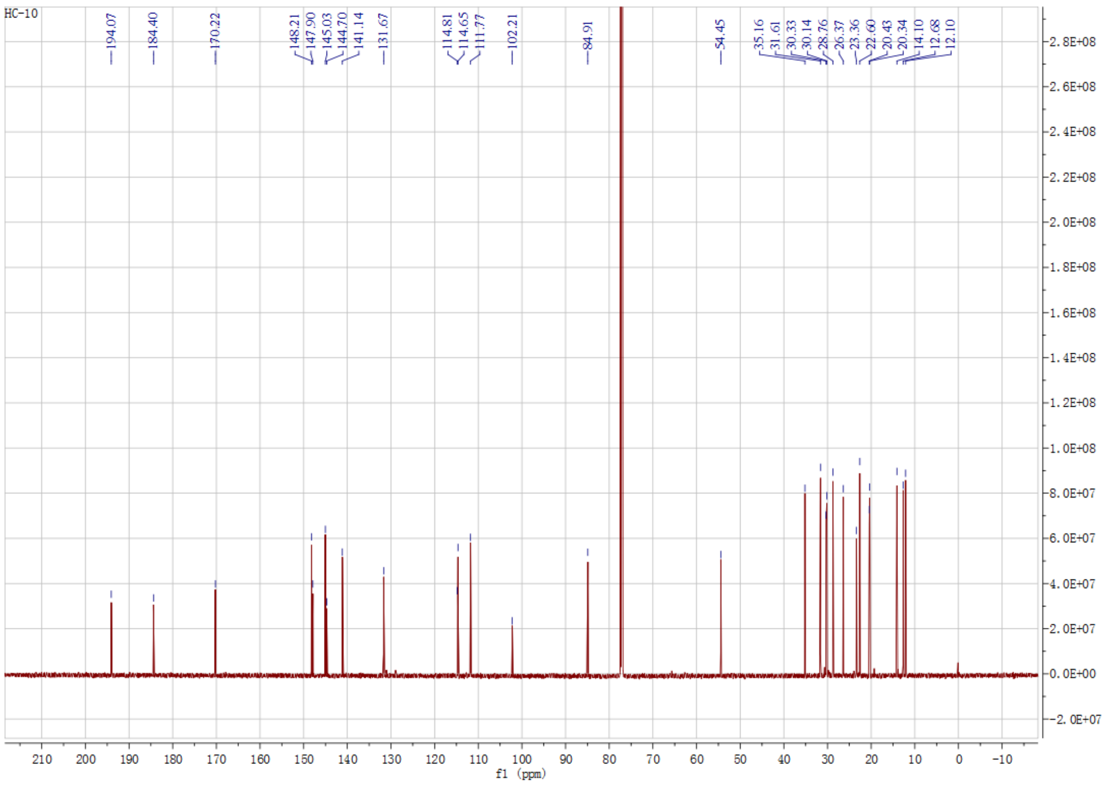

Fig.S29. ^{13}C NMR (150 MHz, $CDCl_3$) spectrum of compound **10**

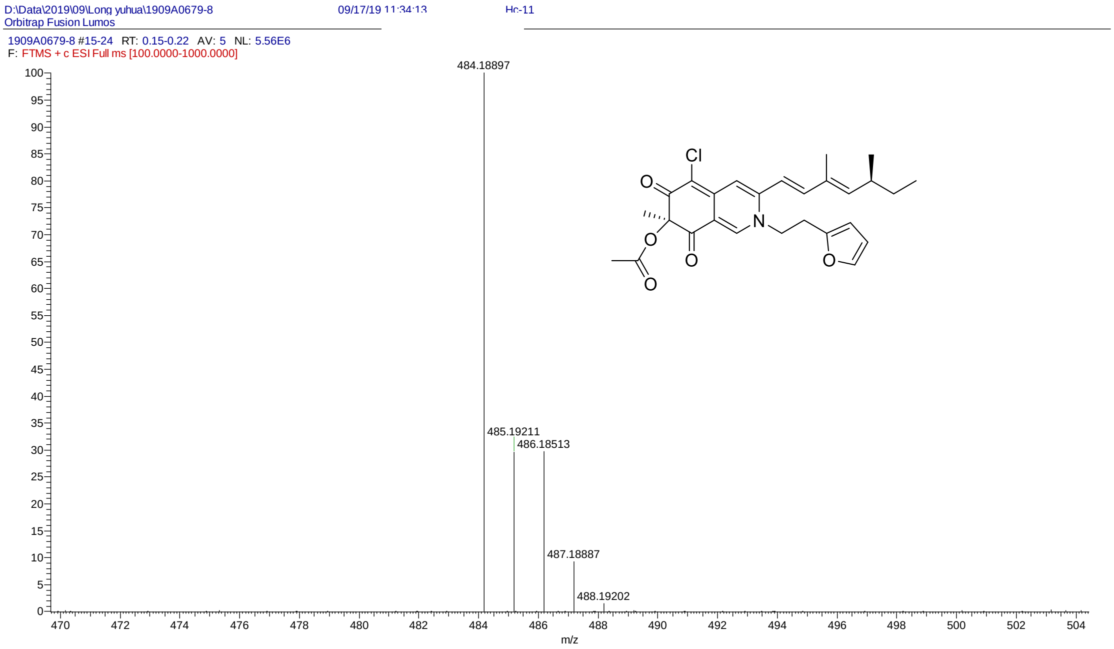

Fig.S30. HRMS spectrum of compound **11**

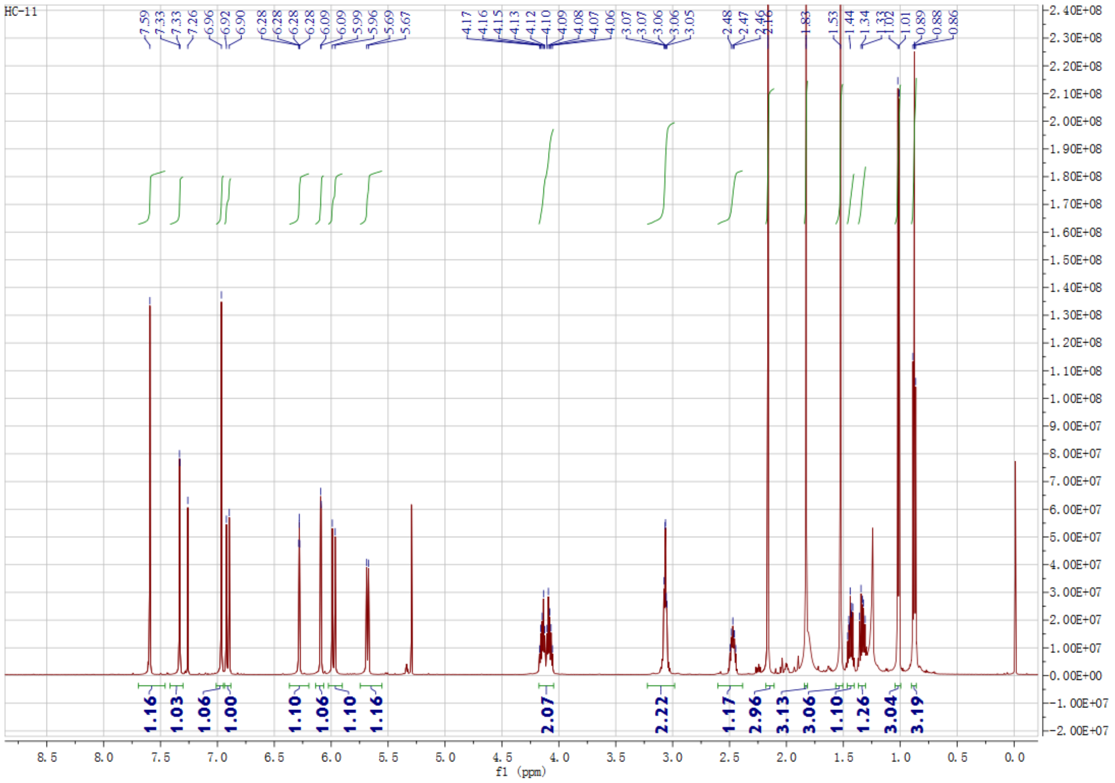

Fig.S31. ^{1}H NMR (600 MHz, $CDCl_3$) spectrum of compound **11**

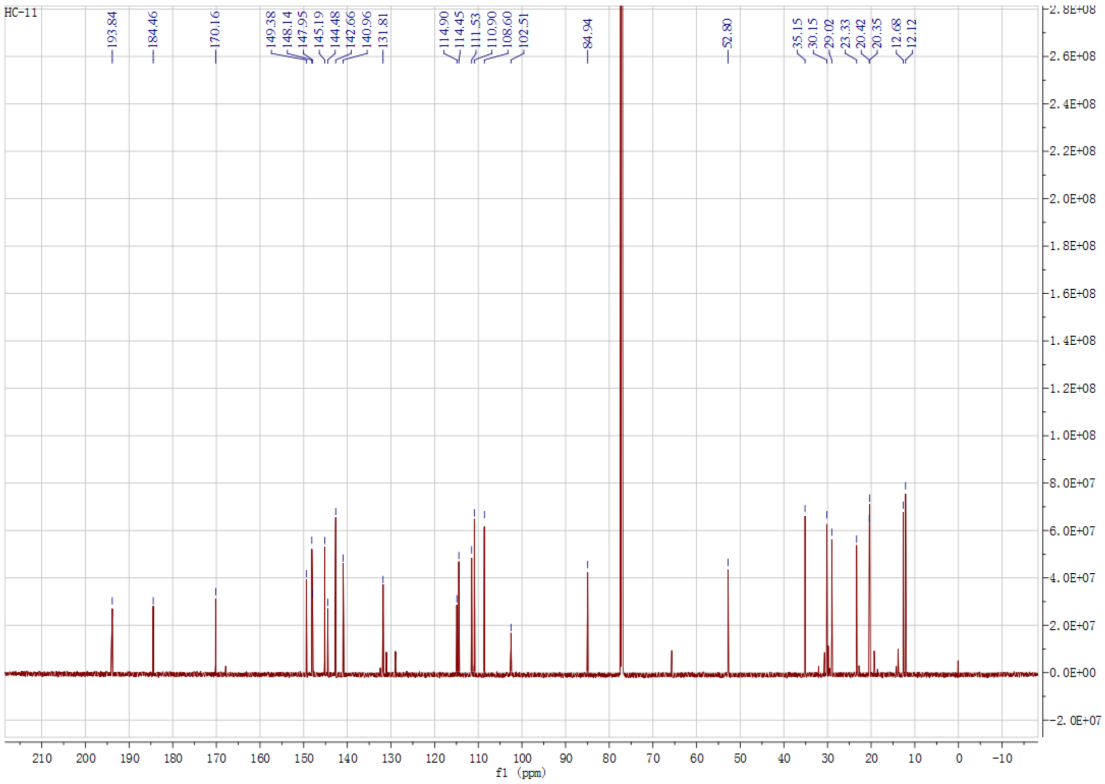

Fig.S32. ^{13}C NMR (150 MHz, $CDCl_3$) spectrum of compound **11**

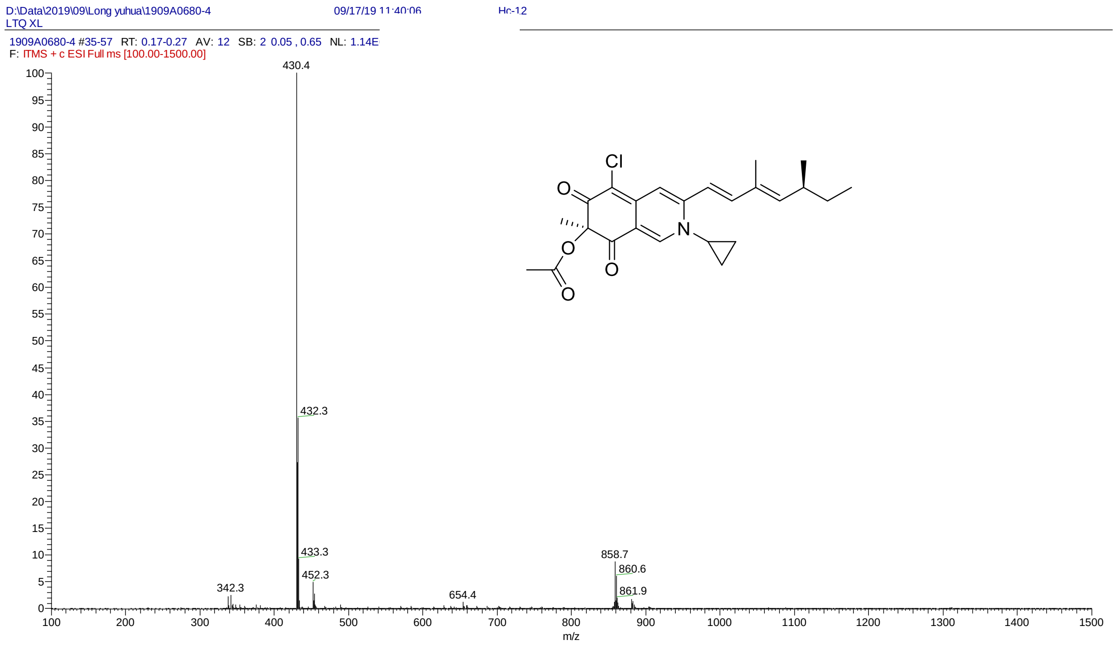

Fig.S33. MS spectrum of compound **12**

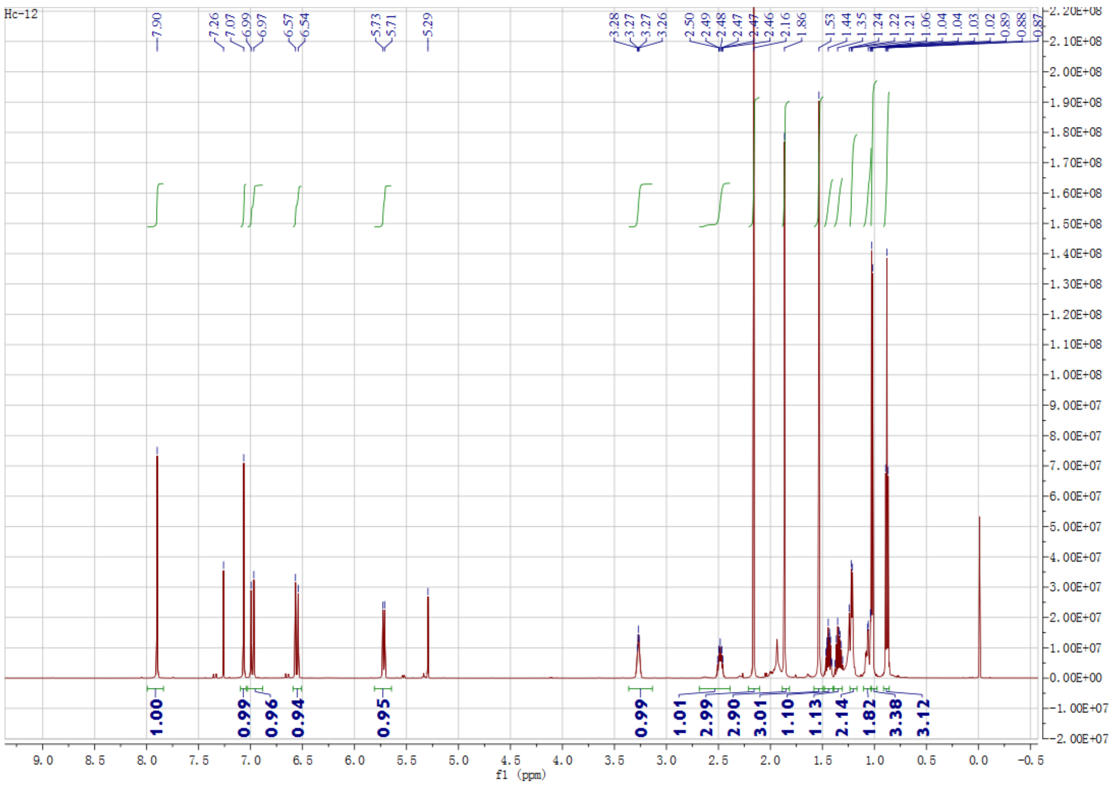

Fig.S34. ^{1}H NMR (600 MHz, $CDCl_3$) spectrum of compound **12**

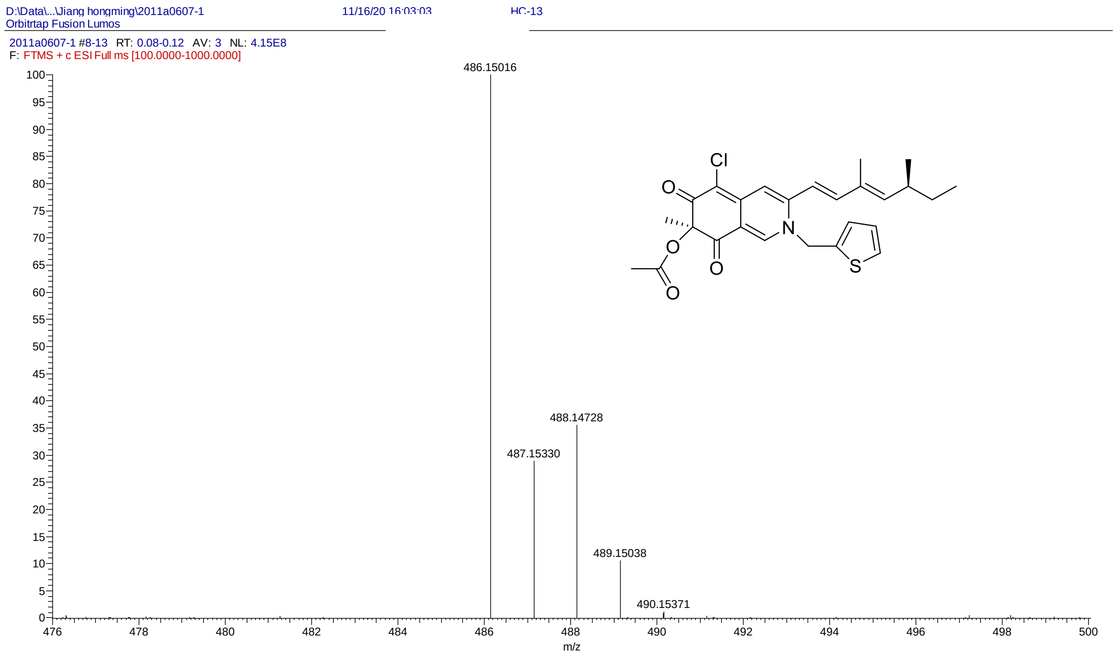

Fig.S35. HRMS spectrum of compound **13**

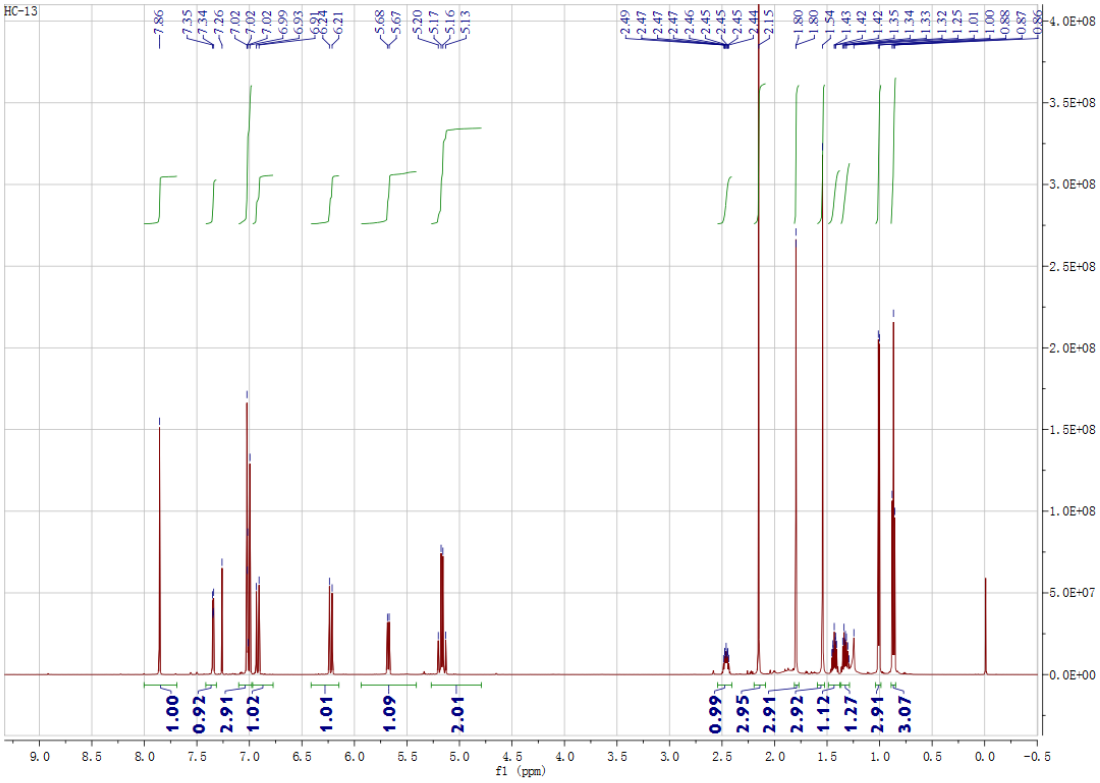

Fig.S36. ^{1}H NMR (600 MHz, $CDCl_3$) spectrum of compound **13**

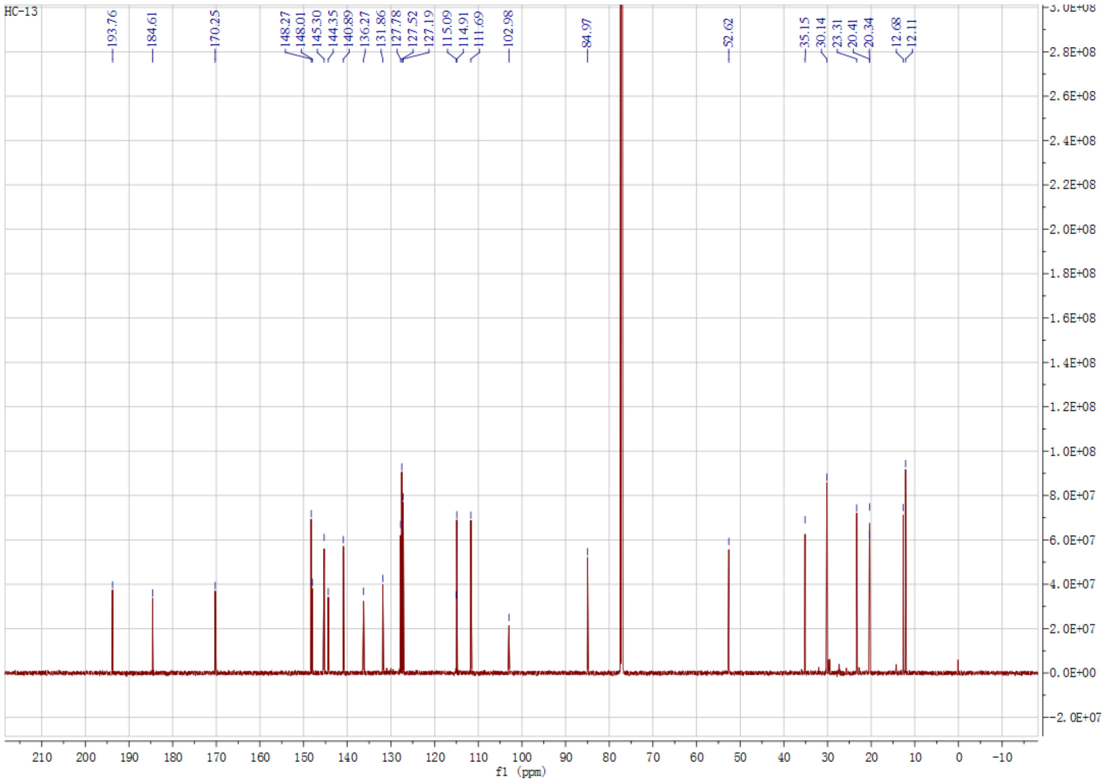

Fig.S37. ^{13}C **NMR (150 MHz, CDCl$_3$) spectrum of compound 13**

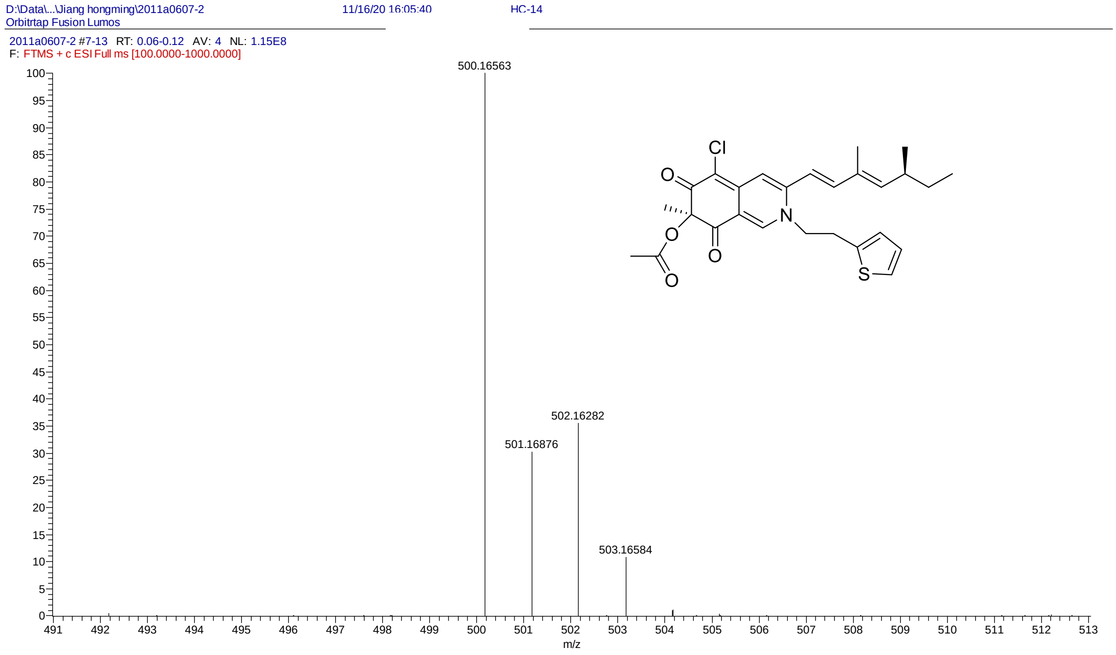

Fig.S38. HRMS spectrum of compound **14**

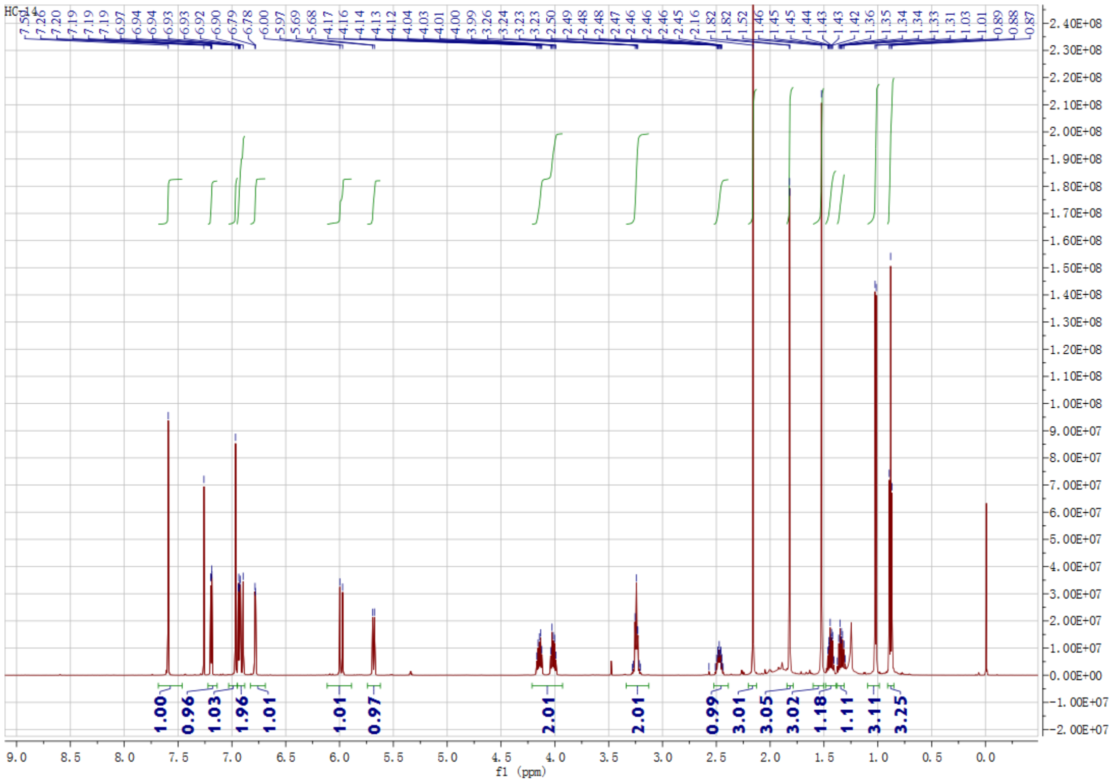

Fig.S39. ^{1}H NMR (600 MHz, $CDCl_3$) spectrum of compound **14**

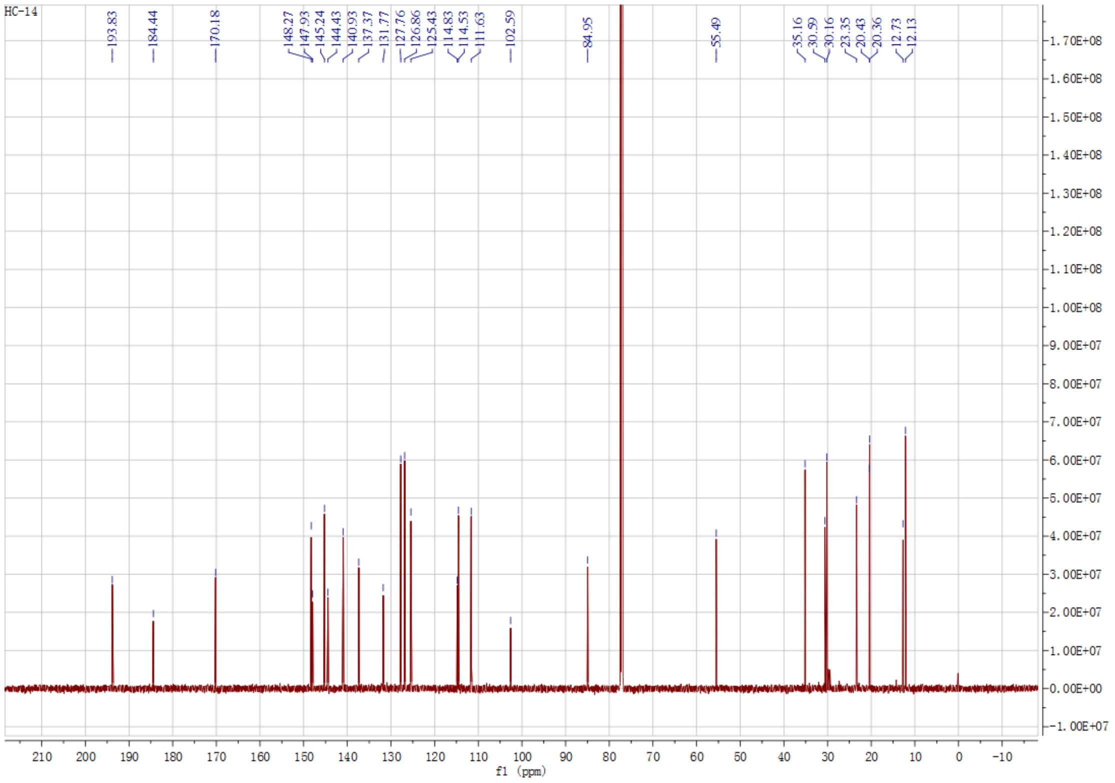

Fig.S40. ^{13}C NMR (150 MHz, $CDCl_3$) spectrum of compound 14

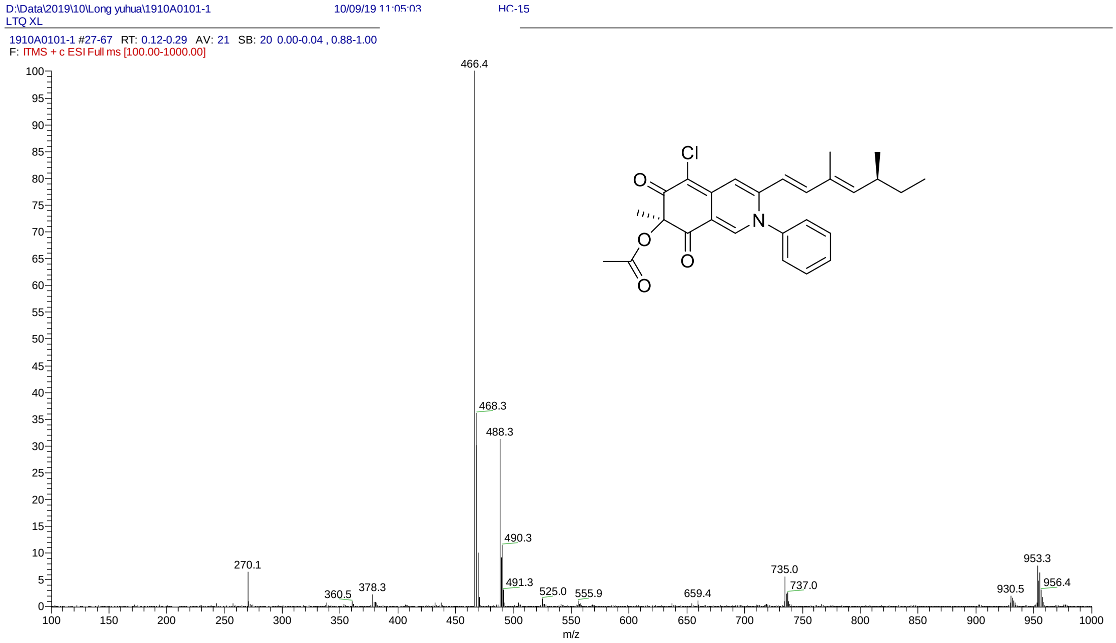

Fig.S41. MS spectrum of compound **15**

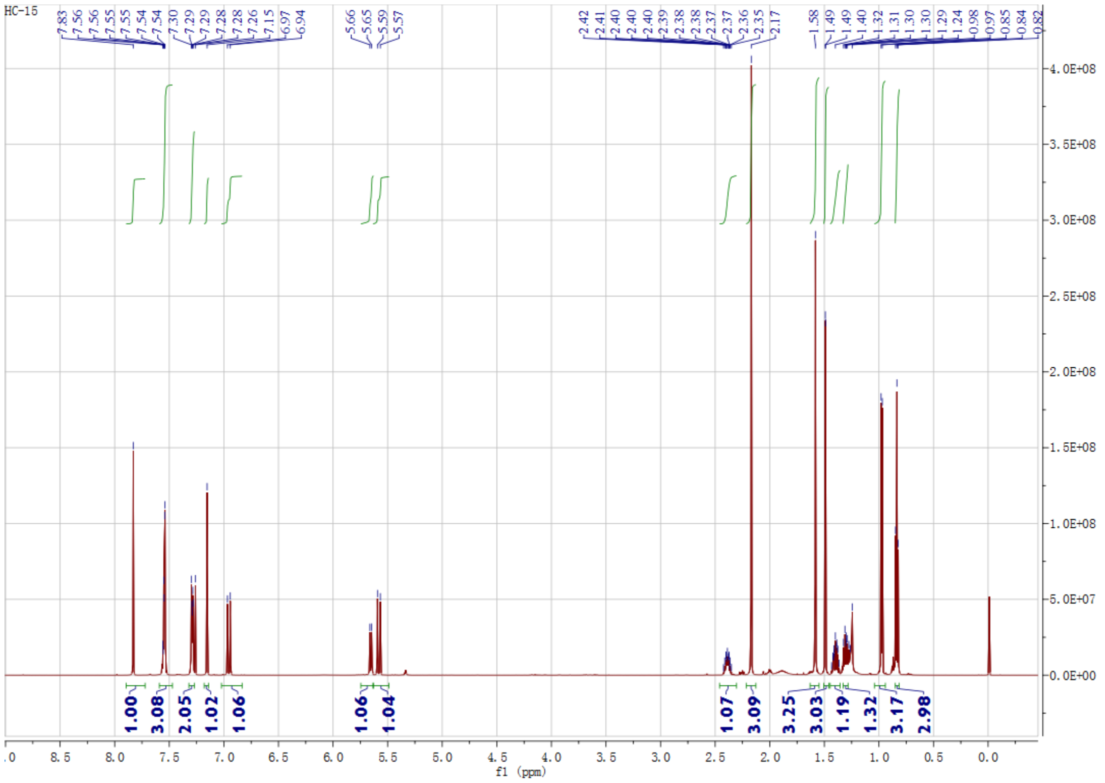

Fig.S42. ^{1}H NMR (600 MHz, $CDCl_3$) spectrum of compound **15**

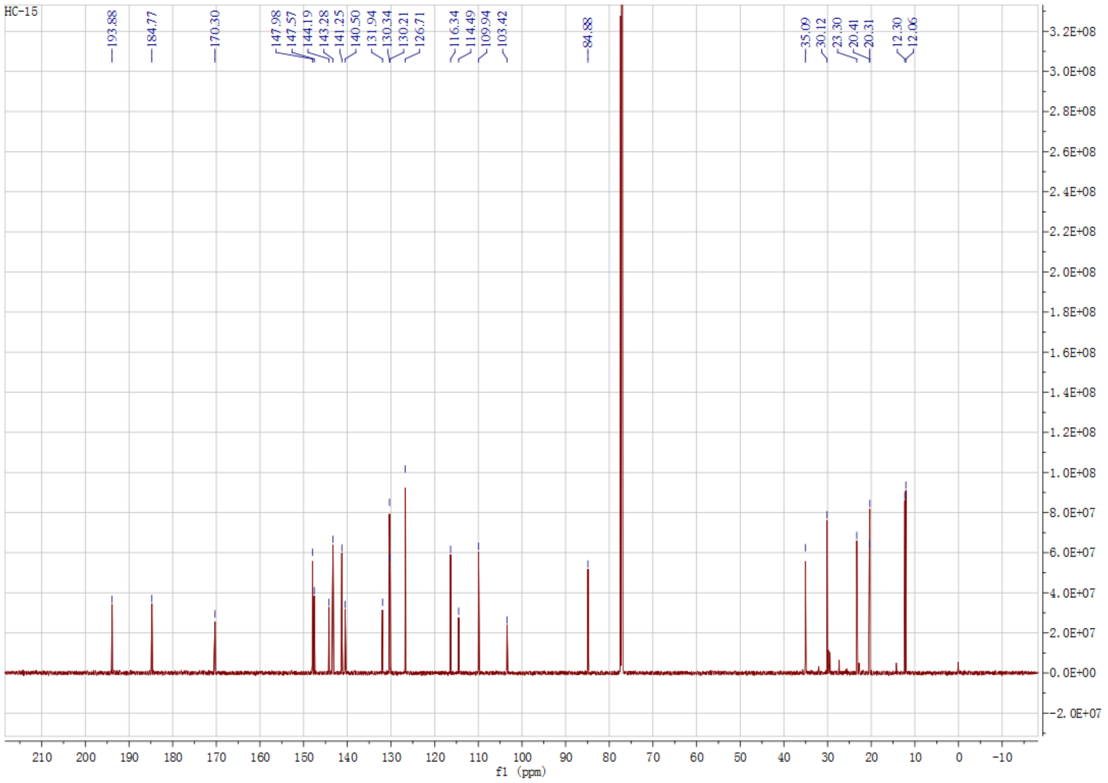

Fig.S43. ^{13}C NMR (150 MHz, $CDCl_3$) spectrum of compound 15

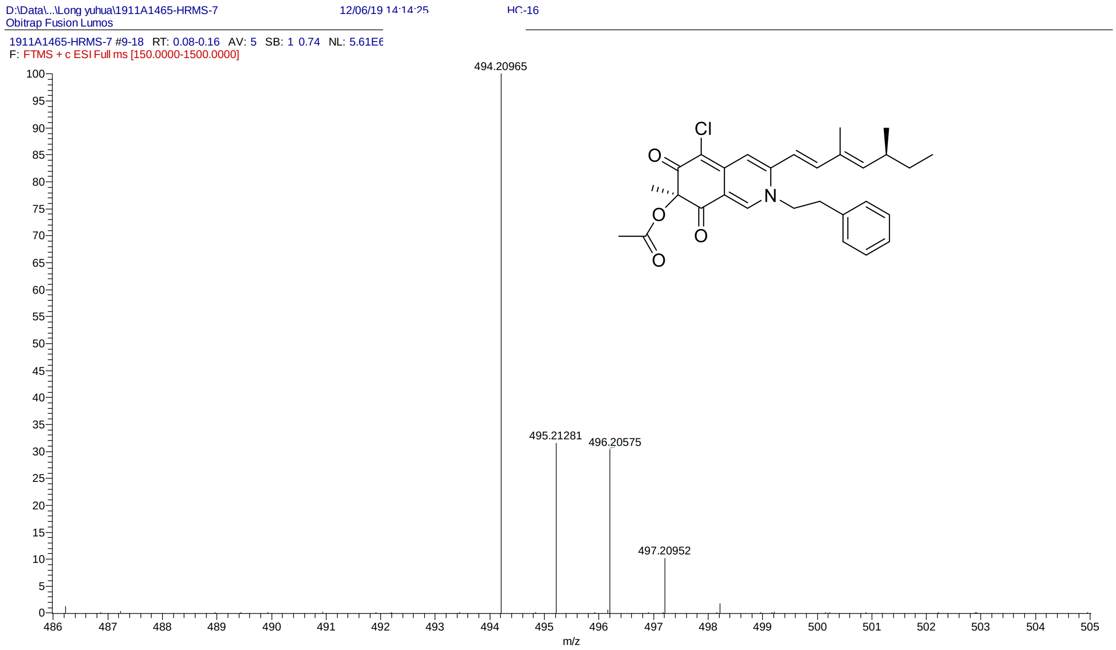

Fig.S44. MS spectrum of compound **16**

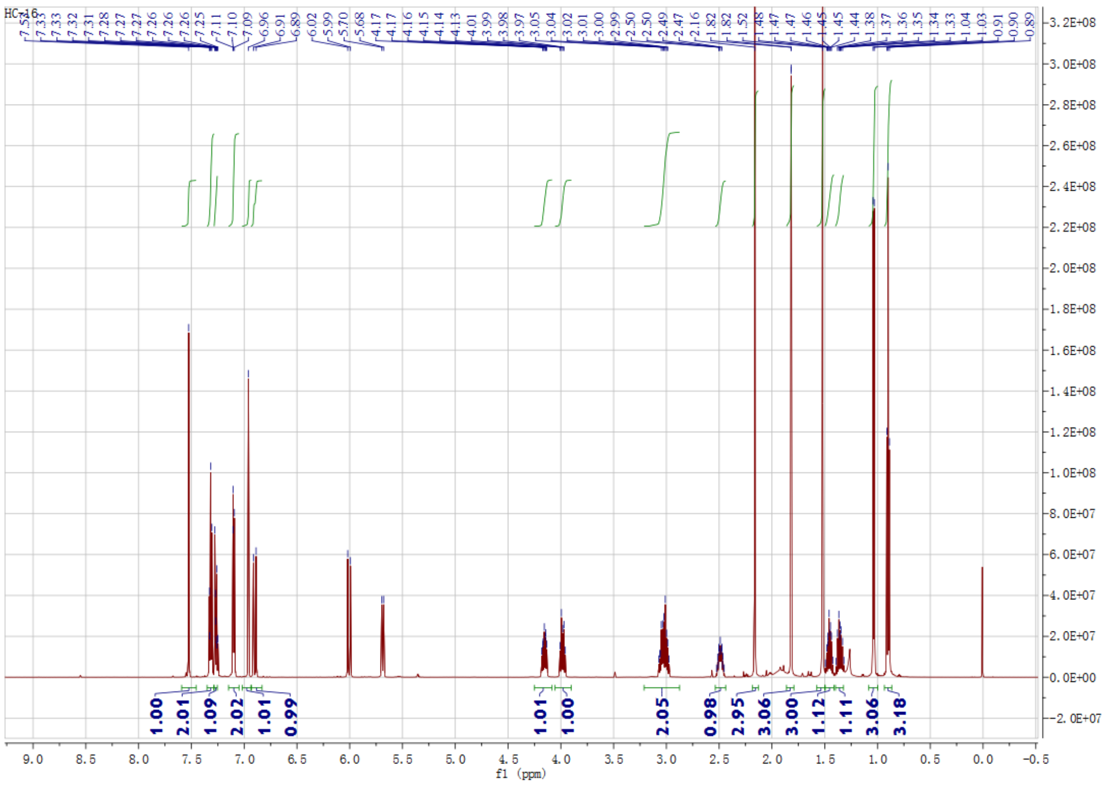

Fig.S45. ^{1}H NMR (600 MHz, $CDCl_3$) spectrum of compound **16**

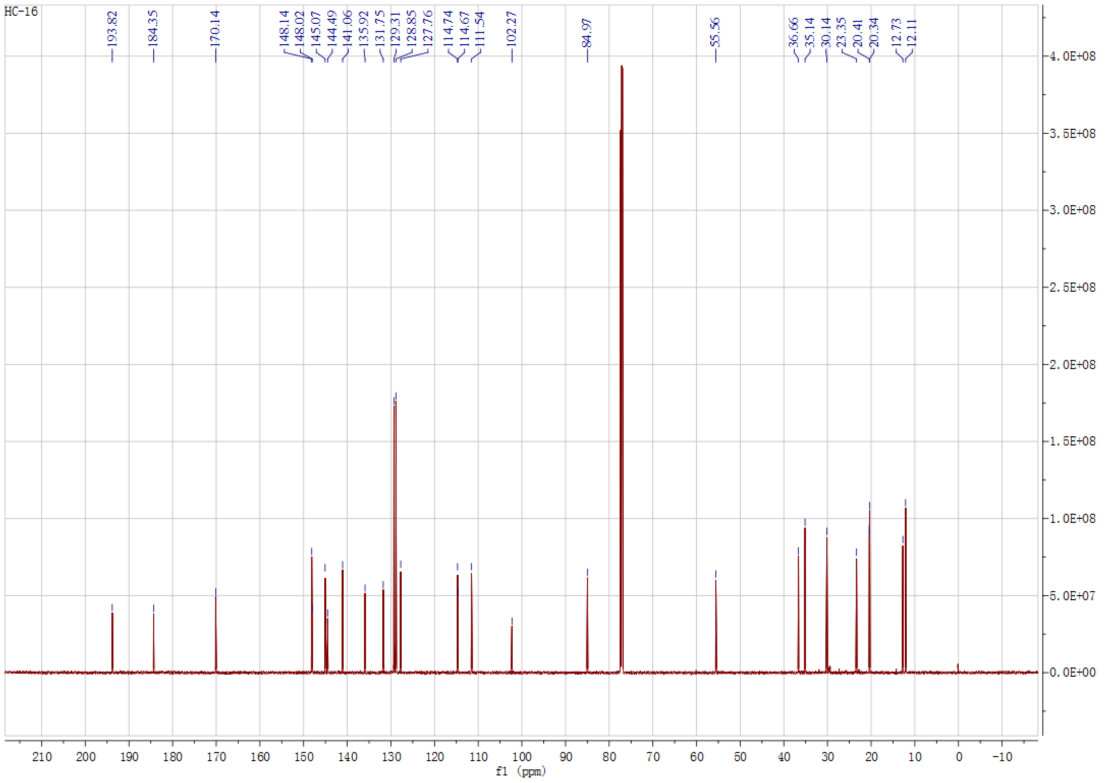

Fig.S46. ^{13}C NMR (150 MHz, $CDCl_3$) spectrum of compound 16

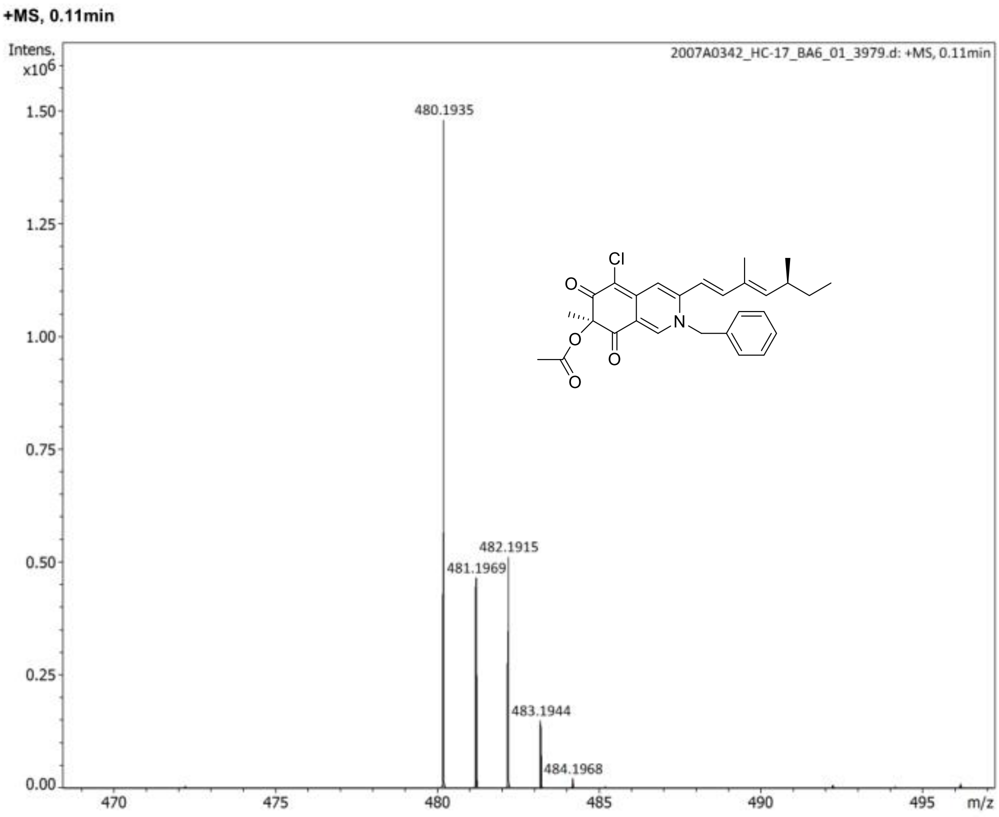

Meas. m/z	Ion Formula	m/z	z	err [ppm]	err [mDa]	mSigma	rdb	e⁻ Conf	N-Rule
480.1935	C28H31ClNO4	480.1936	1+	0.3	0.1	13.1	17.0	even	ok

Fig.S47. HRMS spectrum of compound 17

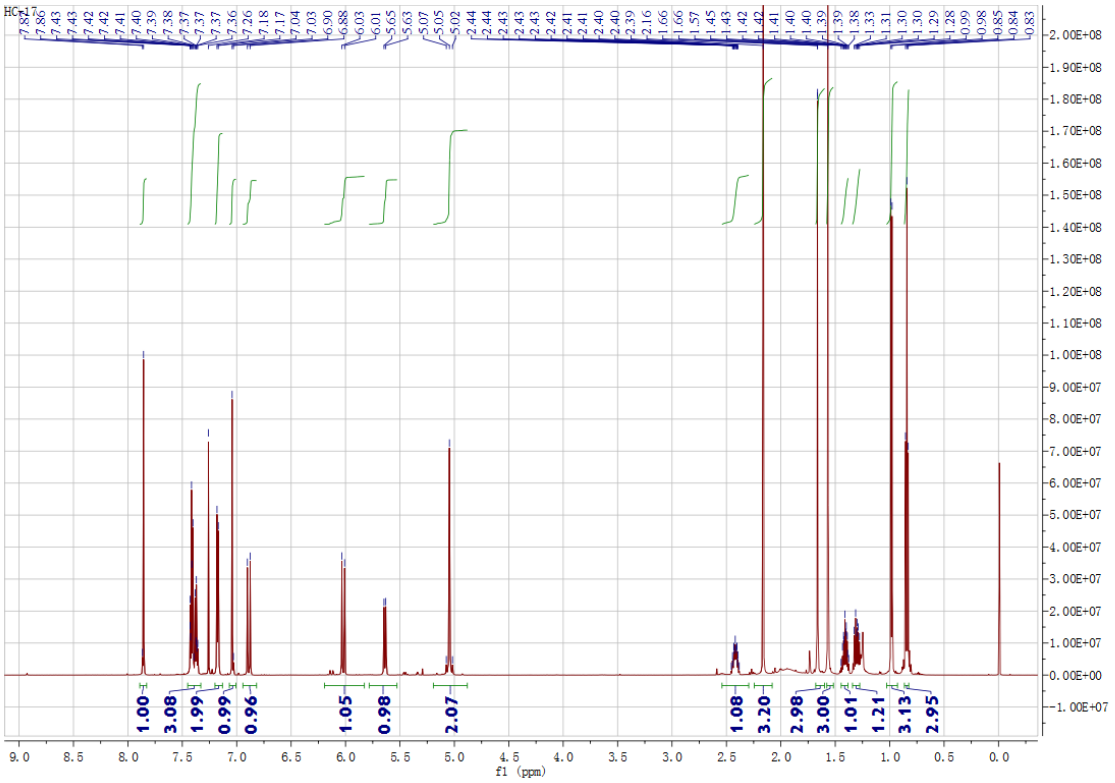

Fig.S48. ^{1}H NMR (600 MHz, $CDCl_3$) spectrum of compound **17**

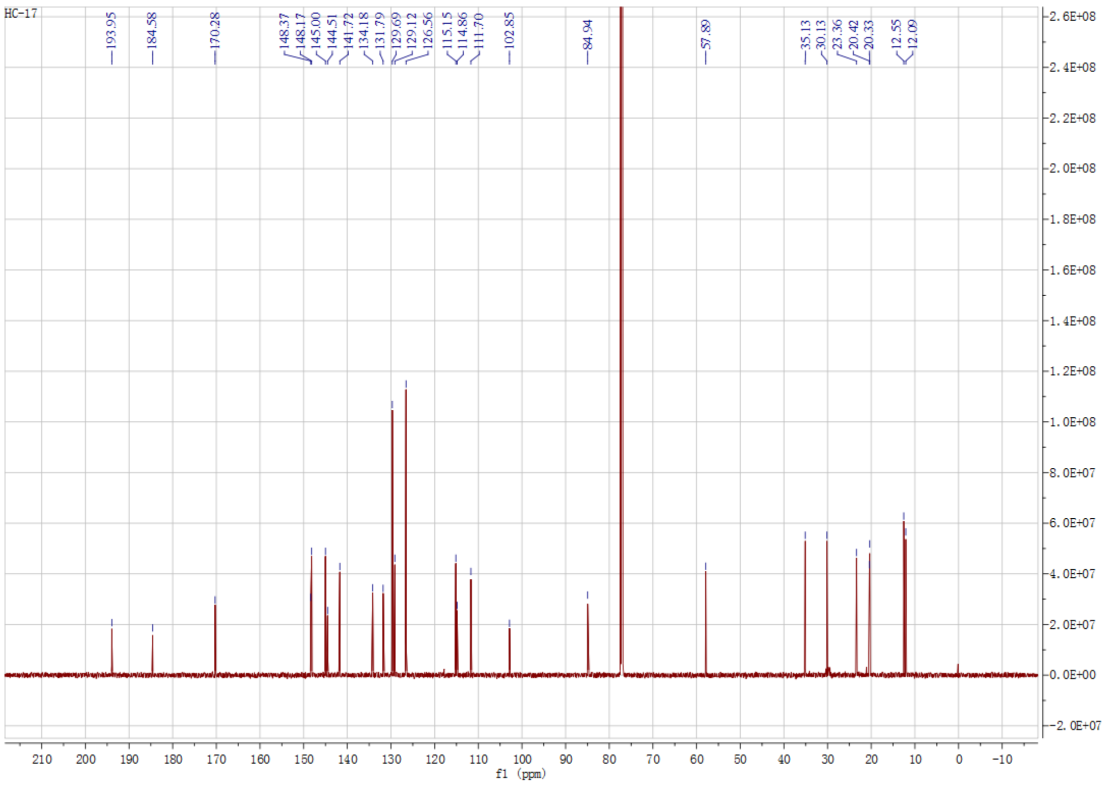

Fig.S49. ^{13}C NMR (150 MHz, CDCl3) spectrum of compound 17

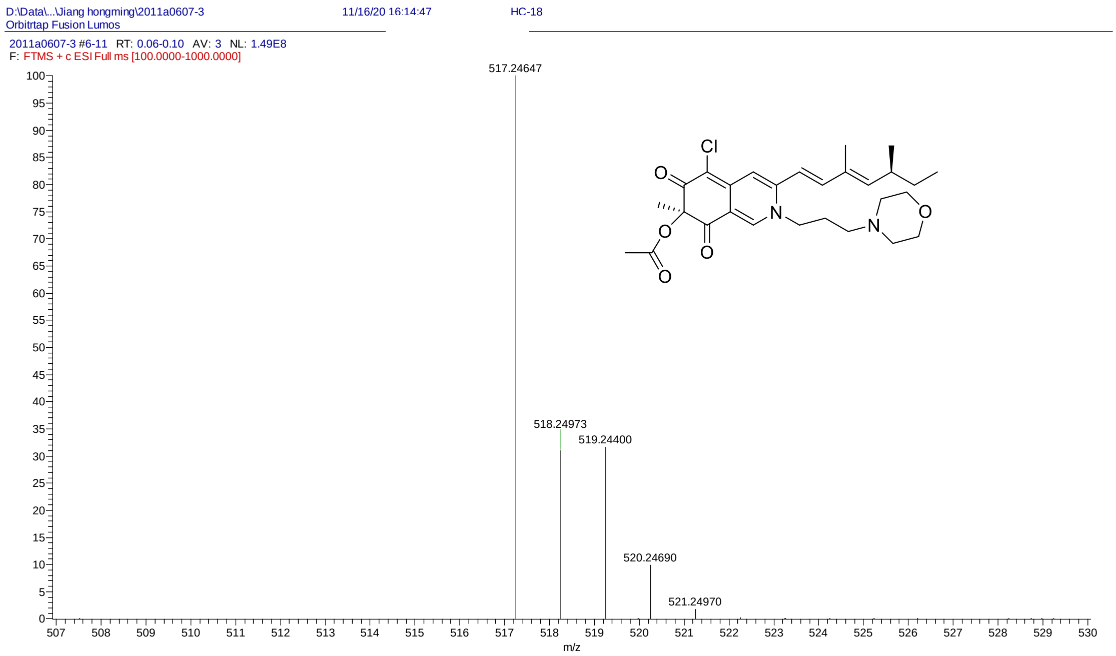

Fig.S50. HRMS spectrum of compound 18

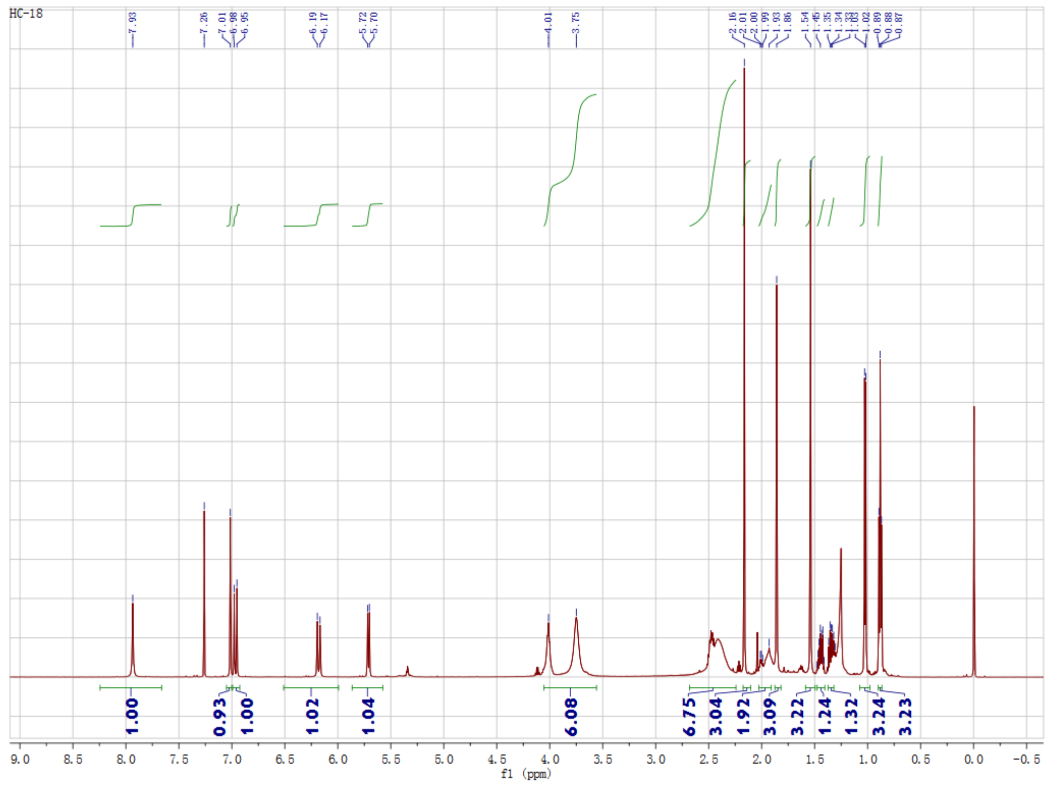

Fig.S51. ^{1}H NMR (600 MHz, $CDCl_3$) spectrum of compound **18**

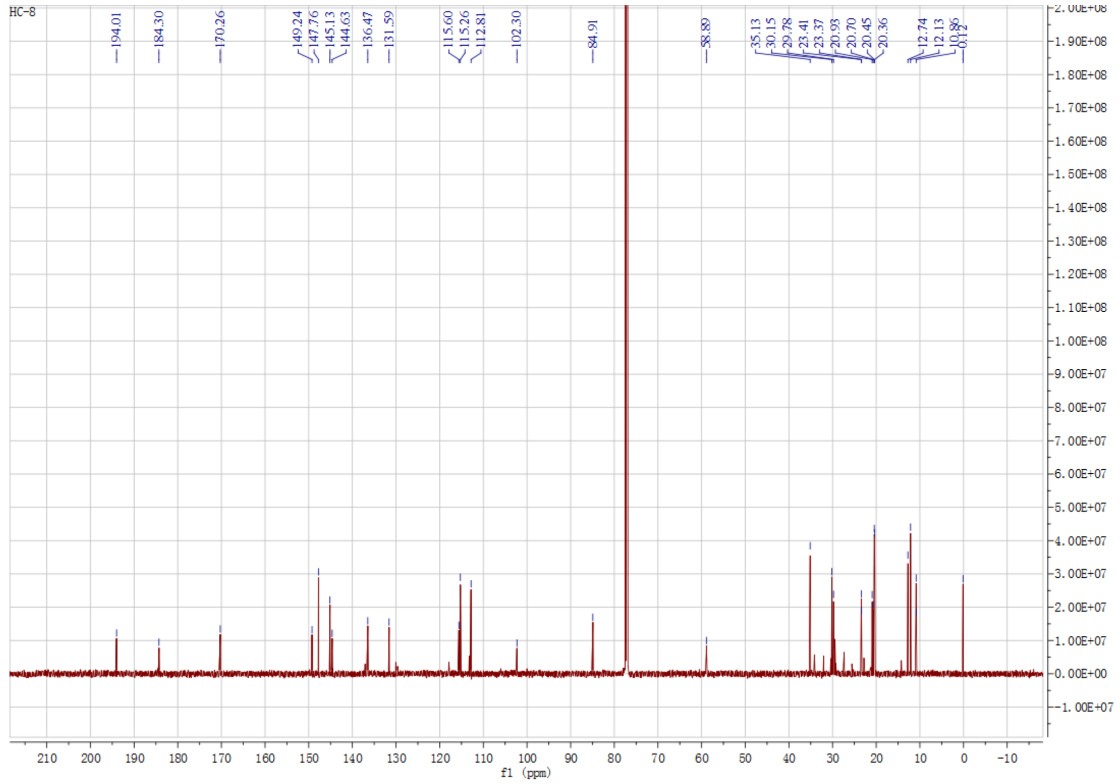

Fig.S52. ^{13}C NMR (150 MHz, $CDCl_3$) spectrum of compound 18

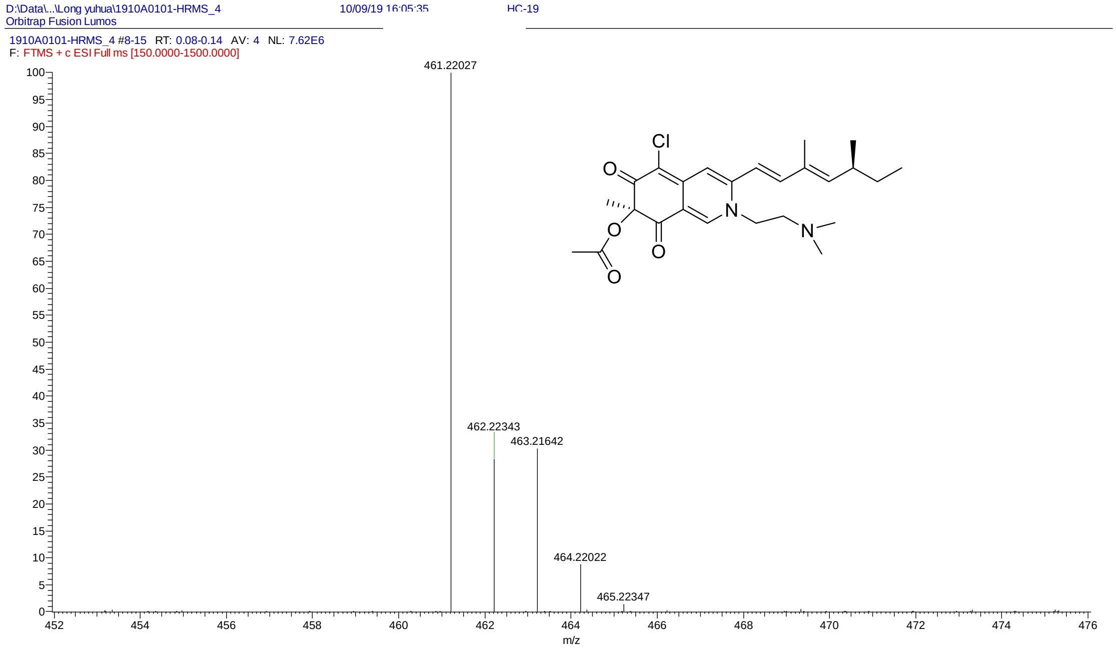

Fig.S53. HRMS spectrum of compound 19

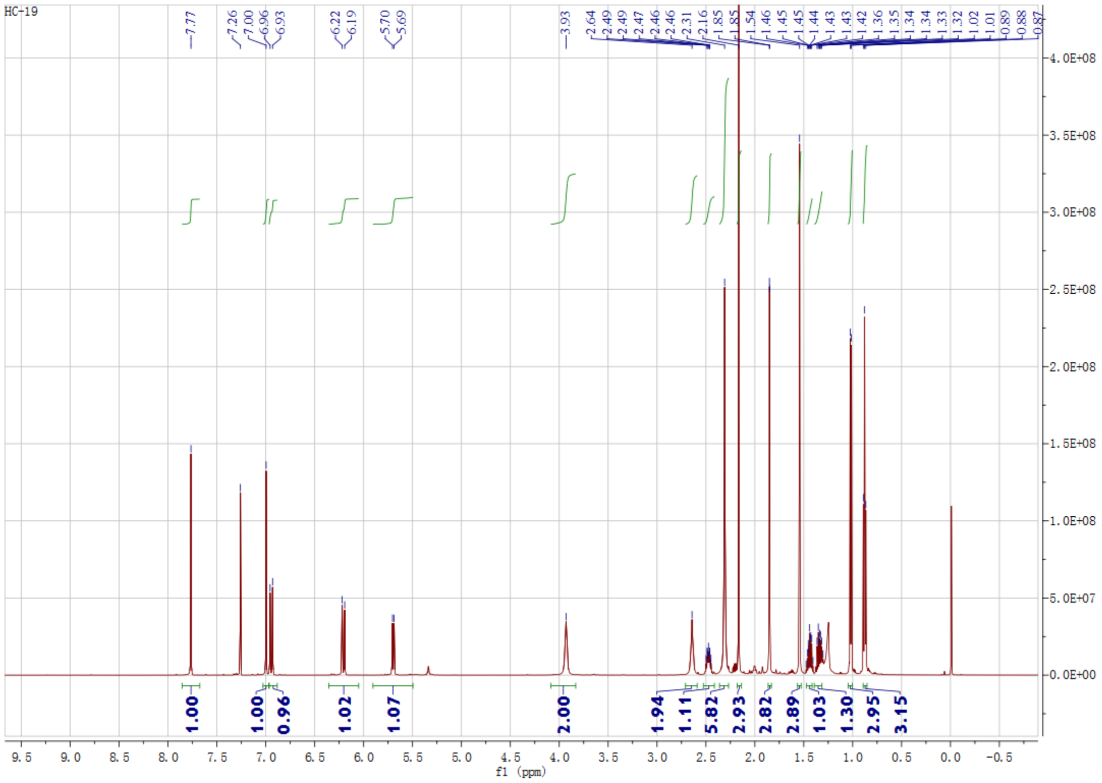

Fig.S54. ^{1}H NMR (600 MHz, $CDCl_3$) spectrum of compound **19**

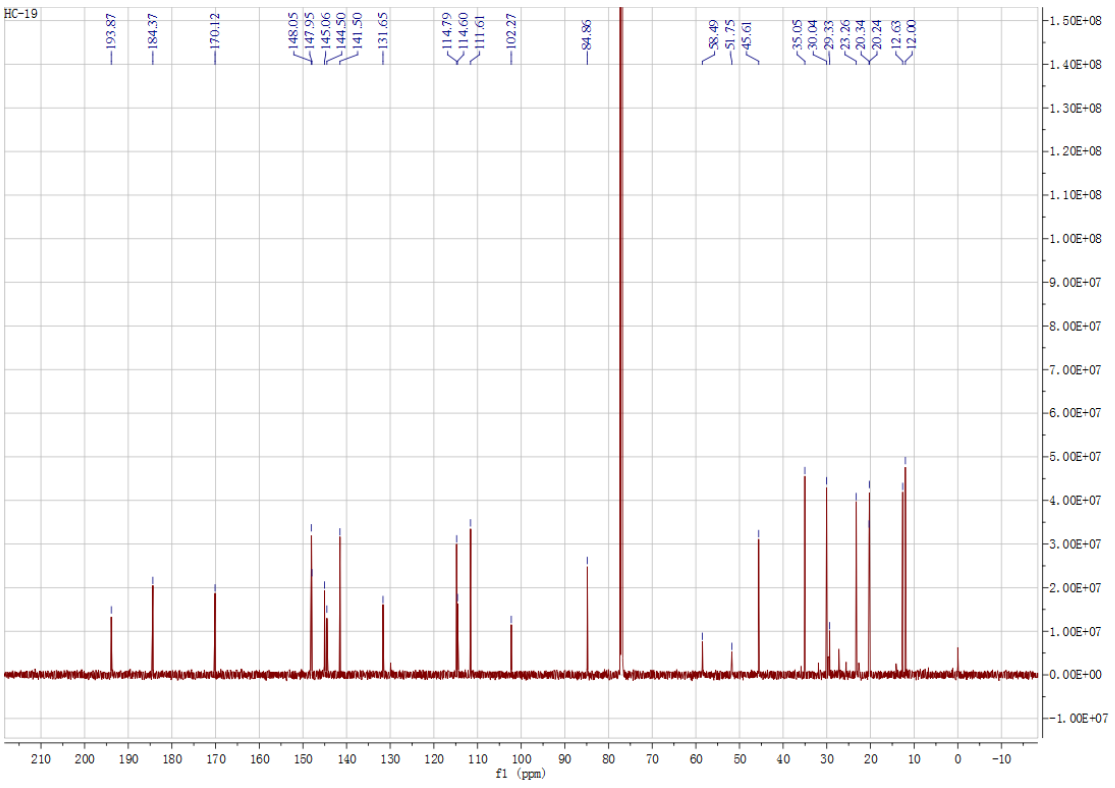

Fig.S55. ^{13}C NMR (150 MHz, $CDCl_3$) spectrum of compound 19

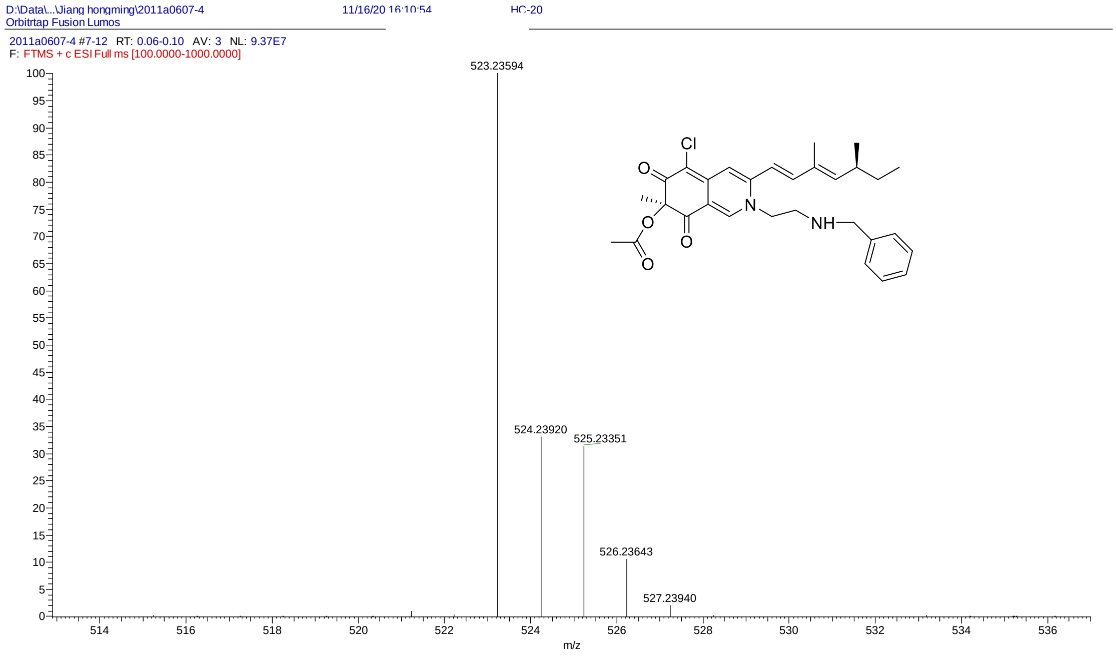

Fig.S56. HRMS spectrum of compound 20

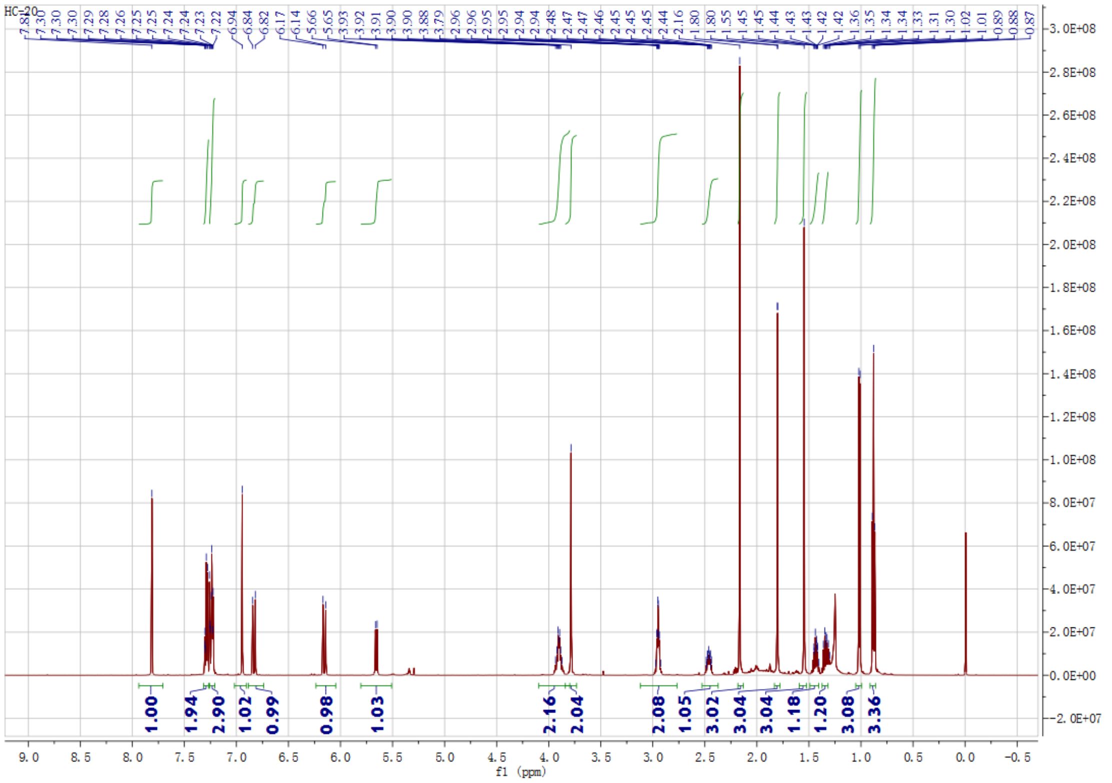

Fig.S57. ^{1}H NMR (600 MHz, CDCl3) spectrum of compound **20**

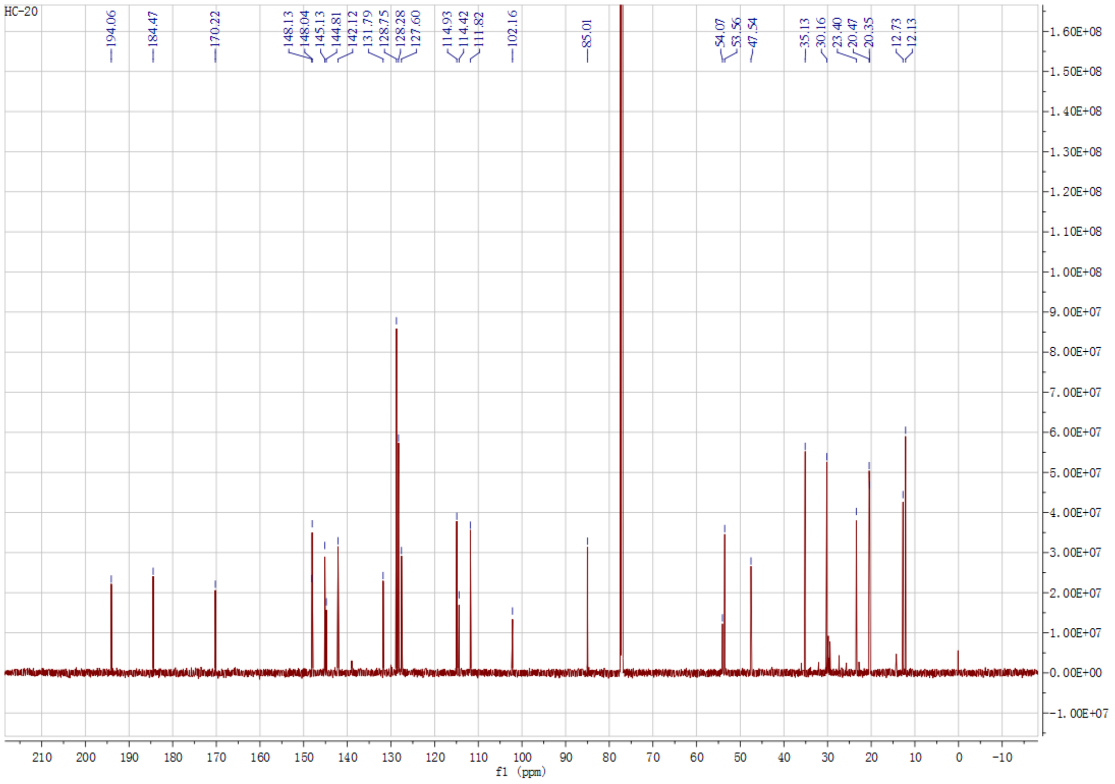

Fig.S58. ^{13}C NMR (150 MHz, CDCl3) spectrum of compound 20

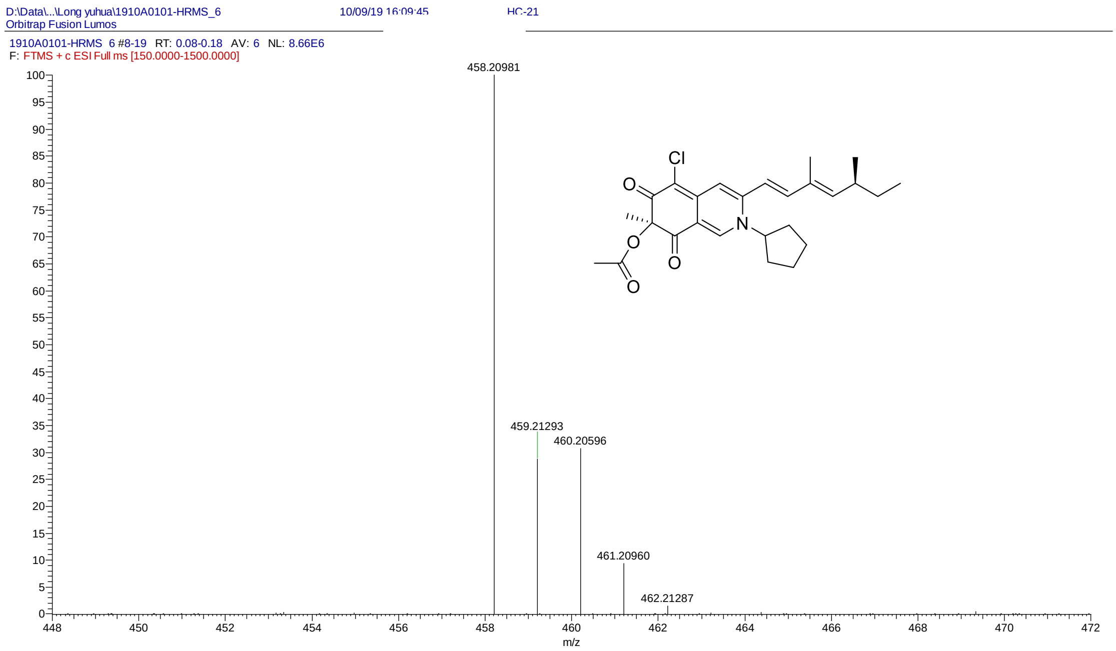

Fig.S59. HRMS spectrum of compound 21

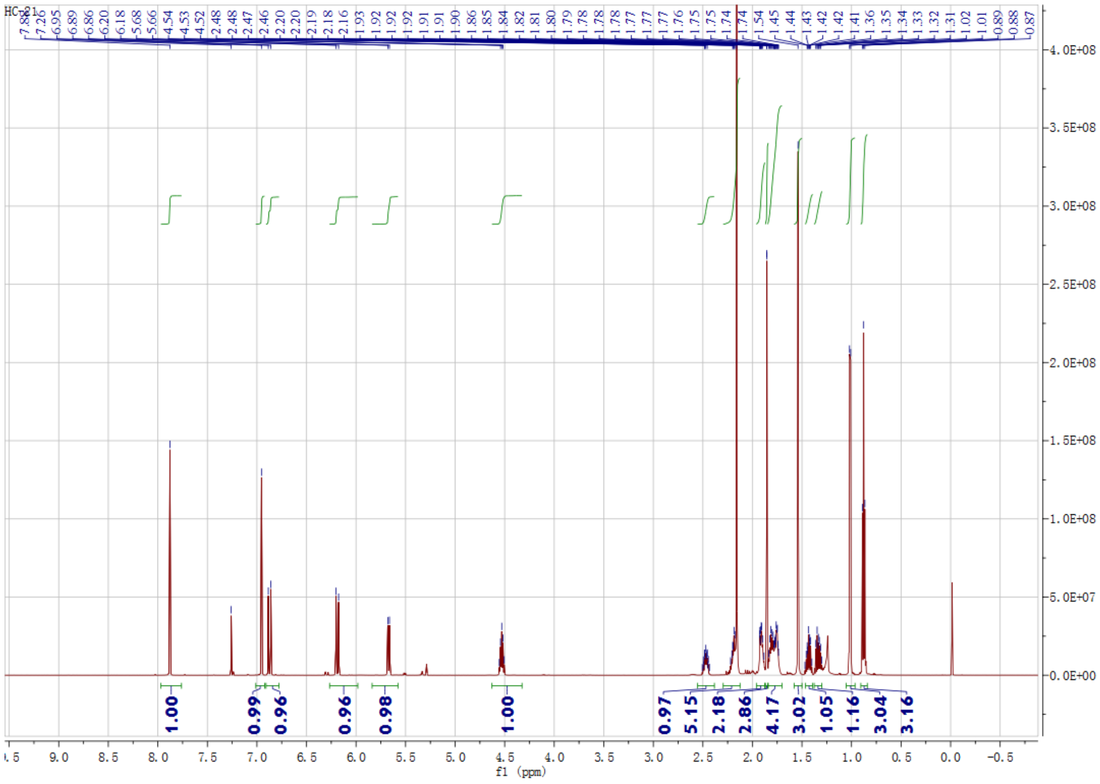

Fig.S60. ^{1}H NMR (600 MHz, $CDCl_3$) spectrum of compound **21**

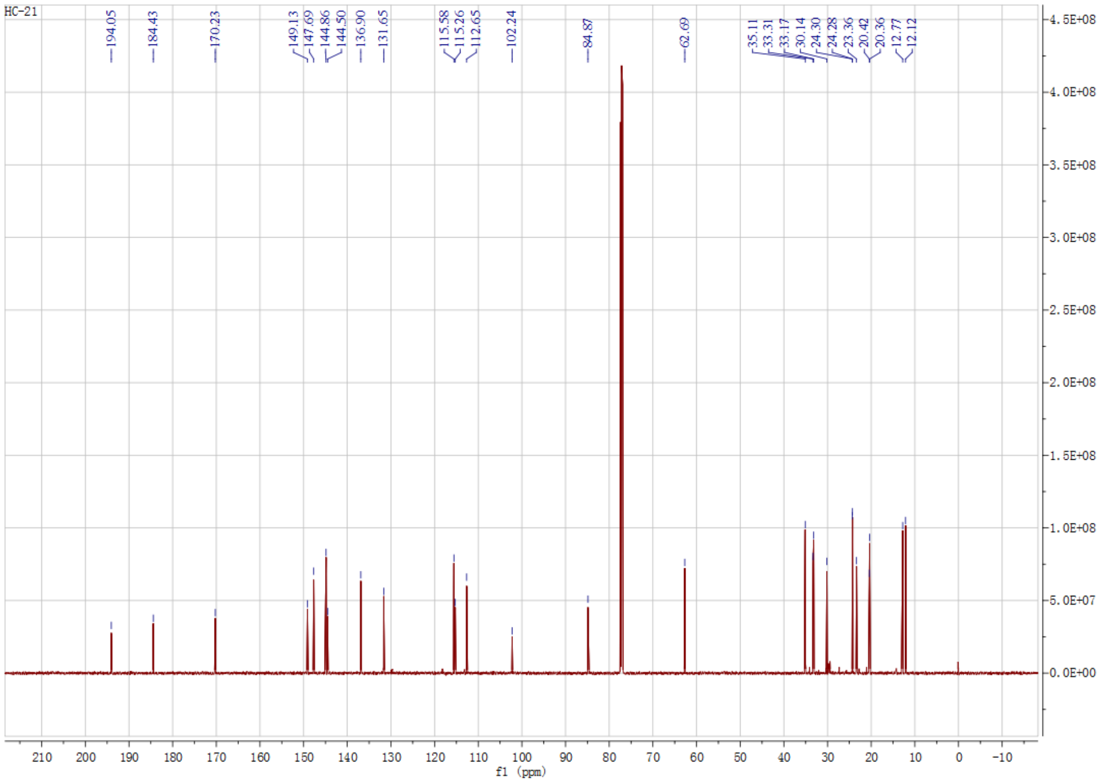

Fig.S61. ^{13}**C NMR (150 MHz, CDCl**$_3$**) spectrum of compound 21**

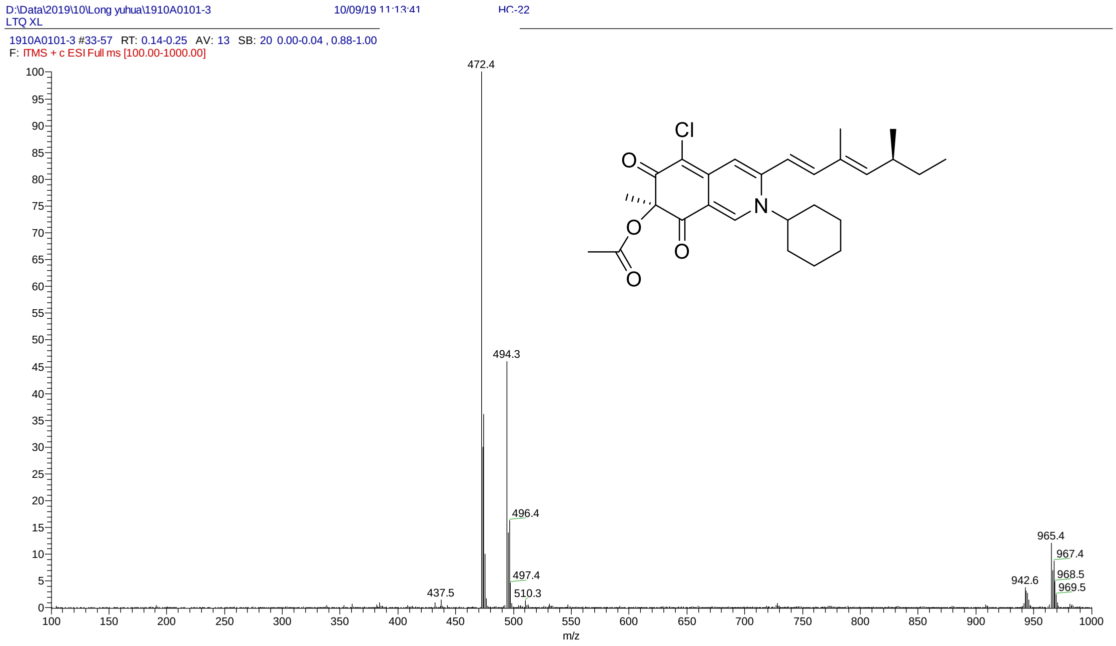

Fig.S62. MS spectrum of compound 22

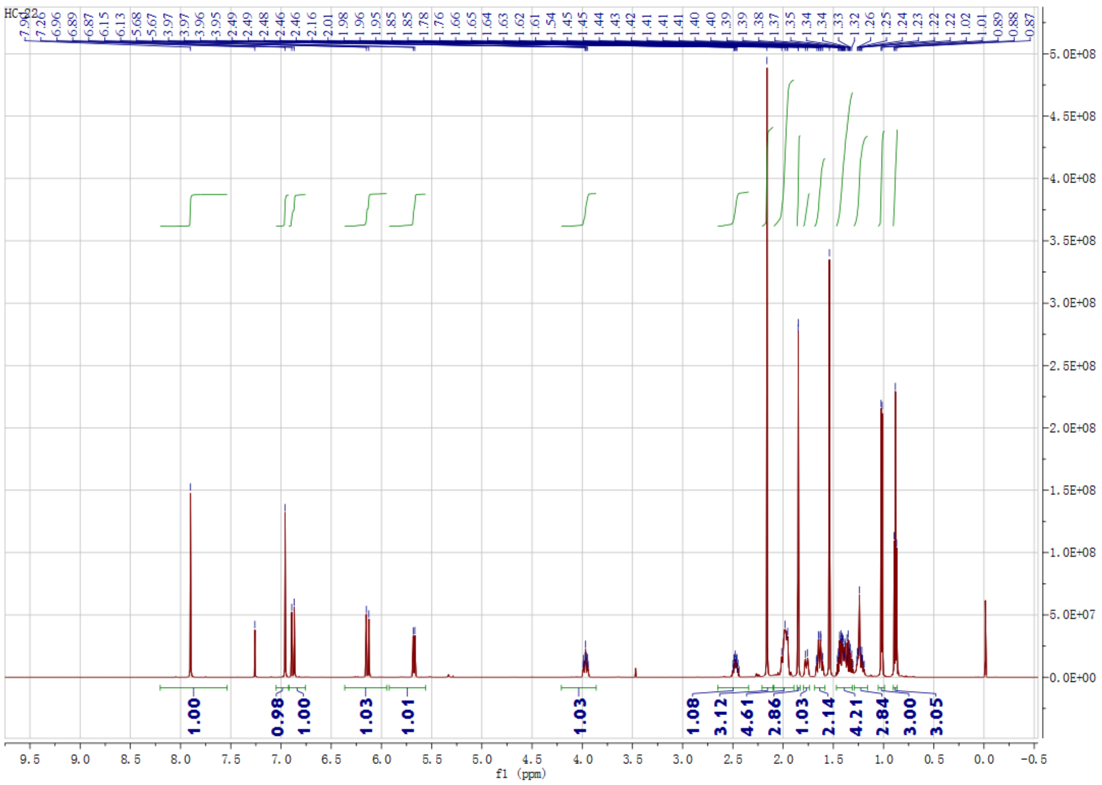

Fig.S63. ^{1}H NMR (600 MHz, $CDCl_3$) spectrum of compound **22**

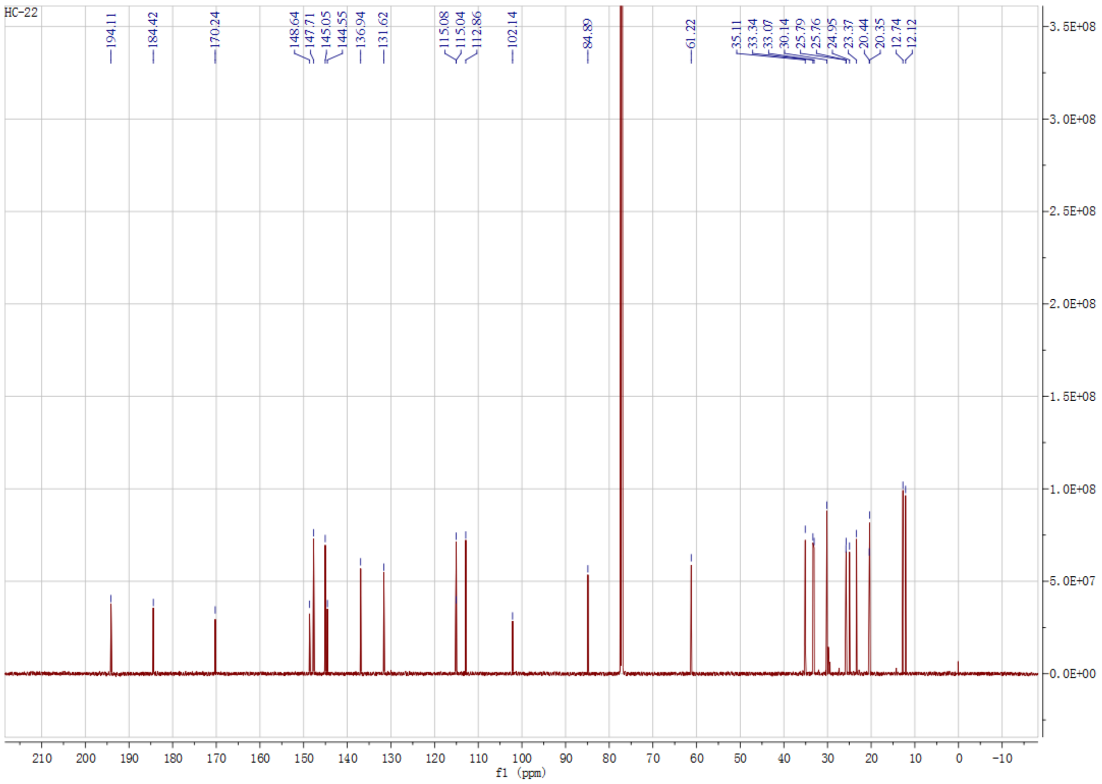

Fig.S64. ^{13}C NMR (150 MHz, $CDCl_3$) spectrum of compound 22

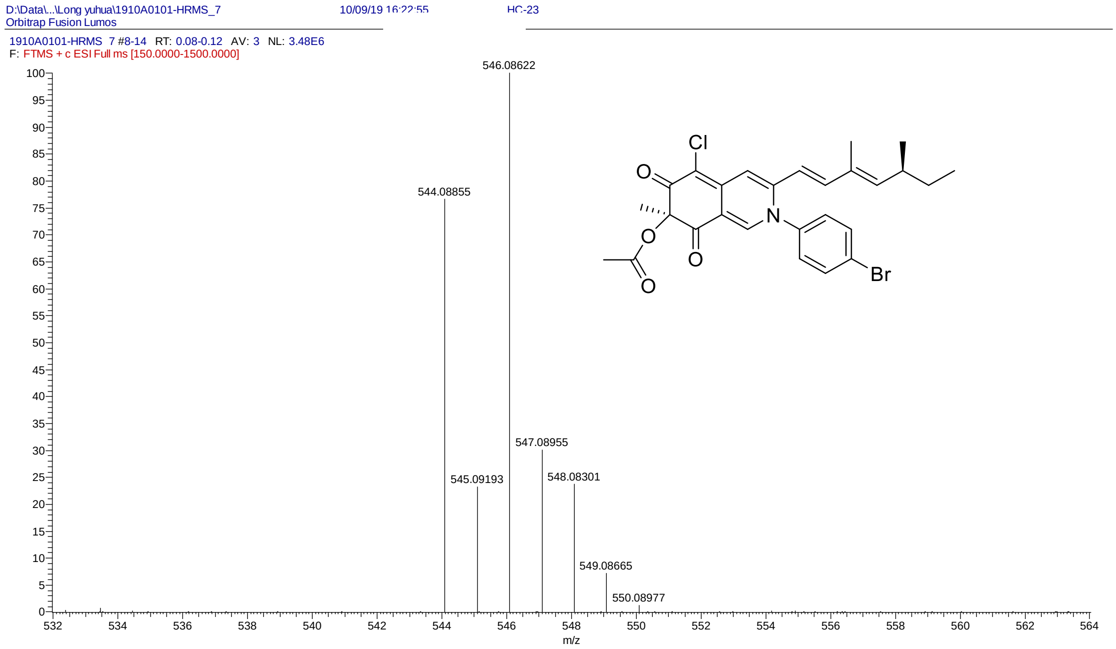

Fig.S65. HRMS spectrum of compound 23

Compound **23**

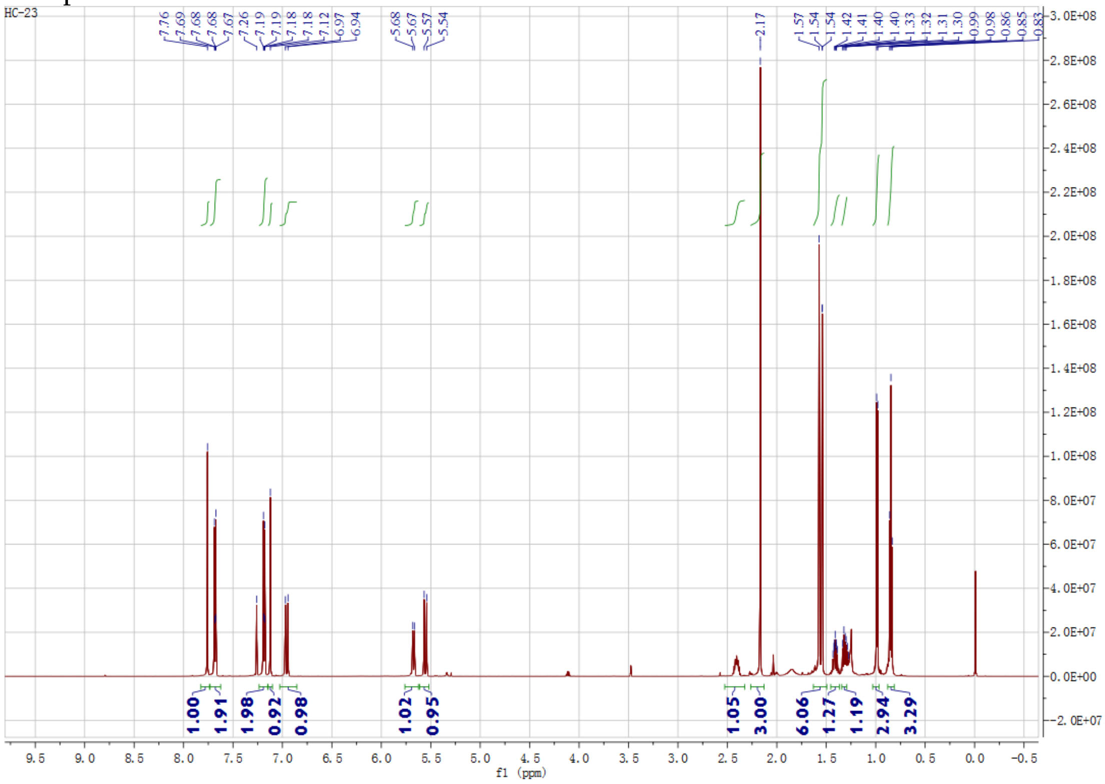

Fig.S66. ^{1}H NMR (600 MHz, $CDCl_3$) spectrum of compound **23**

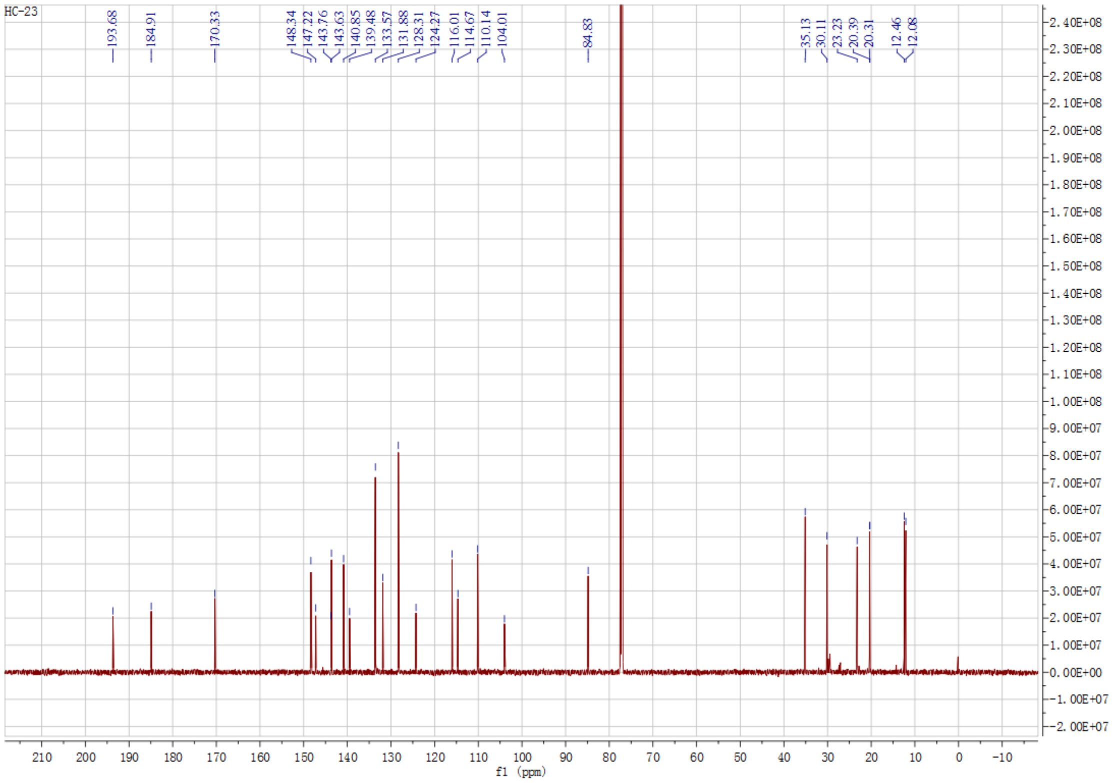

Fig.S67. ^{13}C NMR (150 MHz, $CDCl_3$) spectrum of compound 23

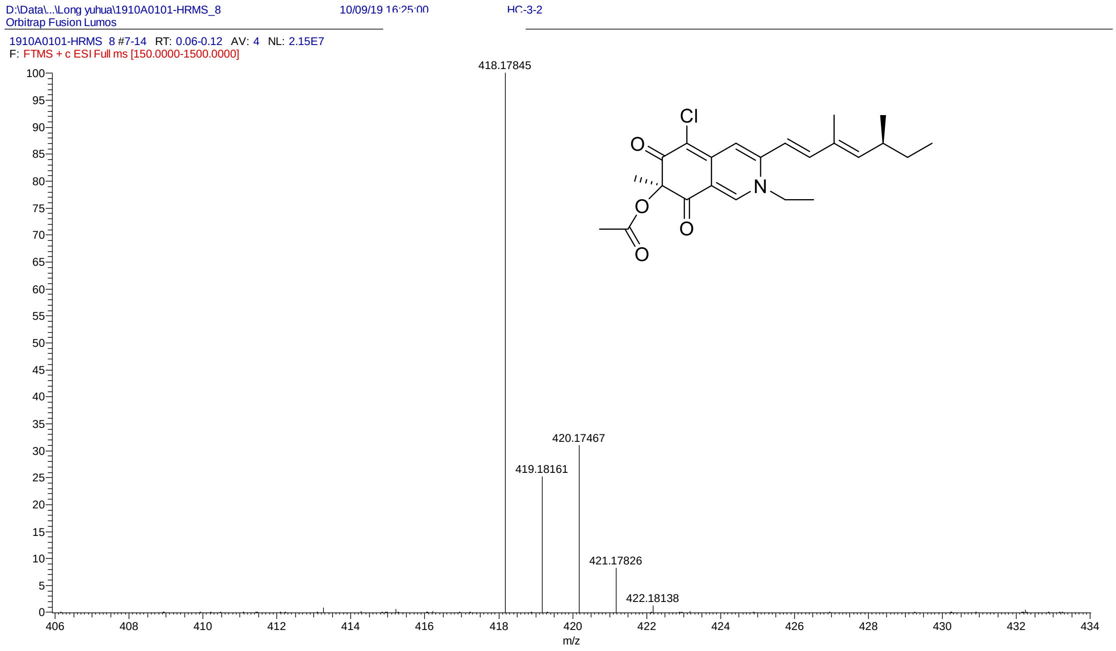

Fig.S68. HRMS spectrum of compound 24

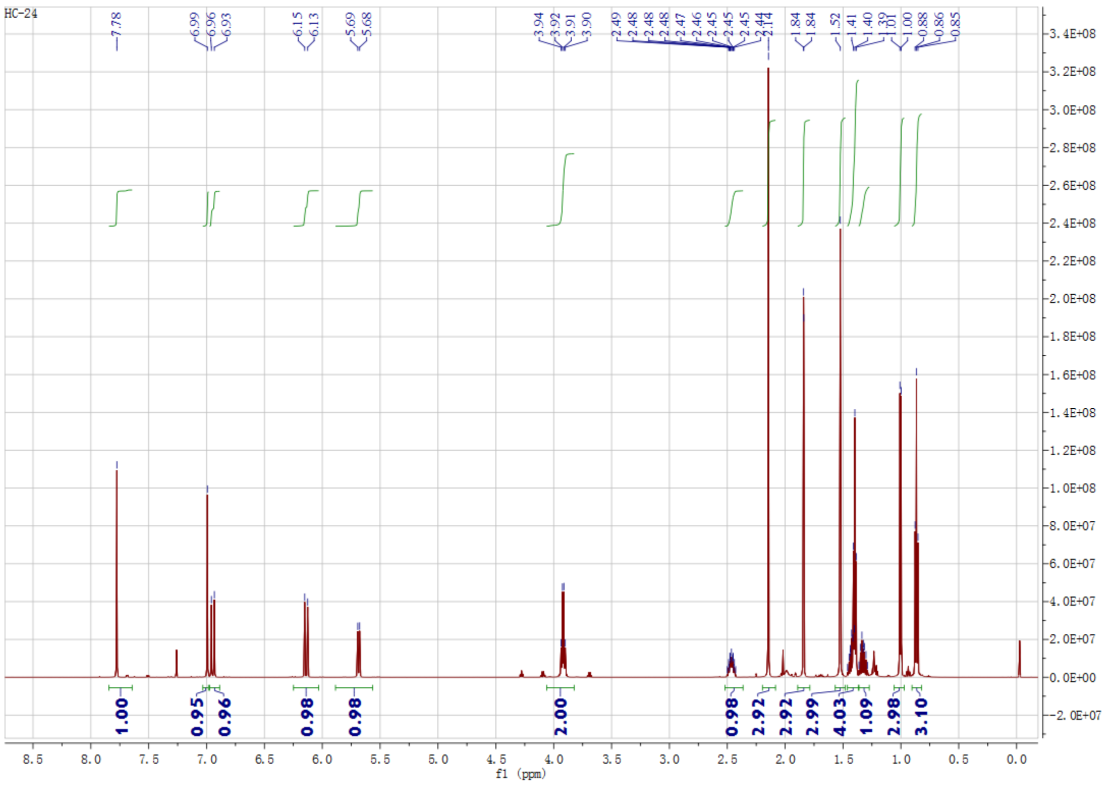

Fig.S69. ^{1}H NMR (600 MHz, CDCl3) spectrum of compound **24**

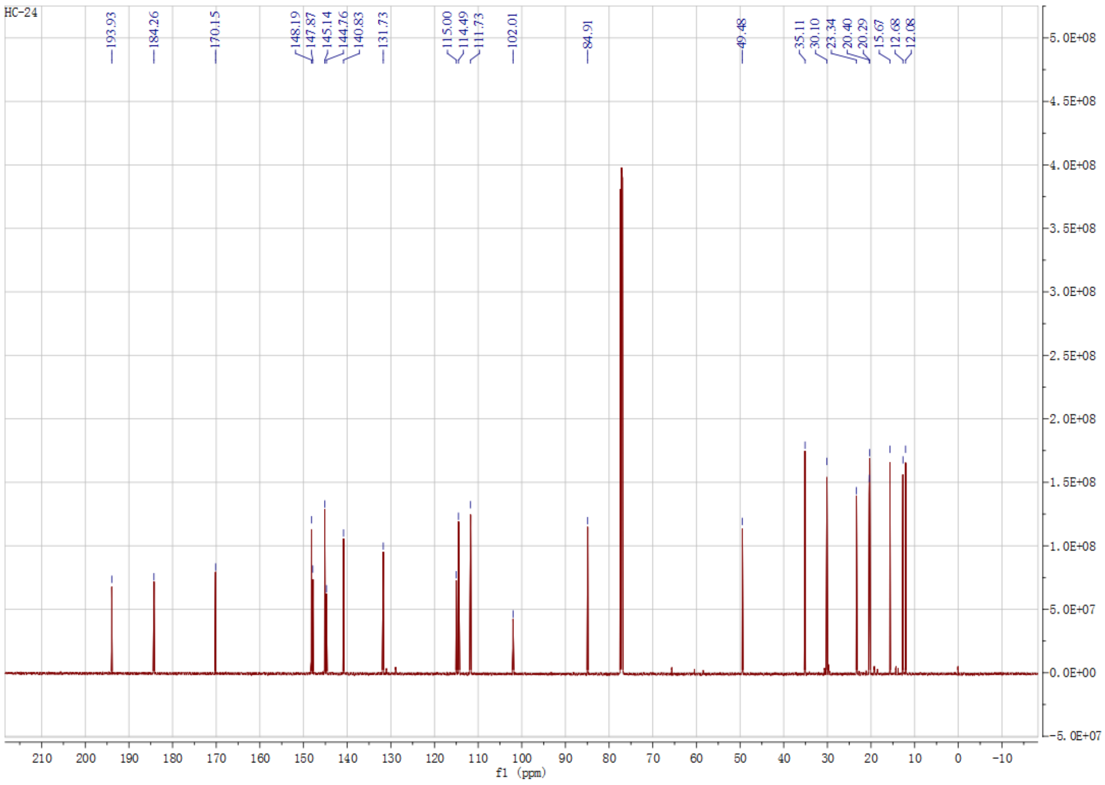

Fig.S70. [13]**C NMR (150 MHz, CDCl3) spectrum of compound 24**

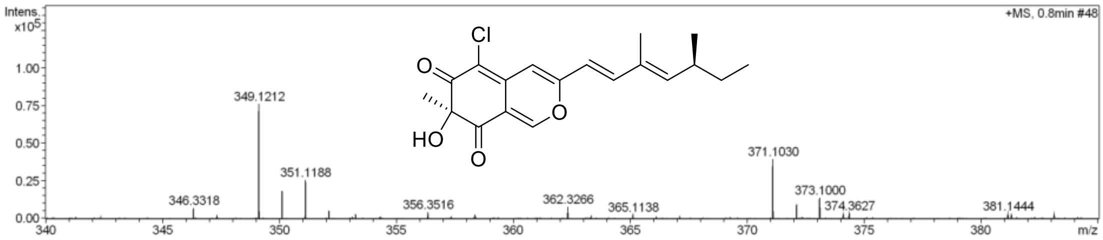

Meas. m/z	#	Ion Formula	Score	m/z	err [ppm]	err [mDa]	mSigma	rdb	e⁻ Conf	N-Rule
349.1212	1	C19H22ClO4	100.00	349.1201	-3.2	-1.1	14.8	8.5	even	ok
371.1030	1	C19H21ClNaO4	100.00	371.1021	-2.6	-1.0	14.0	8.5	even	ok
719.2164	1	C38H42Cl2NaO8	100.00	719.2149	2.0	1.5	38.5	16.5	even	ok

Fig.S71. HRMS spectrum of compound 25

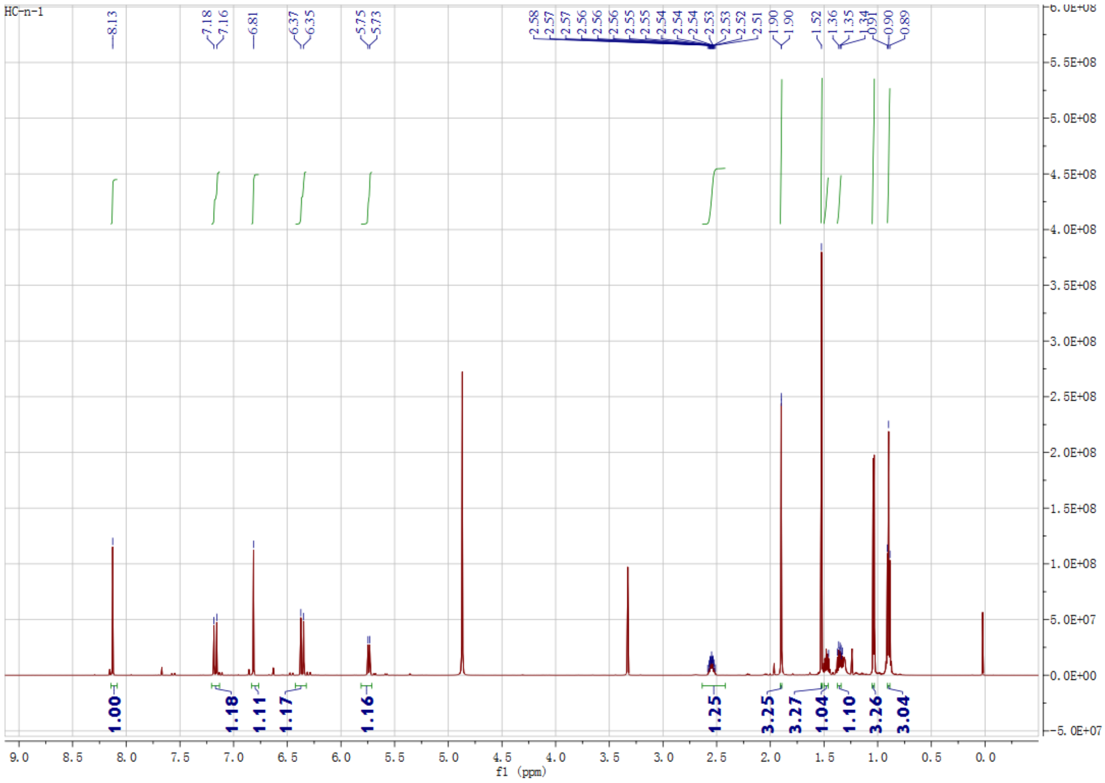

Fig.S72. ^{1}H NMR (600 MHz, $CDCl_3$) spectrum of compound **25**

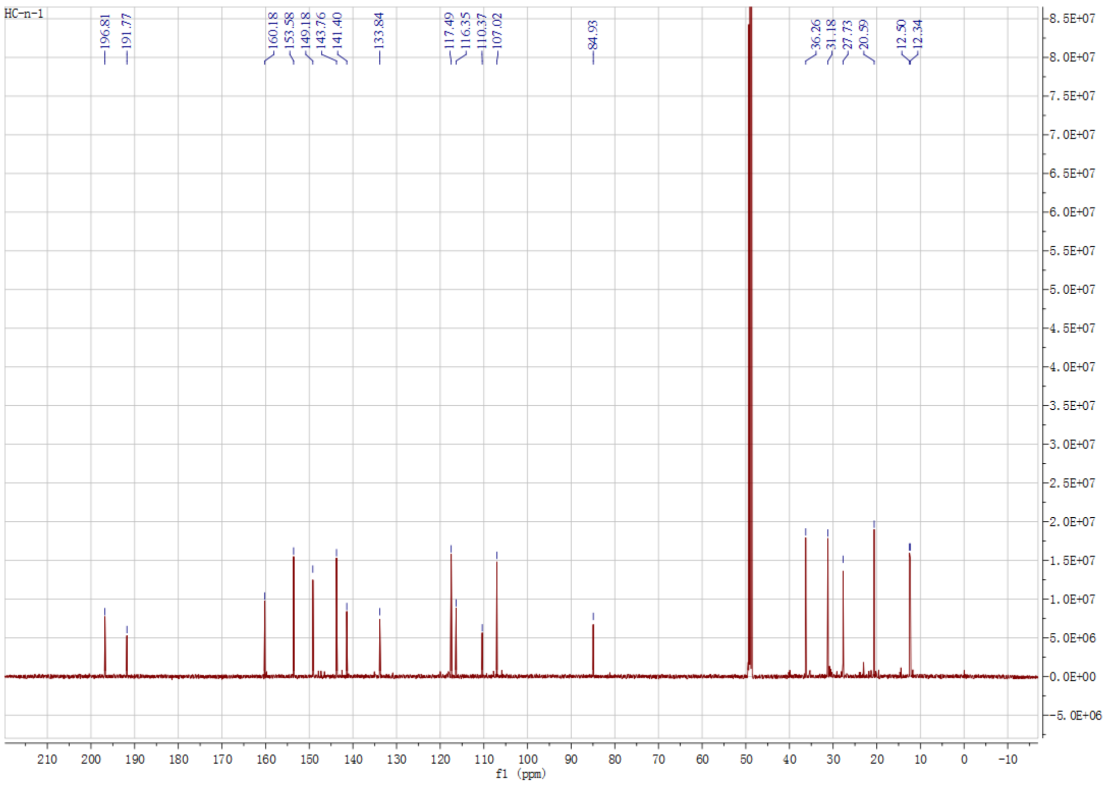

Fig.S73. ^{13}C NMR (150 MHz, CDCl3) spectrum of compound 25

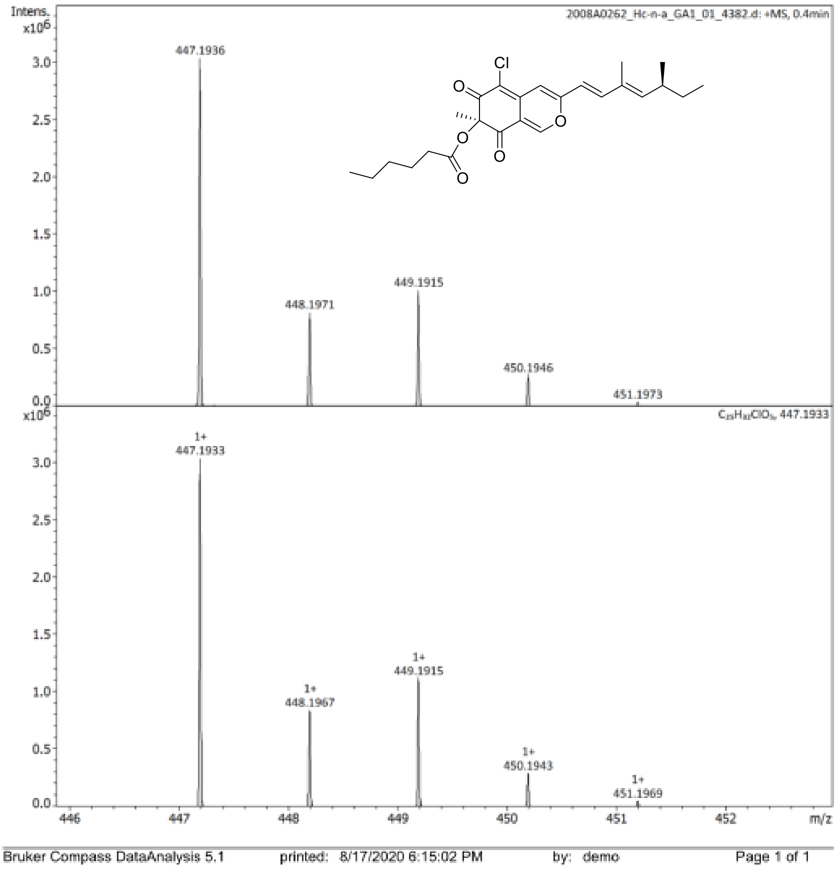

Fig.S74. HRMS spectrum of compound 26

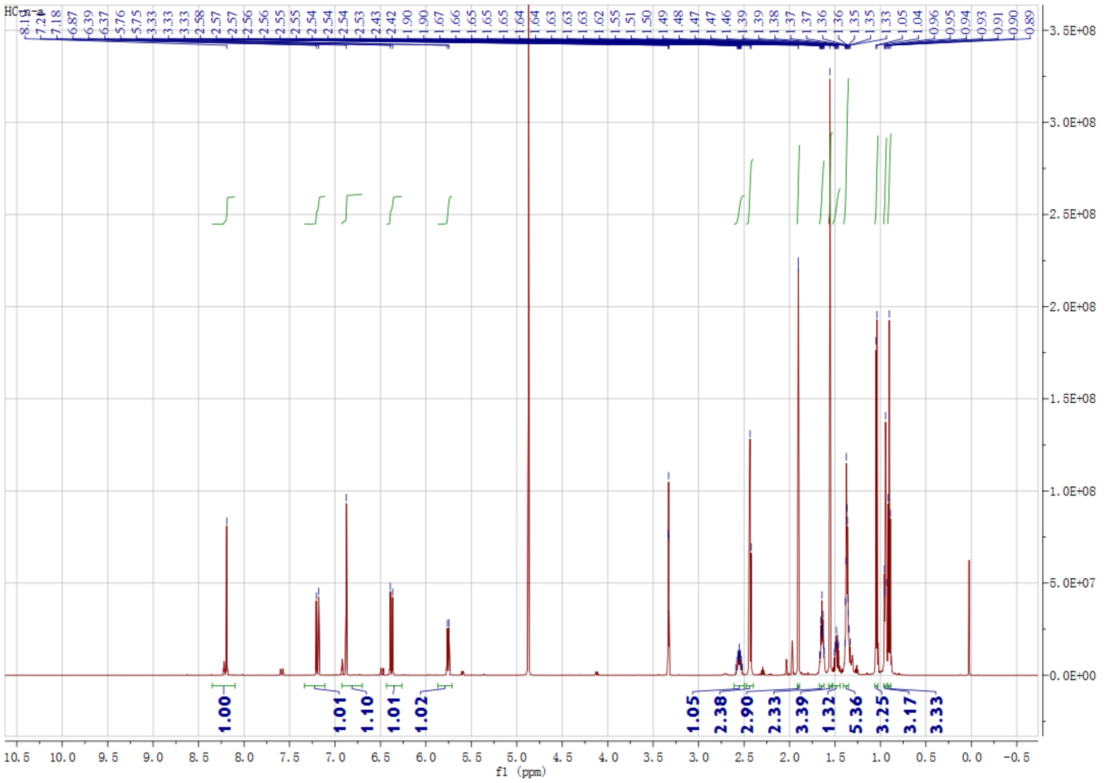

Fig.S75. ^{1}H NMR (600 MHz, $CDCl_3$) spectrum of compound **26**

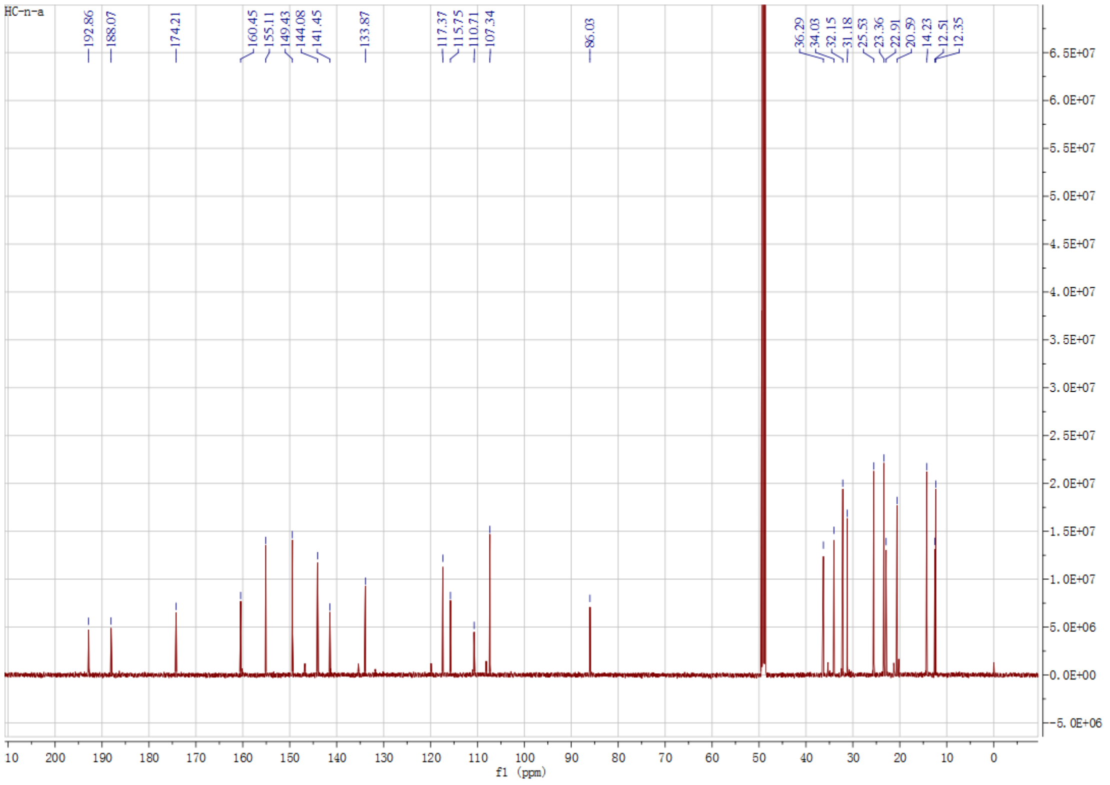

Fig.S76. ^{13}C NMR (150 MHz, $CDCl_3$) spectrum of compound 26

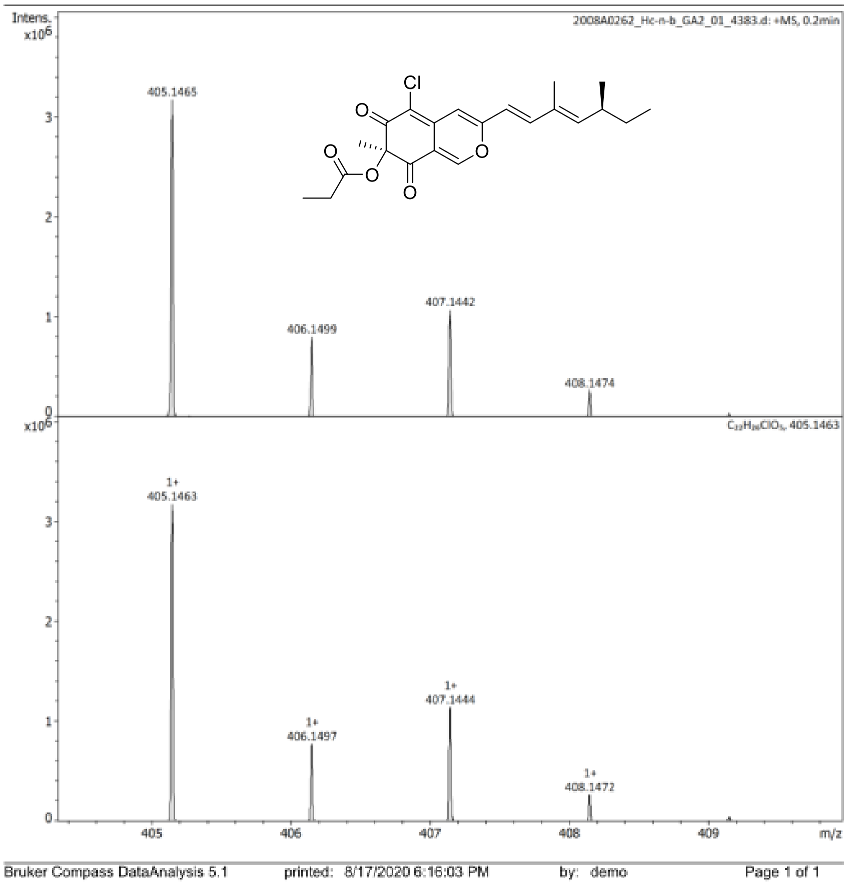

Fig.S77. HRMS spectrum of compound 27

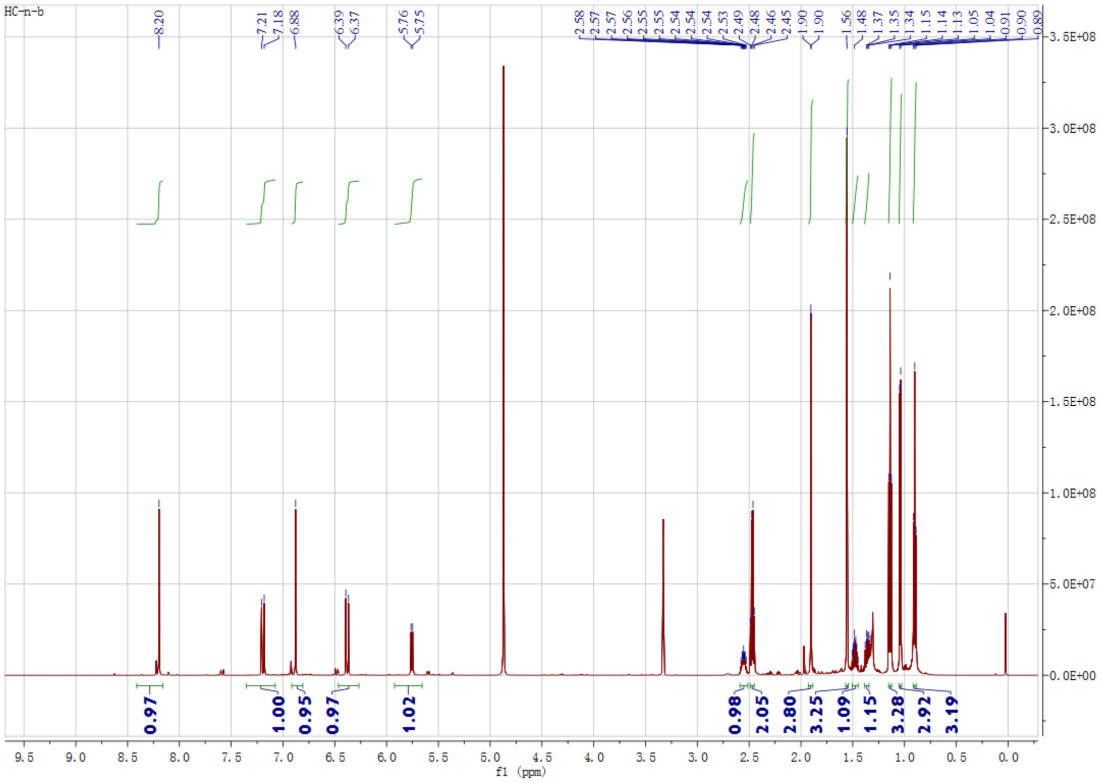

Fig.S78. ^{1}H NMR (600 MHz, $CDCl_3$) spectrum of compound **27**

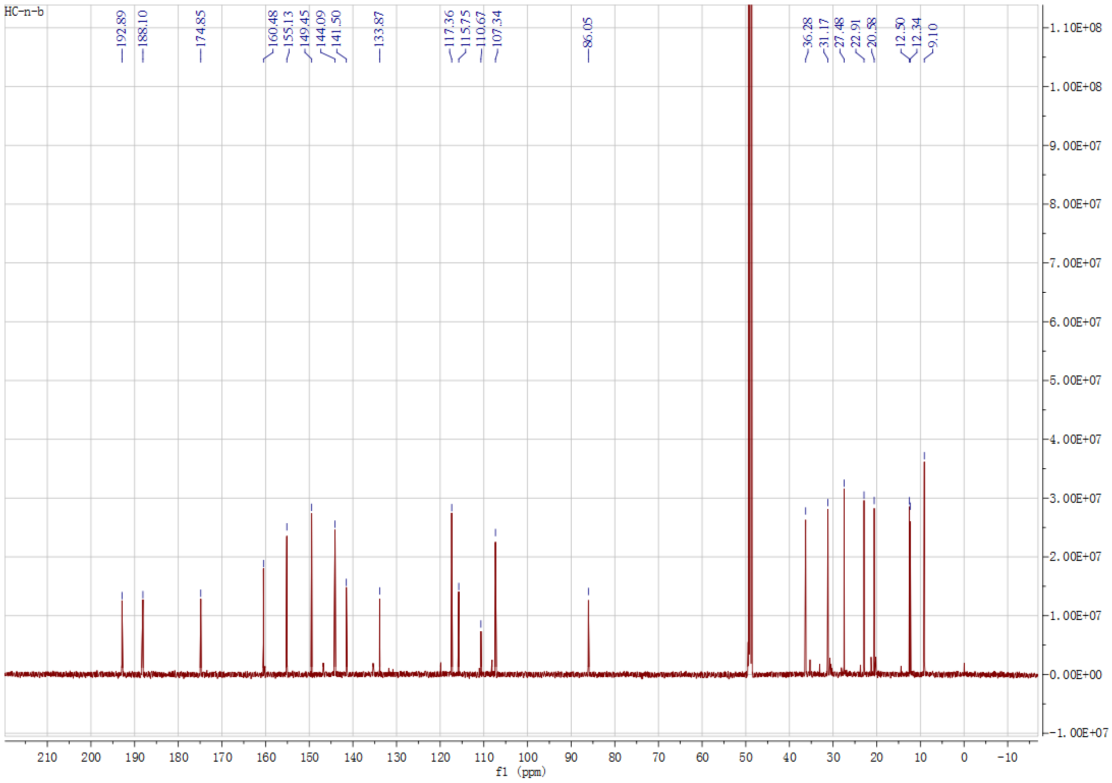

Fig.S79. ^{13}**C** NMR (150 MHz, $CDCl_3$) spectrum of compound **27**
